# The homeodomain-interacting protein kinase HPK-1 preserves protein homeostasis and longevity through master regulatory control of the HSF-1 chaperone network and TORC1-restricted autophagy in *Caenorhabditis elegans*

**DOI:** 10.1371/journal.pgen.1007038

**Published:** 2017-10-16

**Authors:** Ritika Das, Justine A. Melo, Manjunatha Thondamal, Elizabeth A. Morton, Adam B. Cornwell, Beresford Crick, Joung Heon Kim, Elliot W. Swartz, Todd Lamitina, Peter M. Douglas, Andrew V. Samuelson

**Affiliations:** 1 Department of Biomedical Genetics, University of Rochester Medical Center, Rochester, New York, United States of America; 2 Department of Biology, University of Rochester, Rochester, New York, United States of America; 3 Department of Genome Sciences, University of Washington, Seattle, Washington, United States of America; 4 Department of Cell Biology, University of Pittsburgh, Pittsburgh, Pennsylvania, United States of America; 5 Department of Molecular Biology, Hamon Center for Regenerative Science and Medicine, UT Southwestern Medical Center, Dallas, Texas, United States of America; University of California San Francisco, UNITED STATES

## Abstract

An extensive proteostatic network comprised of molecular chaperones and protein clearance mechanisms functions collectively to preserve the integrity and resiliency of the proteome. The efficacy of this network deteriorates during aging, coinciding with many clinical manifestations, including protein aggregation diseases of the nervous system. A decline in proteostasis can be delayed through the activation of cytoprotective transcriptional responses, which are sensitive to environmental stress and internal metabolic and physiological cues. The homeodomain-interacting protein kinase (*hipk*) family members are conserved transcriptional co-factors that have been implicated in both genotoxic and metabolic stress responses from yeast to mammals. We demonstrate that constitutive expression of the sole *Caenorhabditis elegans* Hipk homolog, *hpk-1*, is sufficient to delay aging, preserve proteostasis, and promote stress resistance, while loss of *hpk-1* is deleterious to these phenotypes. We show that HPK-1 preserves proteostasis and extends longevity through distinct but complementary genetic pathways defined by the heat shock transcription factor (HSF-1), and the target of rapamycin complex 1 (TORC1). We demonstrate that HPK-1 antagonizes sumoylation of HSF-1, a post-translational modification associated with reduced transcriptional activity in mammals. We show that inhibition of sumoylation by RNAi enhances HSF-1-dependent transcriptional induction of chaperones in response to heat shock. We find that *hpk-1* is required for HSF-1 to induce molecular chaperones after thermal stress and enhances hormetic extension of longevity. We also show that HPK-1 is required in conjunction with HSF-1 for maintenance of proteostasis in the absence of thermal stress, protecting against the formation of polyglutamine (Q35::YFP) protein aggregates and associated locomotory toxicity. These functions of HPK-1/HSF-1 undergo rapid down-regulation once animals reach reproductive maturity. We show that HPK-1 fortifies proteostasis and extends longevity by an additional independent mechanism: induction of autophagy. HPK-1 is necessary for induction of autophagosome formation and autophagy gene expression in response to dietary restriction (DR) or inactivation of TORC1. The autophagy-stimulating transcription factors *pha-4*/FoxA and *mxl-2*/Mlx, but not *hlh-30*/TFEB or the nuclear hormone receptor *nhr-62*, are necessary for extended longevity resulting from HPK-1 overexpression. HPK-1 expression is itself induced by transcriptional mechanisms after nutritional stress, and post-transcriptional mechanisms in response to thermal stress. Collectively our results position HPK-1 at a central regulatory node upstream of the greater proteostatic network, acting at the transcriptional level by promoting protein folding via chaperone expression, and protein turnover via expression of autophagy genes. HPK-1 therefore provides a promising intervention point for pharmacological agents targeting the protein homeostasis system as a means of preserving robust longevity.

## Author summary

Aging is the gradual and progressive decline of vitality. A hallmark of aging is the decay of protective mechanisms that normally preserve the robustness and resiliency of cells and tissues. Proteostasis is the term that applies specifically to those mechanisms that promote stability of the proteome, the collection of polypeptides that cells produce, by a combination of chaperone-assisted folding and degradation of misfolded or extraneous proteins. We have identified *hpk-1* (encoding a homeodomain-interacting protein kinase) in the nematode *C*. *elegans* as an important transcriptional regulatory component of the proteostasis machinery. HPK-1 promotes proteostasis by linking two distinct mechanisms: first by stimulating chaperone gene expression via the heat shock transcription factor (HSF-1), and second by stimulating autophagy gene expression in opposition to the target of rapamycin (TOR) kinase signaling pathway. HPK-1 therefore provides an attractive target for interventions to preserve physiological resiliency during aging by preserving the overall health of the proteome.

## Introduction

Aging is sensitive to both internal and environmental stimuli, and is tuned by multiple emergent genetic circuits. External signals include the nutritive value and quantity of the food supply; internal signals originate from discrete cellular sources, such as mitochondria or ribosomes, and discrete tissue sources, such as the reproductive system[1–7].

An overarching theme currently emerging in the aging field is one of homeostasis- homeostasis at the level of genome maintenance and gene expression[8–10], and homeostasis at the level of proteome folding and stability[11, 12]. The gradual loss of homeostasis, from precision of gene expression to protein folding and degradation is a common hallmark of aging organisms. Therefore, longevity is often extendable by manipulations that increase overall stress resistance, such as thermal shock or hypoxia, by a phenomenon known as hormesis. It is generally believed that hormesis extends longevity by bolstering organismal and cellular stress response pathways, which subsequently offsets aging-related decline in these pathways[13].

A major aim in aging research is to improve quality-of-life with advancing age (often referred to as healthspan) by reinforcement of those maintenance pathways that ensure the integrity of biological processes[6]. Indeed, a number of aging-related diseases, such as Alzheimer’s and Parkinson’s dementias, are believed to arise from the decline in the systems that maintain proteome stability and plasticity, by injury to or defects in the cellular processes that promote accurate protein folding and elimination of misfolded and damaged proteins[14]. Maintaining protein homeostasis (proteostasis) is the collective process that preserves a robust and functional proteome; an equation balanced by rates of protein synthesis, protein folding, and protein turnover. Protein synthesis places stress on the proteome by increasing the total concentration of cellular protein. Protein concentrations within the cell can approach saturation levels achieved within crystals[15]. Thus, a major challenge in maintaining proteostasis is an issue of solubility, which is managed by molecular chaperones. Chaperones play an integral role both in assisting in the correct maturation of nascent polypeptides, and in the elimination of proteins through chaperone-mediated degradative pathways. Cells eliminate misfolded, damaged or unneeded polypeptides by ubiquitin-mediated proteosomal degradation as well as by macroautophagy (hereafter referred to as autophagy) at the lysosome. Yet, chaperones have a limited buffering capacity to maintain proper folding under different forms of cellular stress. Thus potent stress response mechanisms act to resolve both acute and chronic stress to the proteome, through refolding, degradation, and sequestration.

Protein homeostatic mechanisms are regulated at the transcriptional and post-transcriptional levels. For instance, the heat shock transcription factor HSF-1 activates transcription of the chaperone genetic network in response to a wide range of stresses, the most well-known being acute thermal stress[16]. A myriad of transcription factors in *C*. *elegans* have been shown to promote autophagy at the level of gene expression and autophagosome formation in response to various environmental stressors[17]; including FOXA (PHA-4)[18], TFEB (HLH-30)[19, 20], Mondo/Mlx (MML-1/MXL-2)[21], the HNF4-related nuclear hormone receptor (NHR-62)[22], and several transcription factors necessary for ER and mitochondrial unfolded protein responses[23–25]. There is a growing body of evidence that demonstrates that the loss of autophagy and the decline of proteostasis are conserved hallmarks of normal aging[12, 26, 27]. Consistently, during aging there is a general deterioration in the ability of cells to activate these transcriptional responses to proteotoxic stress. However, there is evidence in *C*. *elegans* suggesting that normal cell non-autonomous signals impair proteostasis. For example, under normal conditions, thermosensory neurons inhibit the ability of distal tissues to resolve proteotoxic stress mediated by polyglutamine expression[28], and the onset of reproduction triggers both a rapid decline in protein quality control in the soma[29] and chromatin silencing at stress response genes limits the somatic heat shock response[30]. In mammals, the autophagy system antagonizes the progression of multiple neurodegenerative disorders[31]. Thus, identifying signals that either positively or negatively impact the inducibility of proteostatic mechanisms, as well as how they are regulated and coordinated will be essential for the treatment of age-associated proteotoxic disease and to maximize healthy aging.

In this study, we describe the *C*. *elegans* homolog of the HIPK homeodomain-interacting protein kinase, or HPK-1, as an essential co-factor of multiple transcriptional responses that collectively preserve proteostasis. The Hipk gene family encodes a set of conserved kinases that act as transcriptional co-factors important for the regulation of cell growth, development, differentiation and apoptosis[32, 33]. Hipks are activated by metabolic and genotoxic stressors from yeast to mammals[34–37]. In a previous study we found that *hpk-1* is necessary for wild-type lifespan and the extended longevity of insulin signaling mutants[38]. Here we show that HPK-1 reinforces proteostasis in *C*. *elegans* in response to both thermal stress and dietary restriction by transcriptional activation of chaperone and autophagy pathways, respectively. We show that HPK-1 is necessary for chaperone induction by HSF-1 in response to heat stress and opposes sumoylation of HSF-1, an inhibitory post-translational modification in mammals[39–41]. Consistently, we show that RNAi of the SUMO moiety encoded by *smo-1* enhances HSF-1 dependent chaperone induction after heat stress. Thus, we propose that HPK-1 preserves HSF-1 activity in *C*. *elegans* by inhibiting sumoylation. As a separate output of HPK-1, we showed that HPK-1 (but not HSF-1) is necessary for induction of autophagosome formation and autophagy gene expression in response to dietary deprivation and reduced TORC1 signaling. HPK-1 overexpression both extended longevity and conferred protection against polyQ protein aggregate formation and toxicity. The ability of *hpk-1* to increase lifespan and prevent protein aggregation relies on PHA-4(FoxA) and MXL-2(Mlx), Thus, HPK-1 functions as a regulatory hub for multiple transcription factors with proteostasis-preserving activities.

## Results

### The homeodomain interacting protein kinase HPK-1 extends longevity

We initially identified the *hpk-1* gene in an RNAi screen aimed at identifying genes necessary for the extension of longevity in *daf-2(e1370)* mutant animals. The *hpk-1* gene represented an attractive target for subsequent investigation because (1) members of the HIPK gene class are known to have broad physiological roles in transcription factor regulation in response to nutrient availability and other environmental cues, collectively suggesting a central role for HIPKs in stress response pathways across eukaryotes, and (2) initial characterization in our laboratory revealed that *hpk-1* promotes the global maintenance of proteostasis by protecting animals from the formation of age-associated polyglutamine protein aggregates, and one of the phenotypic hallmarks of aging organisms is a gradual and progressive decline in proteostasis.

**Fig 1:**
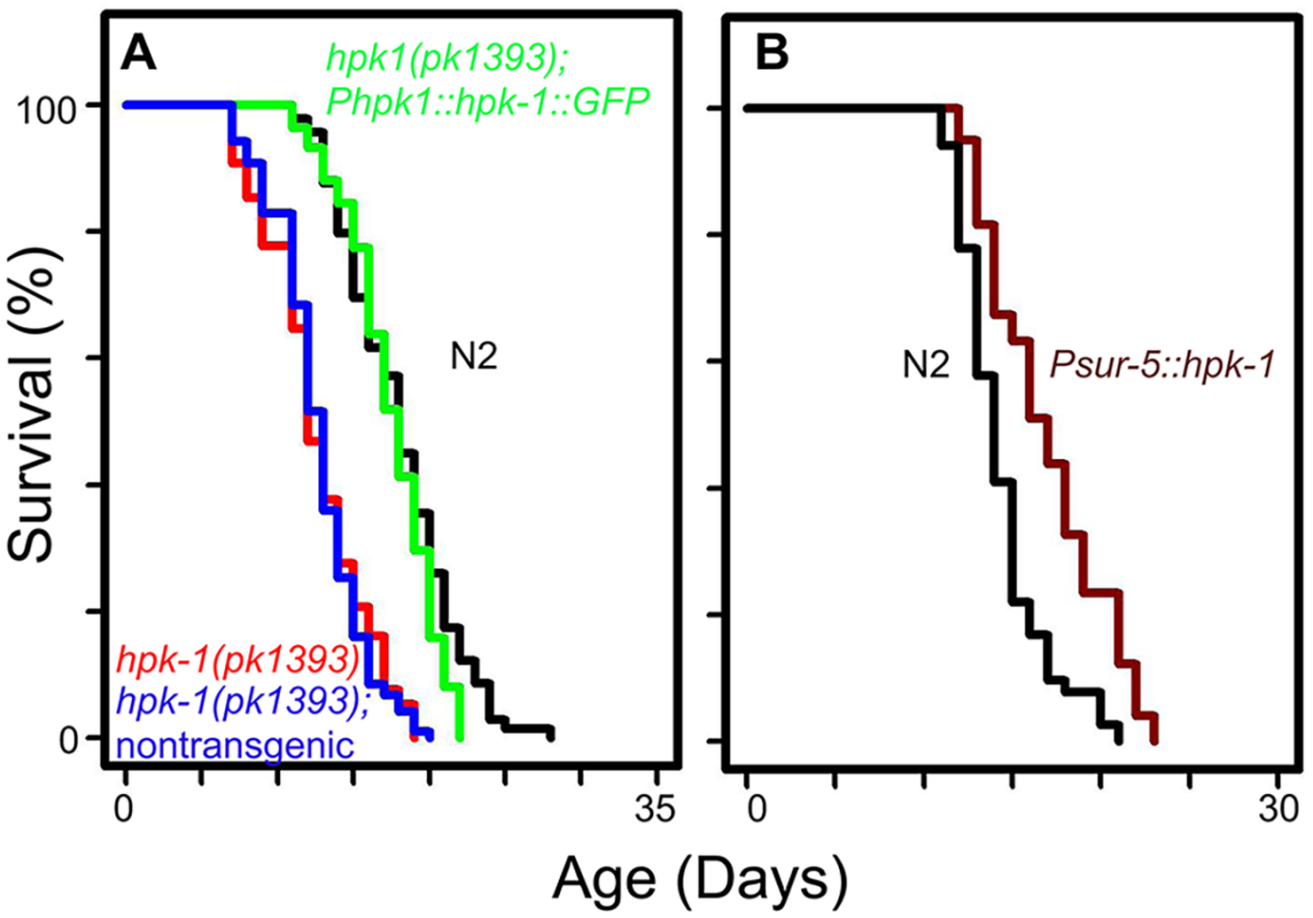
*hpk-1* activity is necessary and sufficient to regulate lifespan. (A) *hpk-1(pk1393)* null mutant animals (red) are short-lived relative to wild-type N2 (black). *Phpk-1*::*hpk-1*::*GFP* largely restores wild-type lifespan to *hpk-1(pk1393)* mutant animals (green). Lifespan of non-transgenic *hpk-1(pk1393)* siblings (blue) is similar to *hpk-1(pk1393)*. (B) Overexpression of *hpk-1* using the heterologous *sur-5* promoter increases lifespan compared to N2 (dark red versus black). Each graph is representative of at least 3 independent lifespan experiments. Full lifespan data can be found in S1 Table.

In order to verify our previous observations that *hpk-1* RNAi produces a progeric phenotype, we obtained an *hpk-1(pk1393)* deletion mutant strain that lacks most of the kinase domain and tested whether *hpk-1* was essential for normal lifespan. Loss of *hpk-1* shortened mean lifespan approximately 30% from 18–21 to 12–14 days (Fig 1A, p<0.0001, S1 Table), in line with a previous study[42]. To verify that the shortened lifespan displayed by *hpk-1(pk1393)* animals was the result of the *hpk-1* deletion and not an independent mutation, we created transgenic animals expressing an HPK-1::GFP translational fusion under control of its own promoter. Inheritance of the *Phpk-1*::*HPK-1*::*GFP* transgene rescued the progeric phenotype of *hpk-1(pk1393)*, consistent with previous reports[42]. In one trial we found that *hpk-1* overexpression with the endogenous promoter slightly increased lifespan (S1 Table), contrary to a previous study[42]. Non-transgenic *hpk-1(pk1393)* siblings remained short-lived (Fig 1A, S1 Table).

One concern when studying mutants or gene inactivations that shorten lifespan is that such genes are essential for viability and that their disruption produces a non-specific and overall “sickly” phenotype. However, overexpression of such genes would not be predicted to extend longevity unless they exert broad regulatory control over essential processes or are themselves “rate-limiting” for lifespan (like the heat shock transcription factor *hsf-1* and the *daf-16*/FOXO transcription factor). To determine if *hpk-1* is such a gene, we tested whether constitutive overexpression of *hpk-1* could increase lifespan by placing it under the control of a strong ubiquitously-expressed promoter (*Psur-5*). Overexpression of *hpk-1* (*Psur-5*::*HPK-1*::*CFP*) increased mean lifespan between 7–16% (Fig 1B, p<0.0001 and S1 Table).

### HPK-1 provides protection against polyglutamine aggregate formation and toxicity

**Fig 2:**
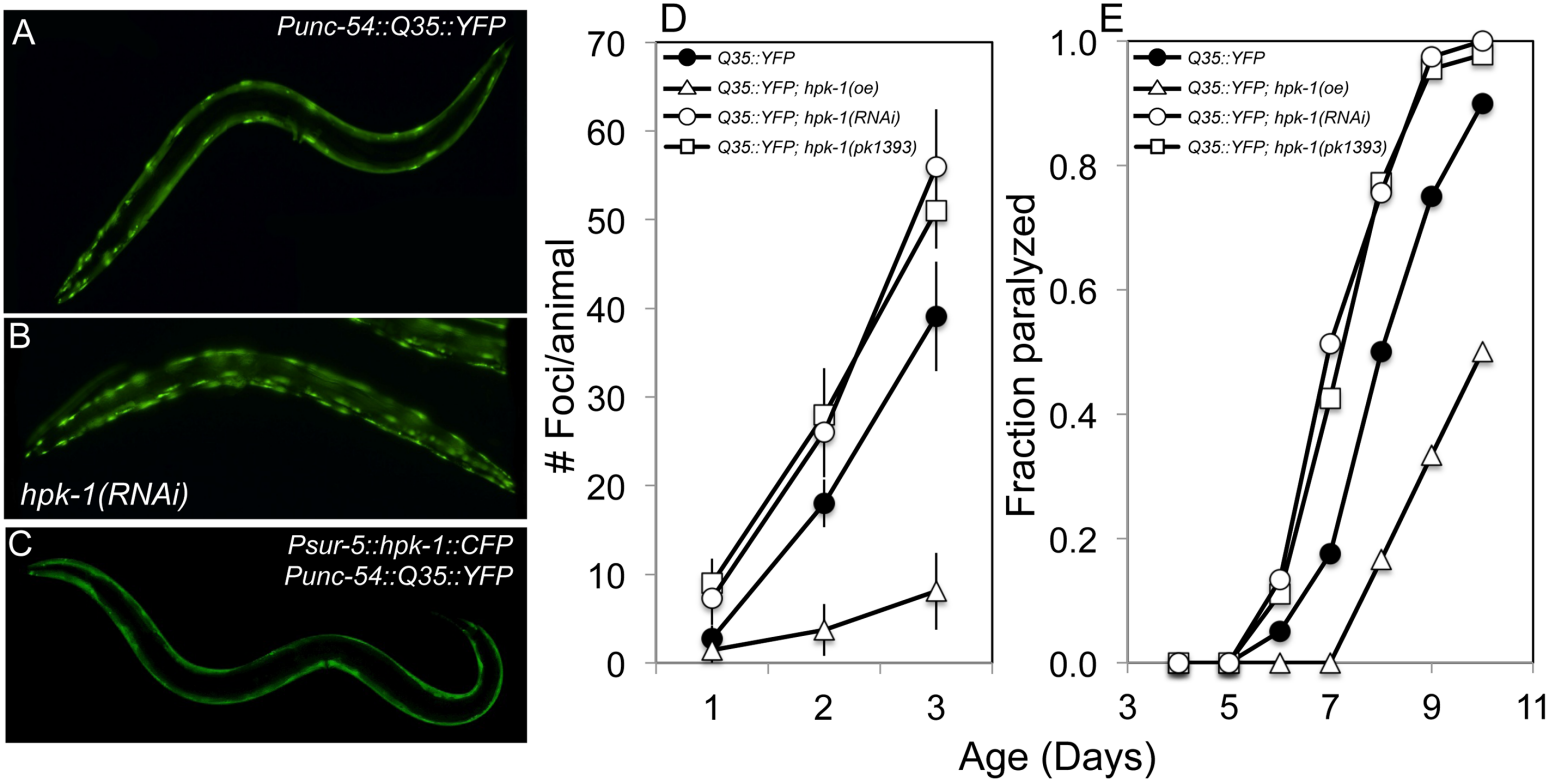
HPK-1 promotes protein homeostasis. (A-C) *hpk-1* activity affects the accumulation of Q35::YFP foci in muscle cells. Shown are representative images of *Punc-54*::*polyQ*::*YFP* animals treated with (A) control RNAi or (B) *hpk-1* RNAi, and (C) transgenic animals overexpressing *hpk-1* (*Psur-5*::*HPK-1*::*CFP*). (D) Time course of polyQ::YFP foci accumulation in conjunction with: treatment with control RNAi (black circles), *hpk-1* RNAi (white circles), *hpk-1(pk1393)* (white squares), or *hpk-1* overexpression (open triangles). Data points display the mean +/- standard deviation (S.D.) of at least 15 animals per biological replicate; at least 5 independent experiments were performed. P-values are provided in Results and in S3 Table. (E) Time course of paralysis of *Punc-54*::*polyQ*::*YFP* animals in conjunction with: treatment with control RNAi (black circles), *hpk-1* RNAi (white circles), *hpk-1(pk1393)* (white squares), or *hpk-1* overexpression (open triangles). Plotted data display the results for a single biological replicate. P-values and data from all trials are provided in S3 Table.

We next tested whether *hpk-1* plays a cytoprotective role in maintaining protein homeostasis. Age-associated decline in protein homeostasis can be measured in *C*. *elegans* through the visualization of *in vivo* polyglutamine aggregate formation in muscle cells harboring the *Punc-54*::*Q35*::*YFP* transgene, or later in life as aggregate formation overwhelms the chaperone network and locomotory paralysis ensues[43]. Loss of *hpk-1* conferred either by RNAi or the *pk1393* deletion resulted in the premature accumulation of fluorescently-labeled polyglutamine Q35::YFP aggregates (Fig 2A–2C, S3 Table p <0.0001 for both comparisons). In the representative trial displayed in Fig 2, on day 2 of adulthood, wild-type animals displayed 18.0+/-2.7 aggregates while the *hpk-1(pk1393)* null mutant and *hpk-1* RNAi-treated Q35::YFP animals averaged 28+/-5.3 and 26.0+/-5.1 aggregates, respectively (Fig 2D, S3 Table). Similarly, by day 8 of adulthood, 77–78% of *hpk-1(RNAi)* and *hpk-1(pk1393)* animals were paralyzed while 50% of control *Q35*::*YFP* animals were paralyzed (Fig 2E, S3 Table). We next tested whether overexpression of *hpk-1* was capable of conferring a cytoprotective phenotype in protein aggregation assays. Overexpression of *hpk-1* reduced both Q35::YFP foci accumulation (Fig 2D, S3 Table) and protected aging animals from *Q35*::*YFP*-associated paralysis (Fig 2E, S3 Table). Further experiments with additional transgenic lines were largely consistent with these results (S3 Table). Thus, *hpk-1* is vital for preserving protein solubility and protecting against aggregate toxicity in adult animals as they age.

### HPK-1 functions in multiple tissues and developmental stages to control longevity

In order to visualize the spatiotemporal pattern of *hpk-1* expression, we analyzed the expression of a *Phpk-1*::*hpk-1*::*GFP* transgene. *hpk-1* is expressed broadly during embryogenesis, but becomes more restricted in expression during larval development (S1A Fig). L3-stage larvae display robust expression of the GFP fusion in many head and motor neurons, and lower levels of expression in the intestine and the seam cells of the hypodermis. By late L4 stage, GFP expression is largely restricted to neurons, and is maintained in nerve cells of the head and nerve cord during adulthood, congruent with a previous study[44]. Localization of HPK-1::GFP protein is most concentrated in the nucleus often within distinct sub-nuclear sites (S1B Fig), consistent findings in mammals[45] and the predicted function of HPK-1 as a transcriptional regulator.

Identifying spatiotemporal requirements in longevity control is necessary for understanding how age-associated decline in individual tissues contributes to the larger gestalt of overall animal viability. Thus, we sought to discover where anatomically and when chronologically HPK-1 was essential for a normal lifespan. We first used stage-specific RNAi feeding to test whether the requirement of *hpk-1* for normal longevity was restricted to a particular life stage. To assess whether larval-specific activities of *hpk-1* are critical for normal adult lifespan, animals were raised on *hpk-1* RNAi during development, and were transferred to *dcr-1* RNAi at the late L4 stage in order to terminate continued silencing of *hpk-1* by RNAi. Animals raised on RNAi bacteria targeting *hpk-1* during larval development exhibited a shortened lifespan similar to lifelong inactivation of *hpk-1* (S2A and S2B Fig), while adult-restricted inactivation of *hpk-1* displayed a weaker progeric phenotype (S1C Fig). Interestingly, these temporal requirements are essentially identical to those previously described for *hsf-1*[46]. Given the broad developmental expression of *hpk-1*, we next sought to define the tissues where *hpk-1* acts to promote normal longevity. Tissue-restricted RNAi of *hpk-1* in the intestine or the hypodermis both caused a significant progeric phenotype (S2D and S2E Fig), consistent with intestinal and hypodermal expression being limited to larval developmental stages. In contrast, inactivation of *hpk-1* in muscle cells had little effect on lifespan (S2F Fig), consistent with the absence of HPK-1 expression in these cells as determined with our fluorescent reporter (S1A Fig, and S1 File).

We next tested whether neuronal *hpk-1* function was necessary for normal lifespan using an enhanced neuronal RNAi (*RNAi(en)*) strain, as RNAi efficiency in neurons is low in wild-type animals. Neuronal inactivation of *hpk-1* showed reduced lifespan to an extent comparable to inactivation of *hpk-1* by systemic RNAi (S2G Fig) and *hpk-1* null mutant animals (Fig 1A). As a positive control to confirm *RNAi(en)* activity, *daf-2(RNAi)* significantly increased lifespan in the *RNAi(en)* strain (S2H Fig) while a control strain lacking the dsRNA channel *sid-1* and enhanced neuronal RNAi did not (S2I Fig), consistent with previous reports[47, 48]. Thus *hpk-1* is required across all of the tissues in which we have observed its expression during the larval stages of development to ensure wild-type lifespan. Because HPK-1::GFP expression is restricted to neurons in adult animals (S1A Fig), we interpret the longevity-extending activity of HPK-1 observed in the intestine and hypodermal seam cells (S2D and S2E Fig) to arise largely from larval-stage functions of HPK-1 in those tissues, although *hpk-1* does have modest longevity-extending effects in adulthood as well (S2C Fig).

### HPK-1 is a member of the HSF-1 longevity pathway

**Fig 3:**
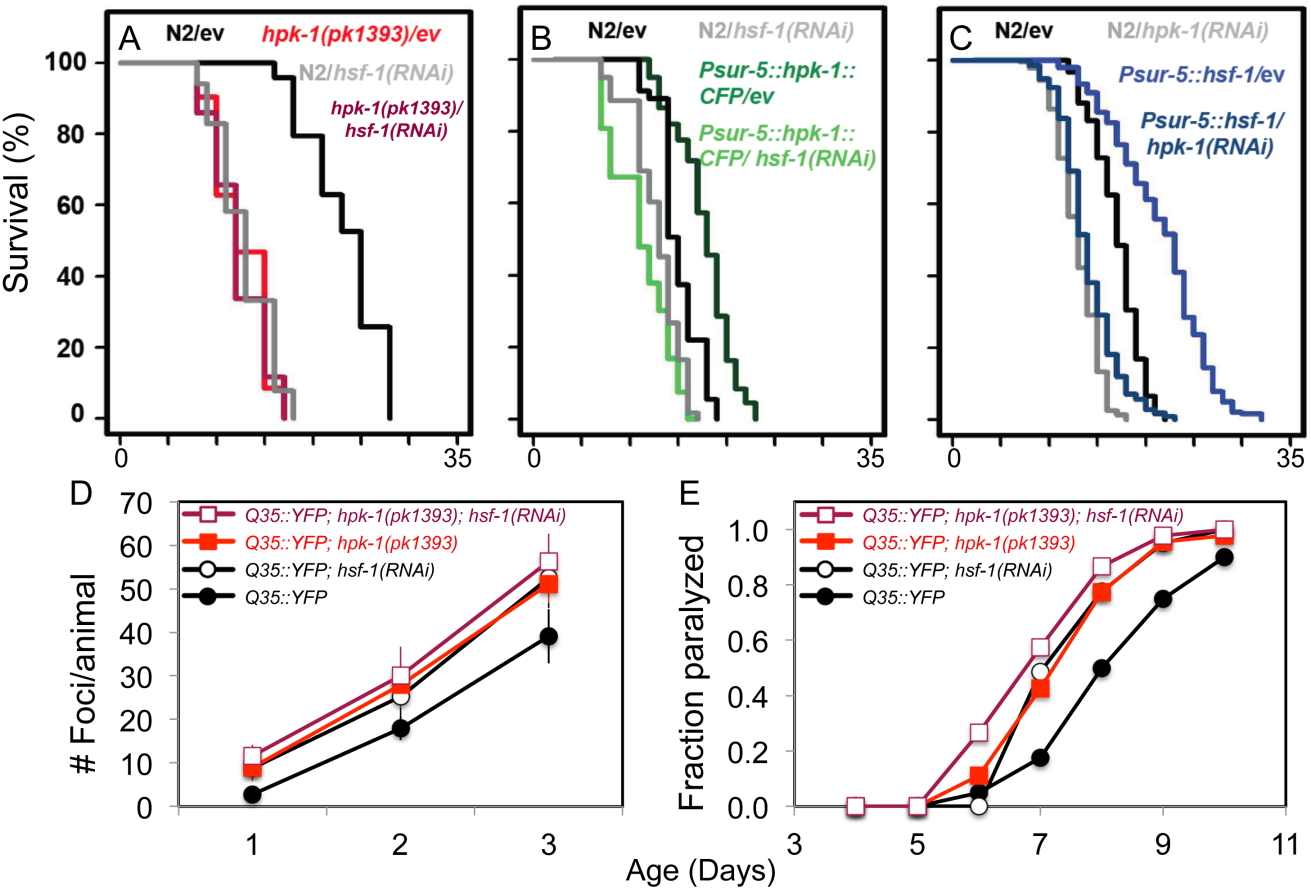
*hpk-1* and *hsf-1* have overlapping functions in longevity control and the preservation of proteostasis. (A) *hpk-1(pk1393)* null mutant animals are short-lived compared to wild-type controls (red versus black) and *hsf-1(RNAi)* does not further shorten *hpk-1(pk1393)* lifespan (maroon versus gray). (B) Overexpression of *hpk-1* increases lifespan (dark green versus black) and is *hsf-1* dependent (light green). (C) Constitutive *hsf-1* overexpression increases lifespan (blue versus black) and is *hpk-1* dependent (light blue). Each graph is representative of at least 3 independent lifespan experiments. Full lifespan data can be found in S1 Table. (D-E) Simultaneous loss of *hpk-1* and *hsf-1* does not produce additive detrimental effects on protein homeostasis (maroon versus red). (D) Time course of *Punc-54*::*Q35*::*YFP* foci accumulation in conjunction with: treatment with control RNAi (filled circles/squares), *hsf-1* RNAi (white circles/squares) in either wild-type (black traces) or the *hpk-1(pk1393)* null mutant background (colored traces). Data are the mean +/- S.D. of at least 15 animals from one representative trial; at least 5 independent experiments were performed. P-values are provided in Results and S3 Table. (E) Time course of paralysis of *Punc-54*::*polyQ*::*YFP* animals after treatment with control RNAi (filled circles/squares) or *hsf-1* RNAi (white circles/squares) in either wild-type (black traces) or the *hpk-1(pk1393)* null mutant background (colored traces). Data is representative of one biological replicate, with at least 5 independent replicates performed. P-values and data from all trials are provided in S3 Table.

Based on a pilot screen to identify putative genetic interactions between known longevity genes and *hsf-1* loss of function, we investigated the extent to which *hpk-1* exerts its effects on overall longevity and proteostasis via *hsf-1*. We measured the extent to which *hpk-1* exerts its effects on overall longevity and proteostasis via the HSF-1 pathway by examining whether their loss-of-function phenotypes were additive. We observed that *hsf-1* inactivation in *hpk-1(pk1393)* null mutant animals did not result in a meaningful additional decrease in lifespan (Fig 3A). Further, inactivation of *hsf-1* by RNAi was sufficient to suppress the increased lifespan of the long-lived *hpk-1* overexpression line (Fig 3B). Conversely, *hpk-1* was necessary for the extended longevity observed in *hsf-1-*overexpressing animals (Fig 3C). The reciprocal requirement we observed between *hpk-1* and *hsf-1* for animal longevity is evidence of a genetic interaction between these factors. Next we tested whether *hpk-1* and *hsf-1* function together or in separable pathways to protect animals from Q35::YFP foci formation by comparing animals lacking both *hpk-1* and *hsf-1* to animals deficient in only one of these genes. As expected, loss of *hpk-1* or *hsf-1* alone resulted in the premature accumulation of protein aggregates and onset of paralysis (Fig 3D and 3E). Inactivation of *hsf-1* by RNAi in the absence of *hpk-1* failed to produce a statistically detectable increase in the accumulation of foci or onset of paralysis over time (Fig 3D and 3E). Additional experiments corroborated these results: *hpk-1* RNAi-treatment alone had as great or a greater negative impact on proteostasis when compared to *hsf-1* RNAi treatment (S3 Table). These results are consistent with the notion that *hpk-1* and *hsf-1* function to maintain protein homeostasis and delay the progression of aging through a shared mechanism.

**Fig 4:**
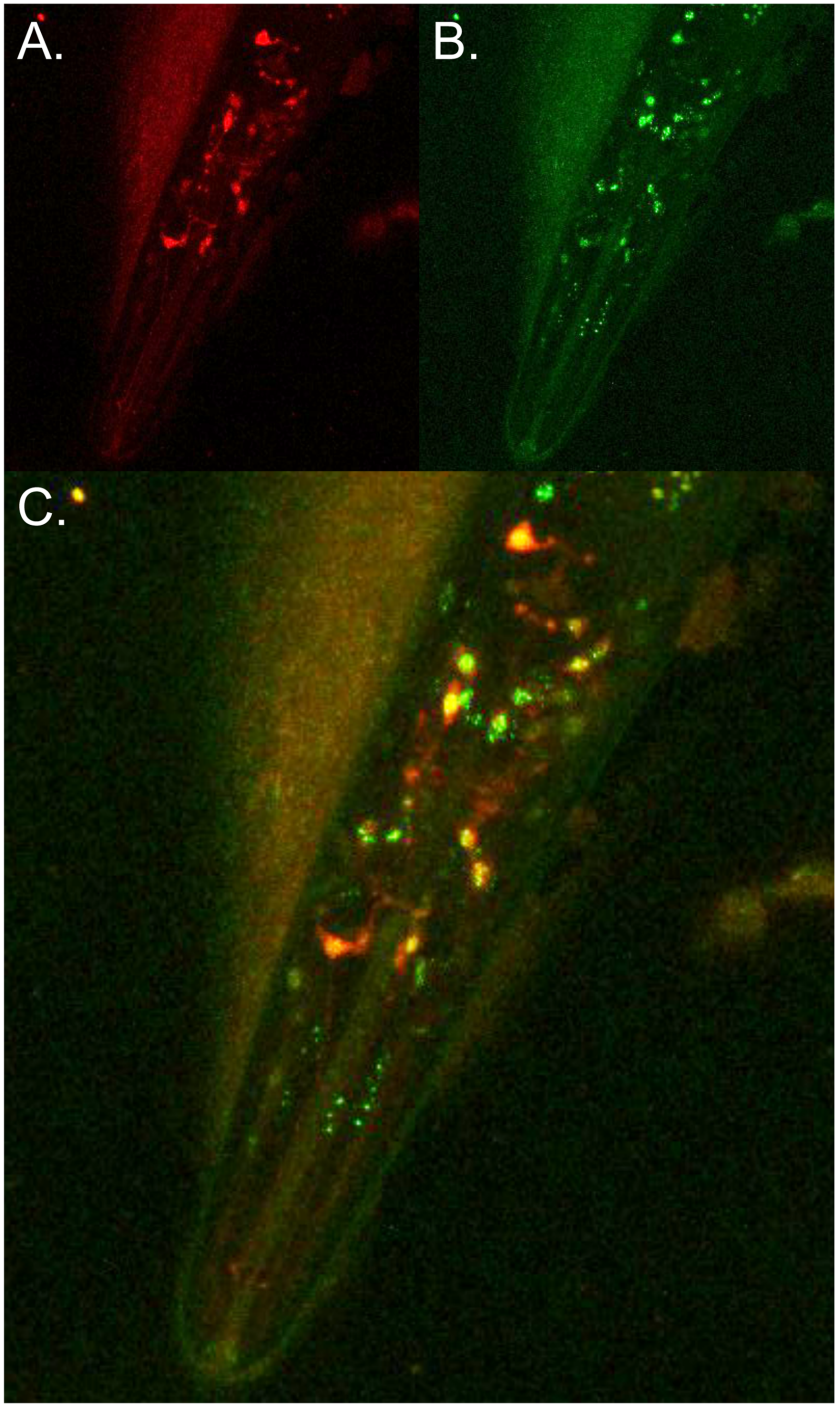
HPK-1 colocalizes with HSF-1 in *C*. *elegans* neurons. (A-C) HPK-1 and HSF-1 colocalize in neurons under basal conditions. Representative image of transgenic animal co-expressing *Phpk-1*::*HPK-1*::*tdtomato*,*Phsf-1*::*HSF-1*::*GFP*: (A) red fluorescence, (B) green fluorescence, and (C) overlay.

That homeodomain interacting protein kinases function as direct regulators of transcription factor activity suggested that the interaction between HPK-1 and HSF-1 may be direct. To begin to explore this possibility, we examined whether HPK-1 co-localizes with HSF-1 at the subcellular level by comparing localization of a *Phsf-1*::*hsf-1*::*GFP* transgene to a translational fusion between *hpk-1* and the fluorescent *tdtomato* protein (*Phpk-1*::*hpk-1*::*tdtomato*) using confocal microscopy (Fig 4). Though not perfectly overlapping, HPK-1 and HSF-1 localization were often coincident with each other.

**Fig 5:**
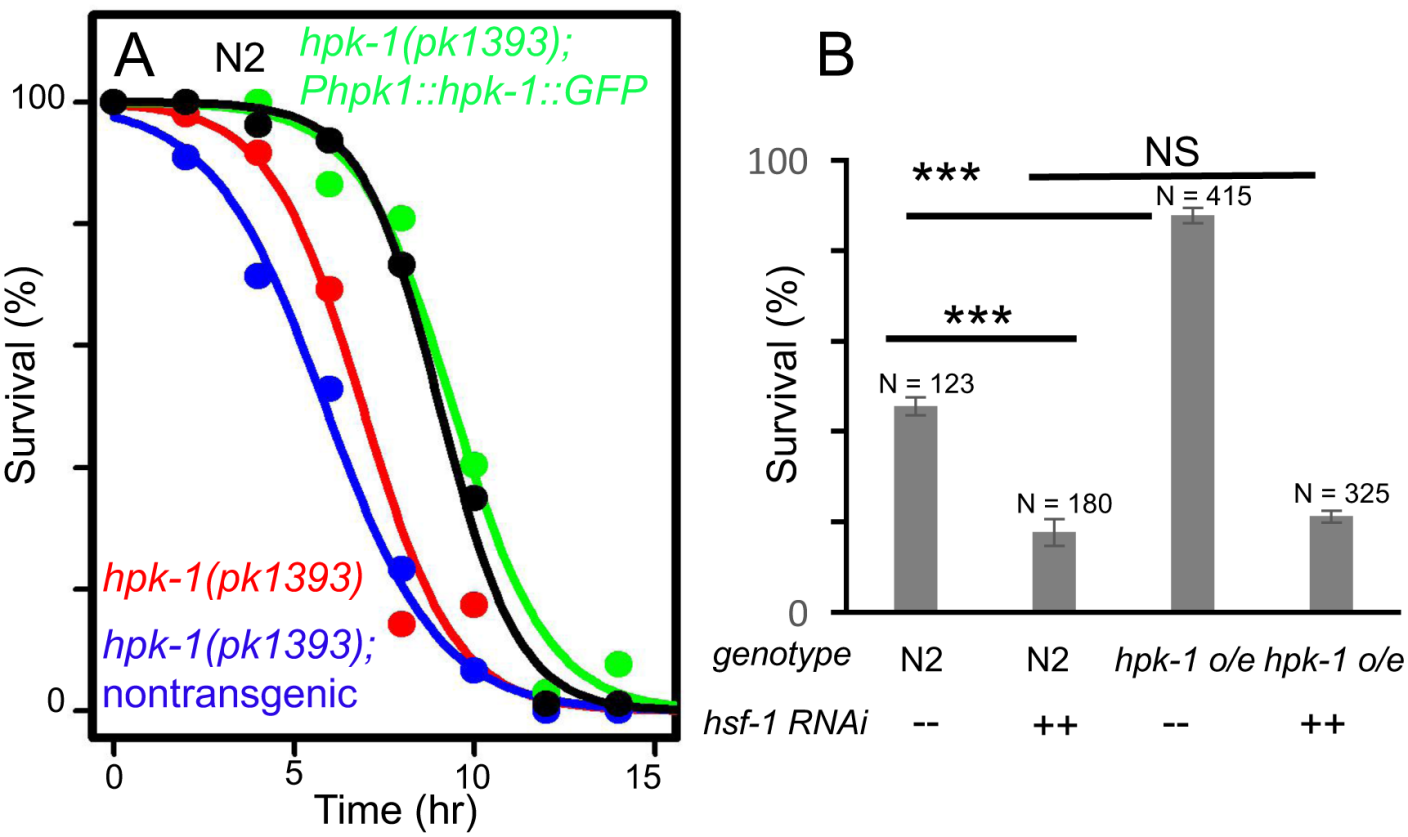
*hpk-1* regulates thermotolerance. (A) *hpk-1* is required for normal thermotolerance, as *hpk-1(pk1393)* has impaired survival at 35°C (red vs. black), which is rescued by transgenic *Phpk-1*::*hpk-1*::*GFP* (green). Graph represents survival data from one representative trial of three independent replica set experiments. Additional trials can be found in S2 Table; see methods and [38, 72] for additional details and statistical analysis. (B) *hpk-1* overexpression increases survival to thermal stress (column 3 versus 1), dependent on *hsf-1* (column 4 versus 2). Plotted data show mean and S.E.M. of a representative trial (trial 1) with 3–4 technical replicates per condition; # above each column represents total number of animals scored per condition. See S2 Table for additional details. *** indicates p-values of <0.001. P-values were calculated using ANOVA with Tukey’s HSD post-hoc, and were corrected to account for multiple testing. See S2 Table for additional trial data.

We next sought to determine if *hpk-1* shares functionality with HSF-1 with respect to the heat shock stress response by examining its role in regulating the transcriptional induction of chaperone gene expression after exposure to heat. We initially tested whether *hpk-1* is important for preserving thermotolerance in response to heat shock. We observed that survival at 35°C was significantly reduced in *hpk-1(pk1393)* mutant animals, and that thermotolerance could be restored to wild type levels by the rescuing *hpk-1* transgene (*Phpk-1*::*hpk-1*::*GFP*) (Fig 5A). Furthermore, we observed that the long-lived *hpk-1* overexpression line (*Psur-5*::*hpk-1*::CFP) increased thermotolerance survival from ~50% in wild type animals to ~75%, and that this increase in thermotolerance was fully dependent on *hsf-1* (Fig 5B).

### HPK-1 prevents sumoylation of HSF-1

**Fig 6:**
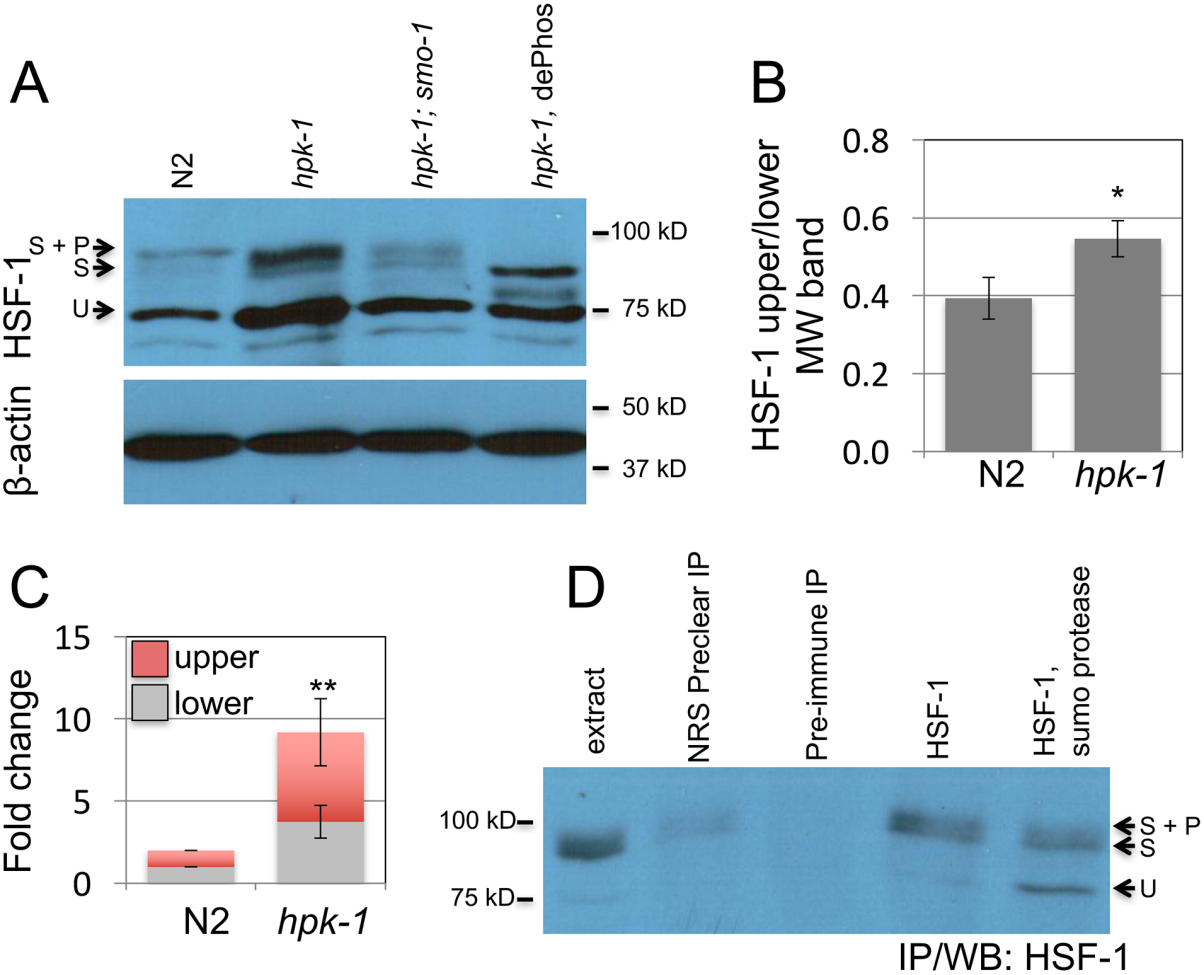
*hpk-1* prevents sumoylation of HSF-1. (A) Changes in HSF-1 post-translational modifications between early L4 wild-type and *hpk-1(pk1393)* animals were examined by western blot to HSF-1; *smo-1(RNAi)*, which targets *C*. *elegans* SUMO, was used to block sumoylation, dePhos is lambda protein phosphatase treatment (other samples were mock treated). Beta-actin serves as a loading control. The ratio of modified to unmodified HSF-1 is 0.35, 0.51, and 0.35 for N2/*ev*, *hpk-1(pk1393)/ev*, and *hpk-1(pk1393)/smo-1(RNAi)*, respectively (see S3A Fig for additional data). (B) *hpk-1* prevents sumoylation of HSF-1. Ratio of HSF-1 unmodified (75kD) to modified (90-95kD, sumoylated and sumoylated plus phosphorylated). The S.E.M. from Image J quantification is shown for seven N2 and *hpk-1(pk1393)* replicates (* p<0.02, Student’s t-test, see S3B and S3C Fig for additional data). (C) Loss of *hpk-1* induces HSF-1 in L4 animals. Protein levels of unmodified (75kD, grey), modified (90-95kD, red) HSF-1, and beta-actin were quantified in ImageJ for four replicates (see S3C Fig for additional data). (For total, upper, and lower MW fold change (respectively): ** p<0.01, <0.01, and <0.02, Student’s t-test). (D) Immunoprecipitation of HSF-1 from *hpk-1(pk1393)* animals followed by SUMO protease or mock treatment. First lane is protein extract prior to immunoprecipitation. Lysate was precleared of nonspecific interactions by treatment with normal rabbit serum (Invitrogen #01–6101) that was then immunoprecipitated (lane 2). Precleared lysate was then evenly divided and treated with either preimmunized rabbit serum (i.e. serum from the rabbit in which *C*. *elegans* HSF-1 antibodies were generated, prior to immunization, lane 3) or HSF-1 antiserum, followed by immunoprecipitation. Immunoprecipitated HSF-1 was then either mock treated or treated with SUMO protease (lanes 4 and 5). See methods for additional details.

HSF-1 transcriptional activity is regulated in mammals through a complex array of post-translational modifications including phosphorylation, acetylation, and sumoylation (reviewed in[41, 49]). We sought to determine whether loss of *hpk-1* altered either expression levels and/or post-translational modifications to the HSF-1 protein. Unmodified HSF-1 displayed the predicted mobility of a ~75 kD protein[50]. We additionally observed in wild-type animals two higher molecular weight isoforms between ~90 and 95 kD (Figs 6A, S3). Loss of *hpk-1* resulted in an increase in the ratio of higher molecular weight isoforms of HSF-1 to the unmodified 75 kD species (Figs 6B, S3B) and an increase in overall levels of HSF-1 protein relative to the β-actin control (Figs 6A and 6C, S3C).

Sumoylation typically results in an electrophoretic mobility upshift of ~15 kD[51]. Mammalian HSF-1 has been shown to be sumoylated[40]. Consequently, we hypothesized that the two higher MW isoforms of HSF-1 might represent a sumoylated product (S isoform) (Figs 6A and S3, lower ~90 kD MW band), and SUMO plus phosphorylation (S+P isoform) (Figs 6A and S3, upper ~95 kD MW band). Consistent with this hypothesis, lambda protein phosphatase treatment of *hpk-1* null extracts resulted in the loss of the highest MW isoform of HSF-1 but not the ~90 kD isoform (Figs 6A, S3A), suggesting that the 90 kD isoform is not a result of phosphorylation events. This result confirms the hypothesis that HSF-1 phosphorylation is a modification of the 90 kD isoform. However, the 95 kD band is still observed in an *hpk-1* mutant, indicating that HPK-1 is not the kinase responsible for this phosphorylation event. We determined that the higher molecular weight isoforms likely represent sumoylated forms of HSF-1 by two approaches. First, *hpk-1* null mutant animals were grown on *smo-1* RNAi, which reduces expression of the *C*. *elegans* SUMO gene that produces the SUMO moiety. *smo-1* RNAi of *hpk-1* mutant animals resulted in a decrease in the ratio of the pair of high MW bands to unmodified HSF-1 at 75 kD. Second, HSF-1 was immunoprecipitated and treated with SUMO protease, which resulted in a relative increase in the 75 kD (unmodified) HSF-1 isoform and a relative decrease in the 90–95 kD (sumoylated) bands of HSF-1 (Fig 6D). While the loss of the sumoylated species was incomplete, each result is consistent with our prediction that the higher molecular weight isoforms of HSF-1 are the result of sumoylation. Thus, HPK-1 acts directly or indirectly to oppose HSF-1 sumoylation, either by blocking sumoylation or promoting de-sumoylation.

**Fig 7:**
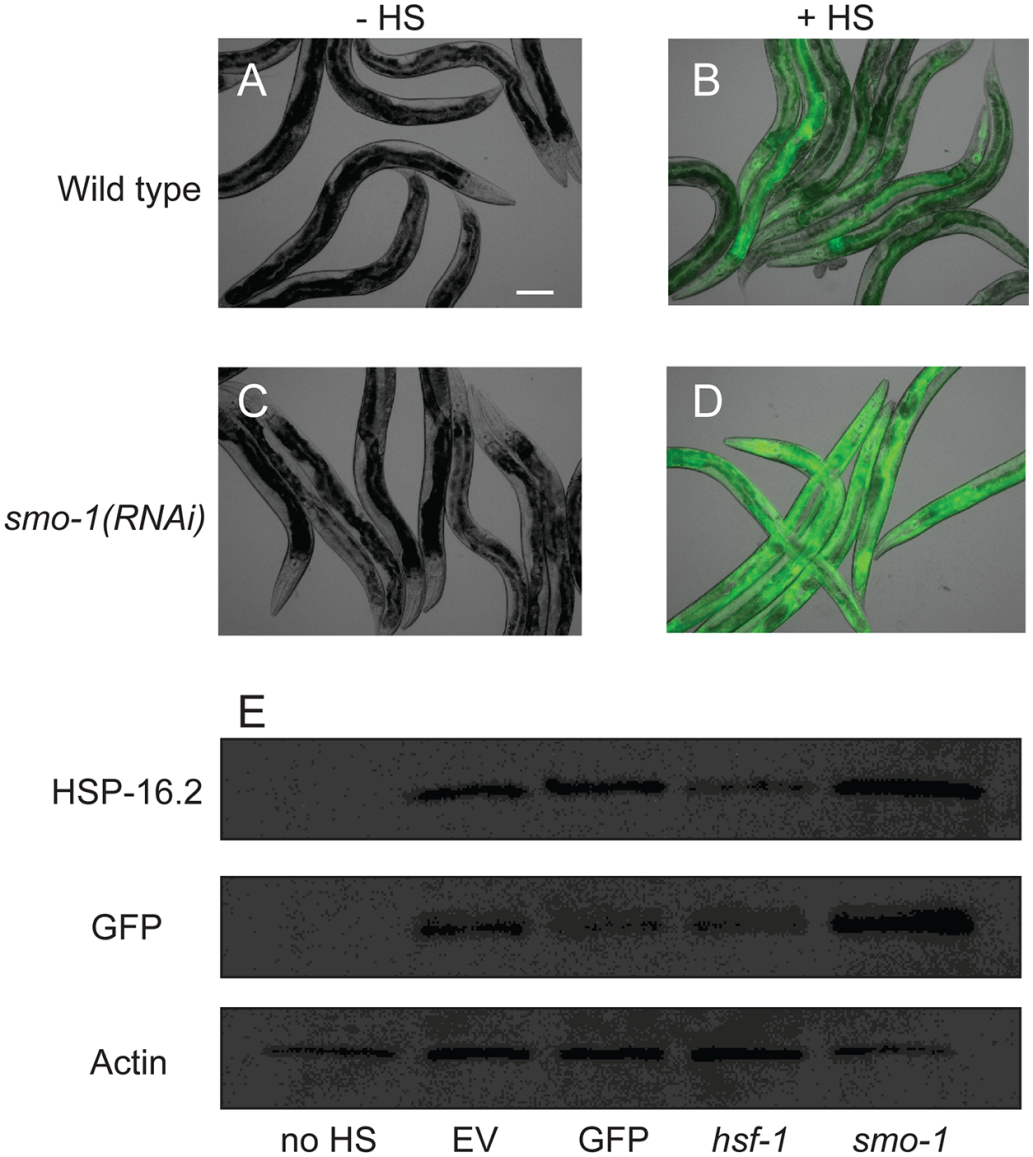
Heat shock induction of *hsp-16*.*2* is enhanced by *smo-1(RNAi)*. (A-D) DIC and GFP overlay for *Phsp-16*.*2*::*GFP* worms on empty vector (A, B) or *smo-1(RNAi*) (C, D) with (+HS) and without (-HS) heat shock. Scale bar = 100μm. (E) Western blot for HSP-16.2, GFP and β-actin from *hsp-16*.*2p*::*GFP* worms grown on empty vector (EV) without heat shock (no HS) or with heat shock (EV), *GFP(RNAi)*, *hsf-1(RNAi)* or *smo-1(RNAi)*. Fold-increases for HSP-16.2/actin on *smo-1(RNAi)* relative to EV in three independent replicates were 2.4, 4.7, and 1.8.

We then tested our hypothesis that sumoylation is an inhibitory modification on HSF-1 by analyzing induction of the HSF-1 transcriptional program in response to heat shock in animals grown on *smo-1* RNAi (Fig 7). In response to thermal stress, *smo-1* RNAi-treated animals harboring the *Phsp-16*.*2*::*GFP* transgene displayed enhanced induction of GFP relative to empty vector control animals (Fig 7A–7D). It is worth noting that in the absence of heat shock, the *Phsp-16*.*2*::*GFP* is not induced, indicating that loss of sumoylation is not sufficient of itself to activate the HSF-1 transcriptional response. In a complementary experiment, we analyzed protein induction of GFP expressed from the *hsp-16*.*2* promoter as well as the endogenous HSP-16.2 protein in response to heat shock (Fig 7E). Prior to heat shock, animals were raised on RNAi targeting *GFP*, *hsf-1*, *smo-1* or the empty vector control. In response to heat shock, both GFP and HSP-16.2 were induced. GFP but not HSP-16.2 induction was blocked by *GFP(RNAi)*. Both GFP and HSP-16.2 induction were significantly reduced by *hsf-1(RNAi)*, confirming that induction of these proteins was dependent on the presence of HSF-1. Finally, *smo-1(RNAi)* increased the level of GFP and HSP-16.2 protein when compared to the EV control, supporting our hypothesis that HSF-1 sumoylation is a modification that is likely to inhibit the HSF-1 transcriptional program in response to heat shock.

### Hormetic extension of natural longevity requires both *hsf-1* and *hpk-1*

In general, the sustained hardship of chronic exposure to an environmental stressor, or application of a stressor to a level above a physiologically tolerable threshold typically compromises organism viability (and consequently limits organismal lifespan). Hormesis is the initially counterintuitive but now well-validated and generalizable phenomenon produced by acute exposure to an environmental stressor. Brief intervals of exposure to stressors (e.g. heat, ROS, etc.) can confer a “hormetic effect” characterized by a significant extension of longevity accompanied by sustained resistance to physiologic stress. For instance, a transient 30°C pulse during development and/or early adulthood increases lifespan in *C*. *elegans*, while sustained growth at 25°C hastens the onset of animal morbidity[52]. It is generally postulated that the mechanism of extended longevity following heat shock is derived from upregulation of chaperone systems and turnover of misfolded proteins, which consequently promotes long term proteostatic robustness/resiliency once heat stress is removed[53]. HSF-1 is necessary for the hormetic effect of transient heat exposure on lifespan[54].

We tested whether *hpk-1* was required for heat-induced hormetic extension of longevity. While defining experimental conditions for heat-induced hormesis, we found that growth at 25°C for the first 3 days of adulthood is a form of mild heat stress that to our knowledge has not been previously documented in *C*. *elegans* (S4A–S4F Fig). We subsequently observed that transient growth at 25°C during this interval in early adulthood (when heat-resistance mechanisms at 20°C normally undergo a dramatic downregulation[29, 30, 55]) was sufficient to increase lifespan only in the presence of *hsf-1* and *hpk-1* (S4G and S4H Fig, p<0.001). Thus *hpk-1*, like *hsf-1*, is necessary for hormetic extension of longevity in response to heat stress, a result consistent with a positive regulatory function of HPK-1 over HSF-1.

### HPK-1 is an essential component of the heat shock response

*hipk* family members canonically function as positive regulators of transcriptional co-activators. If the interaction between HPK-1 and HSF-1 is direct, *hpk-1* could be promoting HSF-1 activity at various regulatory points in the chain of events beginning with newly translated HPK-1 and ending with induction of gene transcription by HSF-1. For example, post-translational modification of HSF-1 by HPK-1 could affect its stability, subcellular localization, DNA binding or transactivation activity at the level of recruitment of RNApol II, additional transcription factors, or chromatin modifying factors. We undertook several experiments to identify mechanisms of regulatory control.

Immediately following thermal stress, acute cytoplasmic misfolding challenges the chaperone network to free HSF-1 from Hsp90 cytoplasmic sequestration, allowing HSF-1 to undergo translocation to the nucleus, where it becomes concentrated in stress granule-like subnuclear speckles that colocalize with markers of active transcription[56]. Re-allocation and compartmentalization of HSF-1 to nuclear speckles occurs within minutes of initiating thermal stress ([57] and this study). When we tested whether *hpk-1* was necessary for HSF-1 re-localization or compartmentalization in response to heat shock, we found that these early events of HSF-1 activation are *hpk-1* independent, as *hpk-1(RNAi)* had no effect on either readout of HSF-1 activation (S5 Fig). Therefore *hpk-1* is likely to play a role in subsequent events during activation and/or establishment of the HSF-1 transcriptional response.

**Fig 8:**
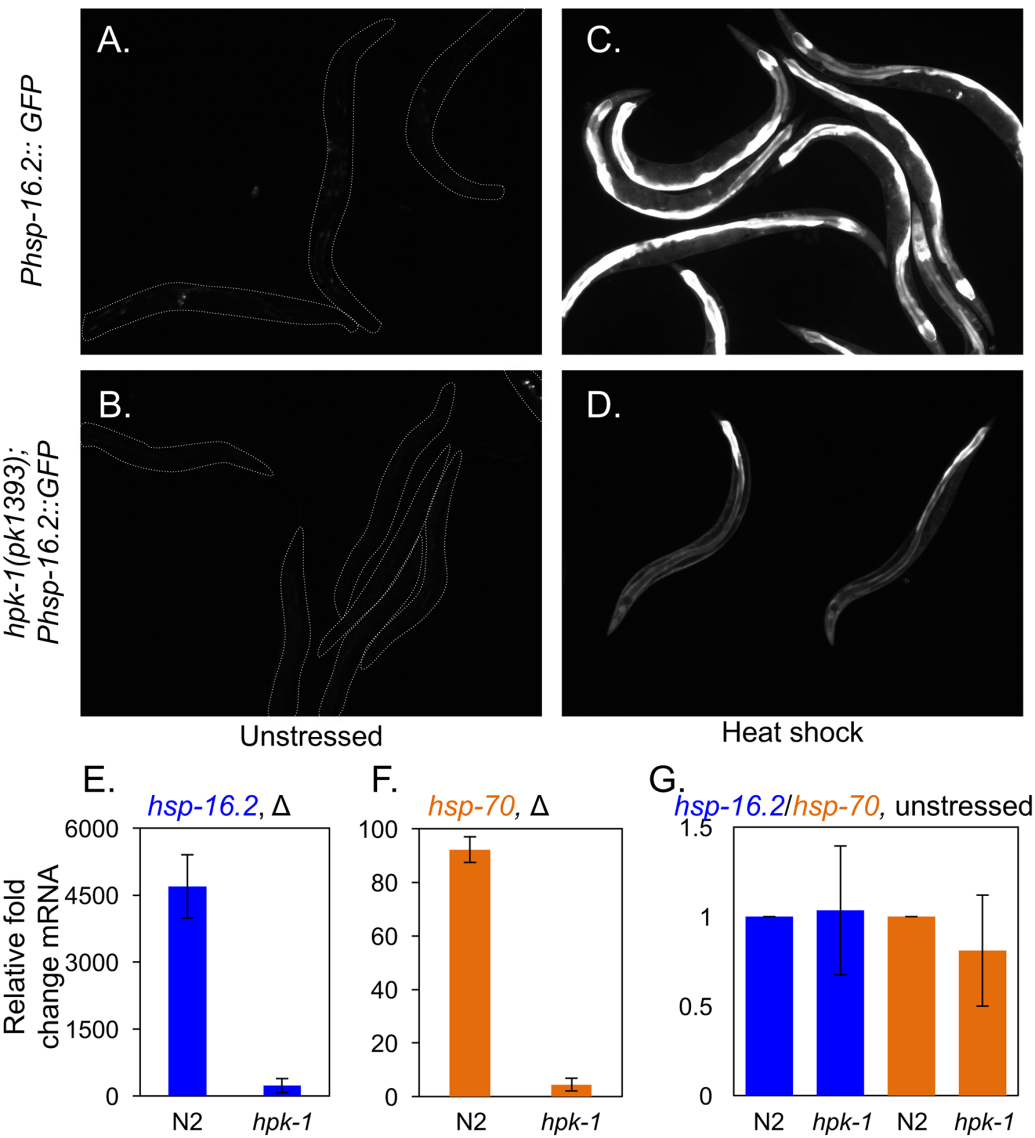
*hpk-1* regulates induction of the heat shock response. (A-D) Representative images of at least 30 *Phsp-16*.*2*::*GFP* animals in wild type (A, C) and *hpk-1(pk1393)* animals (B, D) under basal conditions (A, B) or after heat shock (C, D). Animals are outlined in panels A-B. (E-F) Induction of endogenous *hsp-16*.*2* (E) and *hsp-70* (C12C8.1) (F), in N2 and *hpk-1(pk1393)* animals as measured by qRT-PCR. (G) Loss of *hpk-1* did not alter endogenous expression of *hsp-16*.*2* (blue) or *hsp-70* (orange). Values in (E-G) are normalized to expression of *act-1* and the mean fold change relative to wild-type animals, and the S.E.M. between technical replicates is shown. In total three independent experiments were performed with similar results. P-values for (E) and (F) are <0.05 and <0.01, respectively (Student’s t-test).

We tested whether *hpk-1* was necessary for the transactivation of HSF-1 by determining whether the induction of chaperone target genes in response to thermal stress was compromised in the absence of *hpk-1*. We first analyzed whether *hpk-1* was required for induction of the *Phsp-16*.*2*::*GFP* reporter. *hsp-16* encodes a small chaperone that is induced by heat shock in a manner requiring *hsf-1*[58]. We found that *hpk-1* was also necessary for *hsp-16-2*::*GFP* induction in response to transient heat shock (Fig 8A–8D) as previously shown[42]. In addition, we found that *hsf-1*-dependent transcriptional induction of the endogenous chaperones encoded by *hsp-16*.*2* and *hsp-70* also required the presence of *hpk-1* for heat shock inducibility (Fig 8E and 8F), which unexpectedly differs from a previous report[42]. HPK-1 regulation of chaperone gene expression is dependent on heat stress, as loss of *hpk-1* did not significantly alter basal expression levels of *hsp-16*.*2* and *hsp-70* (Fig 8G). In contrast, *hsf-1* inactivation has been reported to reduce endogenous levels of *hsp-16*.*2* and *hsp-70* mRNA by ~40%[50], suggesting some basal HSF-1 activity remains in the absence of *hpk-1*.

**Fig 9:**
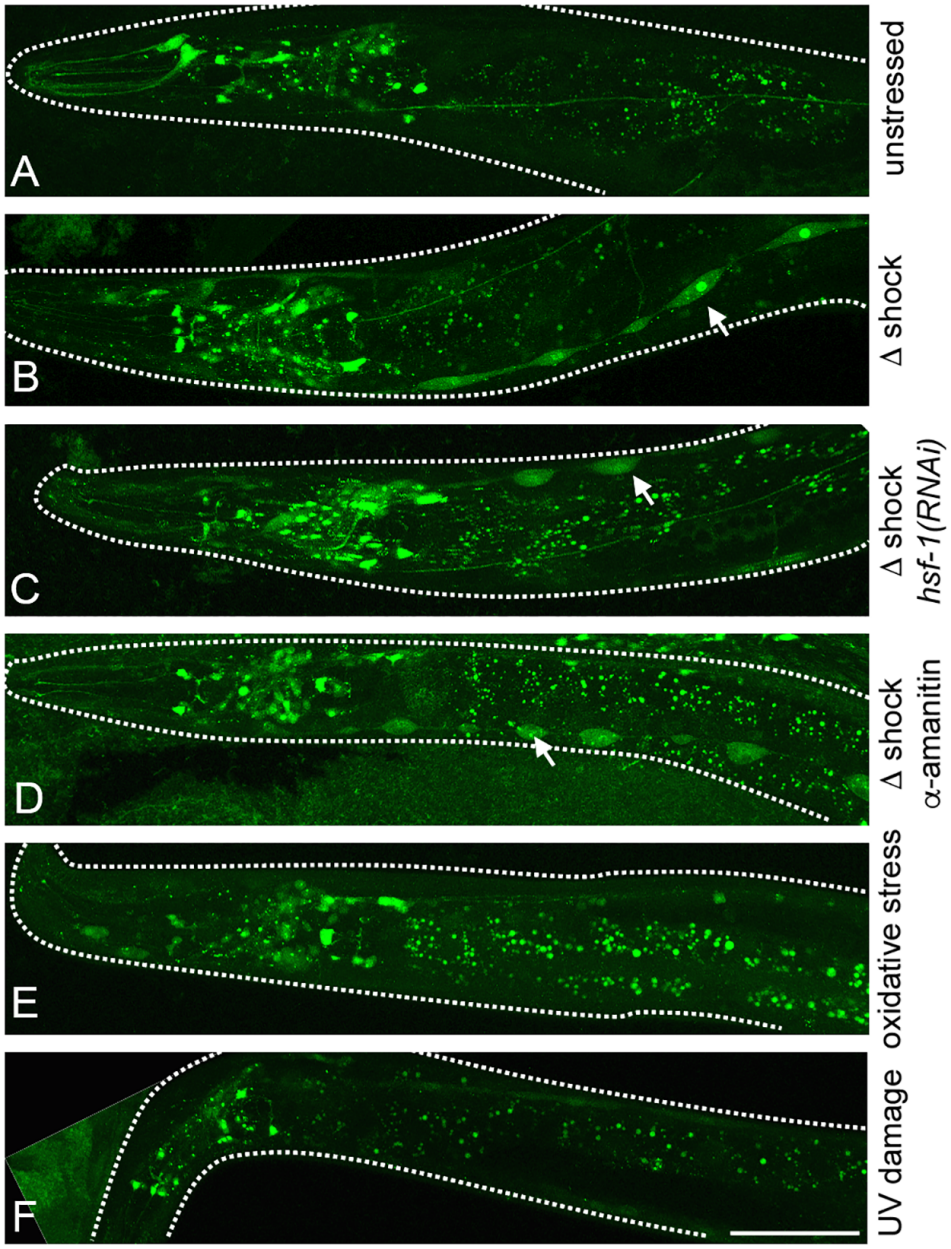
*hpk-1* protein expression is stimulated by heat shock. (A) Expression of HPK-1 protein (*Phpk-1*::*HPK-1*::*GFP*) under basal conditions is primarily restricted to neurons. Fluorescent intestinal speckles are non-specific gut granules[111]. (B-D) Heat shock induces HPK-1 protein (*Phpk-1*::*HPK-1*::*GFP*) levels most strongly within hypodermal seam cells (indicated by arrows) independent of *hsf-1* (C) and transcription (D). *Phpk-1*::*HPK-1*::*GFP* animals after heat shock with (B) empty vector HT115, (C) *hsf-1(RNAi)*, and (D) α-amanitin treatment. Increased HPK-1 expression within neurons and hypodermal seam cells is specific to heat stress as neither oxidative stress (E) or UV damage (F) altered expression. White space was artificially filled for some images and animals are outlined. GFP quantification and further analysis can be found in S6 Fig.

We also considered the possibility that HSF-1 functions upstream rather than downstream of HPK-1, or as part of a feedback loop with HSF-1. We examined HPK-1 expression in response to multiple conditions of stress including heat shock, and tested whether HPK-1 expression is regulated by HSF-1. Under basal conditions, transgenic animals that express a GFP translational fusion that includes the *hpk-1* open reading frame (*Phpk-1*::*hpk-1*::*GFP*) displayed a broad pattern of developmental expression in the intestine, hypodermal seam cells, and neurons. The expression pattern of this transgene became restricted to neurons as animals transitioned to adulthood (Figs 9A, S1A). We next tested whether the pattern of *hpk-1* expression is regulated by thermal stress. We observed robust induction of *hpk-1* expression in transgenic animals expressing the translational reporter after heat shock (Fig 9B). Induction of HPK-1 was greatest in hypodermal seam cells, neurons, and to a much lesser extent within intestinal cells (Figs 9B, S5), and this induction did not require *hsf-1* (Fig 9C).

We asked whether HPK-1 induction was transcriptional or post-transcriptional, as early events in many stress response pathways including the heat shock response do not require active transcription. Consistently, we could not discern an increase in fluorescence in transgenic animals expressing a transcriptional fusion of the *hpk-1* promoter to GFP (*Phpk-1*::*GFP*) (S7A and S7B Fig). However, we did notice that hypodermal seam cells appeared much larger and swollen compared to unstressed controls (S7A and S7B Fig, S1 File). As extrachromosomal transgenic reporter lines lack both normal gene copy number and the context of endogenous chromatin, we further examined *hpk-1* mRNA levels in wild-type animals by qRT-PCR but found no significant difference in mRNA expression as a function of heat shock (S7C Fig). Importantly, endogenous *hsp-16*.*2* mRNA was induced in the heat stressed sample (S7D Fig). Because *hsf-1* is a global activator of chaperones and other heat shock response genes, we asked whether the thermal inducibility of HPK-1 translation requires *hsf-1*. Consistent with a mode of post-transcriptional regulation of *hpk-1* in response to heat shock, *hsf-1(RNAi)* had no effect on the induction of the translational *Phpk-1*::*hpk-1*::*GFP* reporter after heat shock (Fig 9C) and induction was not blocked by pre-treatment with the RNA polymerase inhibitor α-amanitin (Fig 9D). In contrast, α-amanitin pre-treatment completely blocked the induction of the known transcriptional *Phsp-16*.*2*::*GFP* reporter in response to heat shock (S8 Fig). Induction of HPK-1 protein is specific to thermal stress, as oxidative damage (by tert-butyl hydroperoxide) (Fig 9E) and DNA damage (by UV) (Fig 9F) failed to alter *Phpk-1*::*HPK-1*::*GFP* levels or its pattern of expression. Thus, HPK-1 is specifically induced by thermal stress and this induction is post-transcriptional.

### HPK-1 is inhibited by TORC1 and is necessary for TORC1-associated longevity

**Fig 10:**
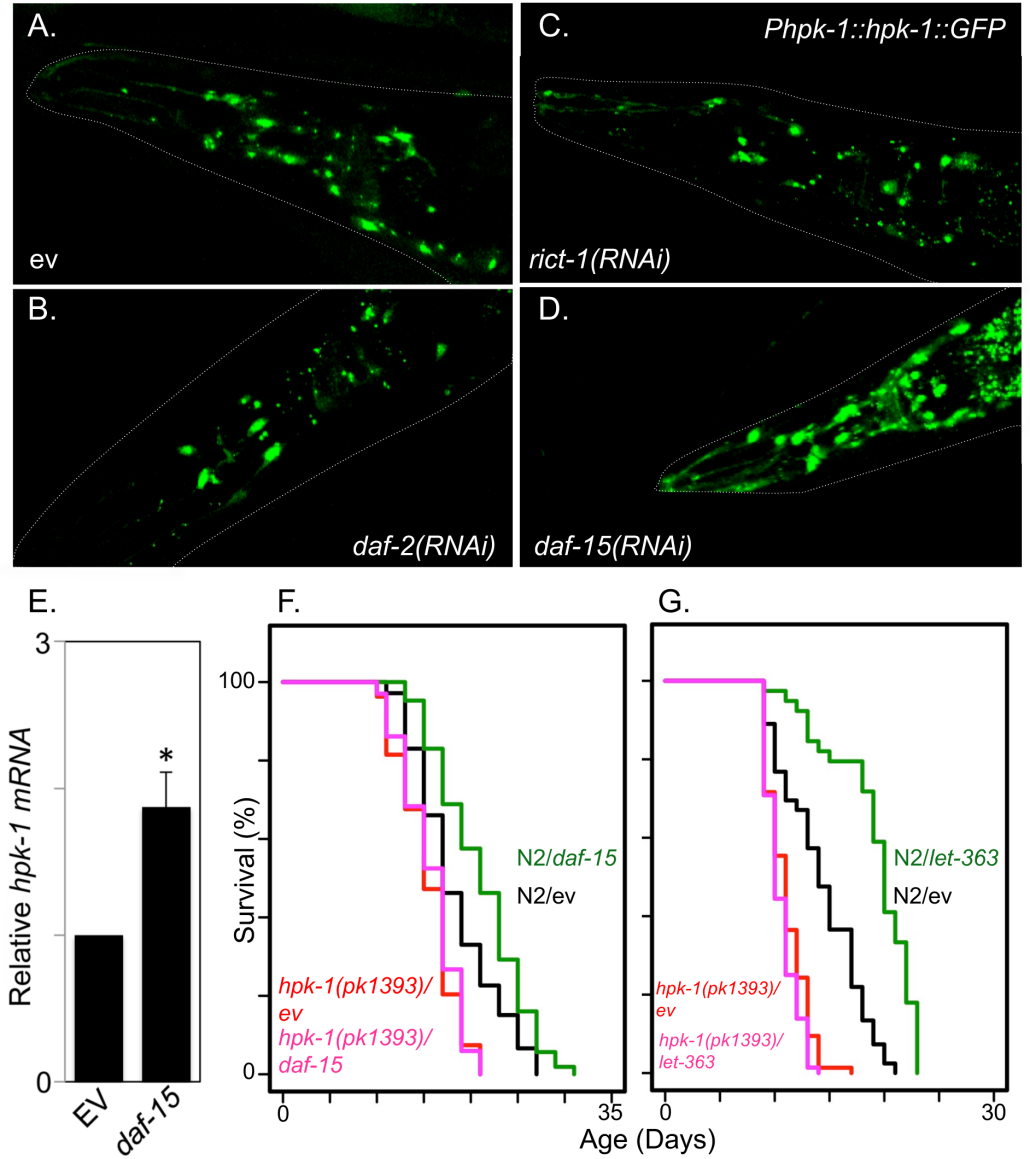
Decreased TORC1 activates HPK-1 to extend longevity. (A-D) Decreased TORC1 induces neuronal expression of HPK-1. Representative images of *Phpk-1*::*HPK-1*::*GFP* neuronal expression in day 3 adult animals after treatment with control (A), *daf-2* (B), *rict-1* (C) and *daf-15* RNAi (D). Outlines of animals are traced in white. White space was artificially filled for (D). Additional images and quantification can found in S9 Fig. (E) Induction of endogenous *hpk-1* after *daf-15* inactivation as measured by qRT-PCR. Values are mean fold change and S.E.M. Three independent experiments were performed and normalized to *cdc-42*. * indicates a p-value <0.05 (Student’s t-test). (F, G) *hpk-1* is essential for increased lifespan of *daf-15(RNAi)* and *let-363(RNAi)*-treated animals, respectively. Tabulated lifespan is provided in S1 Table.

In addition to heat shock, DNA damage, and oxidative stress, another type of stress that contributes to longevity is metabolic or nutritional stress. Many longevity transcription factors linked to environmental stress responses are also responsive to changes in nutritional status. For example, HIPK2 induction under conditions of glucose deprivation has been described in mammalian cell culture[59]. This suggested that HPK-1 expression might be inhibited by the insulin-like receptor (*daf-2*) and/or the TOR signaling complex, both of which control key nutritional responses in *C*. *elegans* and extend longevity when disrupted. We tested whether RNAi inactivation of *daf-2*, *daf-15* (corresponding to *raptor*, a TORC1 subunit), or *rict-1* (corresponding to *rictor*, a TORC2 subunit) altered HPK-1 expression. We observed no change in expression of the *Phpk-1*::*HPK-1*::*GFP* reporter in response to *daf-2* or *rict-1* inactivation, but we did observe a significant increase in HPK-1::GFP protein expression in head neurons in *daf-15(RNAi)* animals (Figs 10A–10D, S8A). HPK-1 induction was distinct mechanistically from heat shock induction of HPK-1 in two ways: 1) *hpk-1* mRNA levels were increased by inactivation of *daf-15* (Fig 10E) but not heat shock (S7C Fig), and 2) HPK-1 induction by *daf-15* RNAi treatment was restricted to neurons whereas heat shock induced a broader expression pattern (S9B–S9D Fig). *daf-15(RNAi)* and *let-363(RNAi)* (TOR kinase) have been shown to increase lifespan in wild-type animals[60, 61]. We subsequently asked whether *hpk-1* was necessary for the enhanced longevity of either *daf-15(RNAi)* or *let-363(RNAi)*-treated animals. We observed suppression of the extended longevity phenotype of *daf-15(RNAi)* or *let-363(RNAi)-*treated animals to the lifespan observed in *hpk-1* mutant animals alone (Fig 10F and 10G respectively), suggesting that *hpk-1* may be an inhibitory target of TORC1 that is critical for changes in longevity mediated by altered TOR signaling.

### HPK-1 promotes autophagy in response to dietary restriction and inactivation of TOR

**Fig 11:**
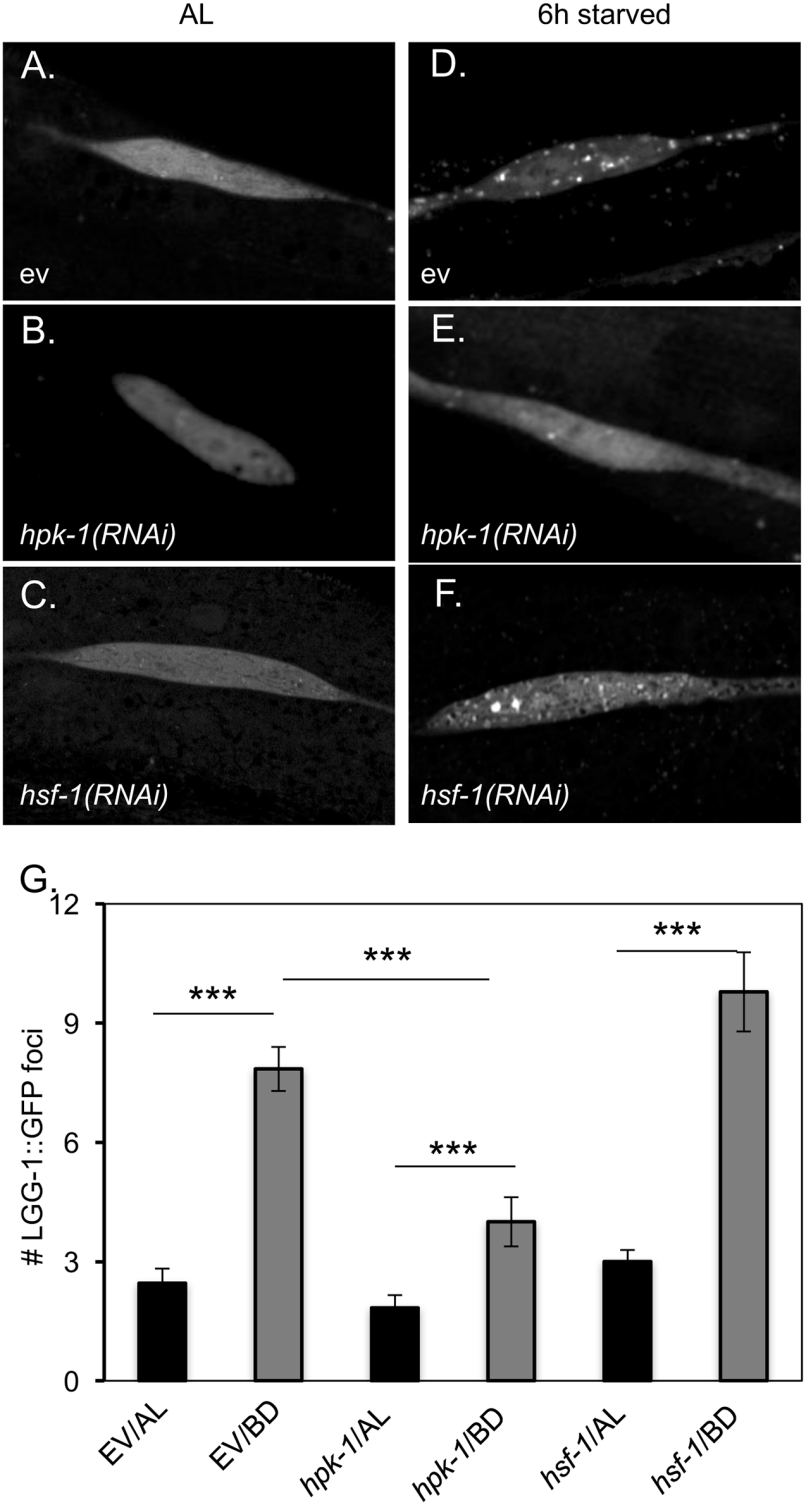
HPK-1 but not HSF-1 is essential for autophagosome formation. (A-F) Inactivation of *hpk-1* but not *hsf-1* disrupts autophagosome formation after bacterial deprivation (BD) as visualized by puncta formation for the autophagosomal reporter *Plgg-1*::*LGG-1*::*GFP* (Atg8p/MAP-LC3) [18]. (G) Quantification of LGG-1:GFP foci in L3 stage animals under *ad libitum* (AL) and bacterial deprivation (BD) conditions. BD was imposed by removal from bacterial food for 6 hours prior to scoring puncta formation. Plotted are the mean number of LGG-1::GFP puncta/seam cell visualized +/-S.D. *** indicates a p-value of <0.001 (Student’s t-test). Summary data provided in S4 Table.

Under nutrient rich conditions, TOR promotes cellular growth by activating protein translation (*e*.*g*. transcription of translation components) while inhibiting protein turnover (*e*.*g*. transcription of chaperones[62, 63] and autophagy genes[64], and the initiation of autophagy[65]). TOR inhibition, or genetic activation of any of these targets of TOR inhibition, results in extension of longevity[18, 60]. We tested whether regulation of any of these cellular processes was dependent on *hpk-1*. Autophagy is induced in response to fasting across many species, and can be visualized in *C*. *elegans* using the LGG-1::GFP reporter, in which GFP is C-terminally fused to the autophagosome component LGG-1 (i.e. LC3/Atg8 in mammals and yeast, respectively). Stimulation of autophagy is observed in epidermal seam cells during fasting as the formation of discrete LGG-1::GFP puncta[18]. We tested whether *hpk-1* was necessary for autophagosome formation following six hours of bacterial deprivation (BD) relative to replete, or *ad libitum* (AL) conditions. Consistent with published reports, LGG-1::GFP foci were rarely observed under *ad libitum* conditions (Fig 11A–11C), but were readily observable in seam cells upon bacterial deprivation (Fig 11D). We observed a near total loss of LGG-1::GFP foci formation in response to BD in *hpk-1(RNAi)-*treated animals (Fig 11E). Interestingly, LGG-1::GFP foci formation following bacterial deprivation did not require *hsf-1* (Fig 11F and 11G). This result reveals that autophagosome formation in hypodermal seam cells constitutes a biological function for HPK-1 that is separable from its role in regulating HSF-1 activity and HSF-1-dependent proteostatic outputs.

**Fig 12:**
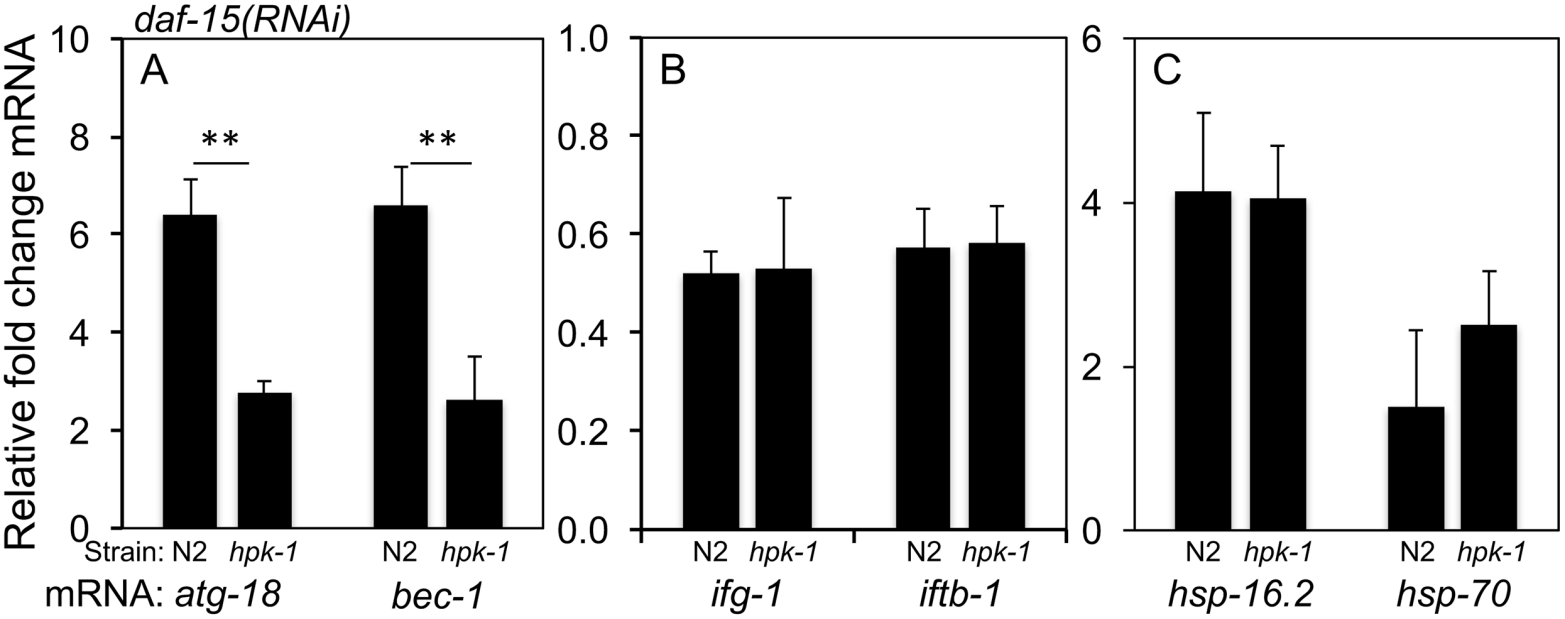
HPK-1 is essential for the transcriptional activation of autophagy. (A) *hpk-1* is necessary for the induction the autophagy genes *atg-18* and *bec-1* (Beclin1) in response to inactivation of TORC1 by *daf-15(RNAi)* (** indicates p<0.01, Student’s t-test). (B) In contrast, decreased TORC1 signaling represses the expression of the translation initiation factor genes *ifg-1* and *iftb-1* independently from *hpk-1*. (C) Similarly, TORC1 inhibition mildly induces *hsp-16*.*2* and *hsp-70* independently from *hpk-1*. Columns labeled *hpk-1* indicate *hpk-1(pk1393)*. Expression levels are presented as fold change +/- S.D. normalized to *cdc-42* and averaged across four independent experiments.

Because HIPKs are transcription factor regulators, we tested whether any of the genes negatively regulated by TOR require *hpk-1* for their expression. We observed that *hpk-1* is necessary for the induction of two autophagy genes (*atg-18* and *bec-1*) previously reported to be induced in response to *daf-15* inactivation and that are essential for autophagosome production (Fig 12A)[18, 20, 64, 66]. In contrast, loss of *hpk-1* had no effect on *daf-15(RNAi)-*induced down regulation of *ifg-1* and *iftb-1* (eIF-4G and eIF2beta, respectively), two translation initiation genes whose transcription is induced by active TORC1 (Fig 12B). Similarly, loss of *hpk-1* had no effect on the modest induction of molecular chaperones that occurs with TORC1 inactivation (Fig 12C), an induction known to be *hsf-1* dependent[63]. Therefore, a previously described interaction between TORC1 and HSF-1 leading to chaperone transcriptional induction is *hpk-1*-independent. Conversely, HPK-1 functions specifically in the autophagy axis of TORC1 signaling while *hsf-1* does not. Therefore, HPK-1 and HSF-1 must each have cellular functions that are distinct from each other in addition to their shared control of heat shock responses.

**Fig 13:**
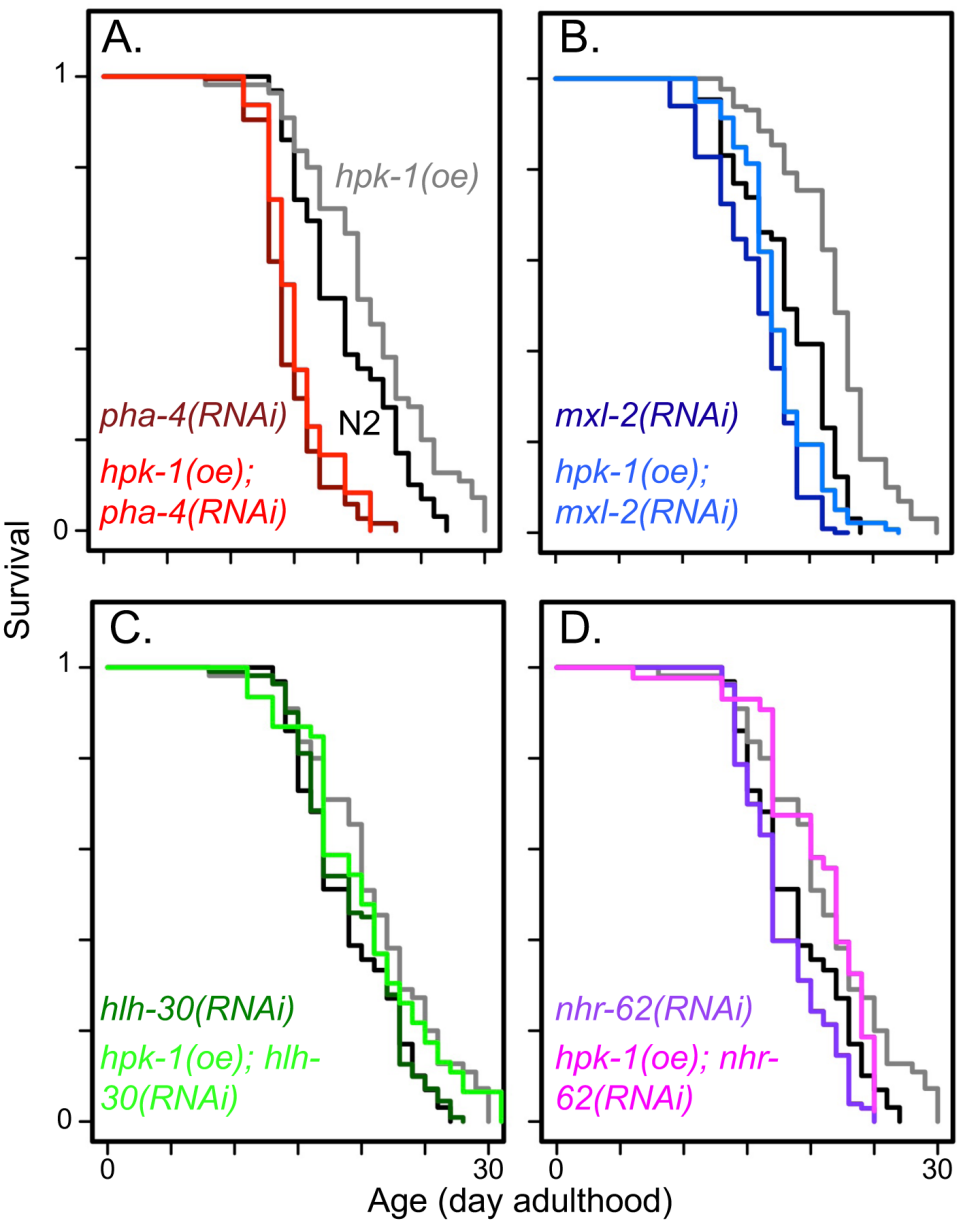
*pha-4* and *mxl-2* are required for *hpk-1* to promote longevity. (A) Overexpression of *hpk-1* increases lifespan (grey versus black) and is *pha-4* dependent (red traces). (B) Overexpression of *hpk-1* increases lifespan dependent on *mxl-2* (blue traces). (C) Loss of *hlh-30* (green traces) partially suppresses the increased lifespan of *hpk-1*, consistent with parallel signaling or independence. (D) Loss of *nhr-62* (pink/purple traces) has a minimal negative effect on both normal and the increased lifespan conferred by *hpk-1* overexpression. In all cases, black traces are N2 and grey traces are *Psur-5*::*HPK-1*::*CFP* animals treated on control RNAi. For each panel, darker colored traces are respective RNAi-treatment of N2 animals and lighter colored traces are RNAi treatment of *Psur-5*::*HPK-1*::*CFP* animals. In some cases, experiments shown within this figure were performed simultaneously and split into multiple figures for readability. Full lifespan data can be found in S1 Table.

Our result that HPK-1 promotes autophagy through a mechanism independent of HSF-1 suggests that HPK-1 regulates at least one additional transcription factor that is necessary for autophagy induction in response to DR or TORC1 inactivation. PHA-4/FoxA, HLH-30/TFEB, NHR-62/HNF4-related nuclear hormone receptor are transcription factors known stimulate autophagosome assembly[18, 22, 64, 66–68]. MXL-2/Mlx (part of the MML-1/(MondoA/ChREBP) complex) has been implicated in autophagy regulation because it is the binding partner of MML-1, which promotes autophagy gene expression upon inactivation of TOR[21]. As we are primarily concerned with those functions connecting TOR and HPK-1 activity to lifespan, we examined whether the increased lifespan arising from HPK-1 overexpression (Fig 1B) was suppressed by inactivation of any of the above transcription factors. Of these, we found that inactivation of *pha-4* or *mxl-2* is epistatic to *hpk-*1 overexpression, suppressing the increased lifespan conferred by *hpk-1* overexpression (Fig 13A and 13B). In contrast, animals overexpressing *hpk-*1 were still long-lived after inactivation of *hlh-30* or *nhr-62* (Fig 13C and 13D). Thus, *hpk-1* overexpression extends longevity in a manner dependent on *pha-4* and *mxl-2*, but not *nhr-62* or *hlh-30*.

**Fig 14:**
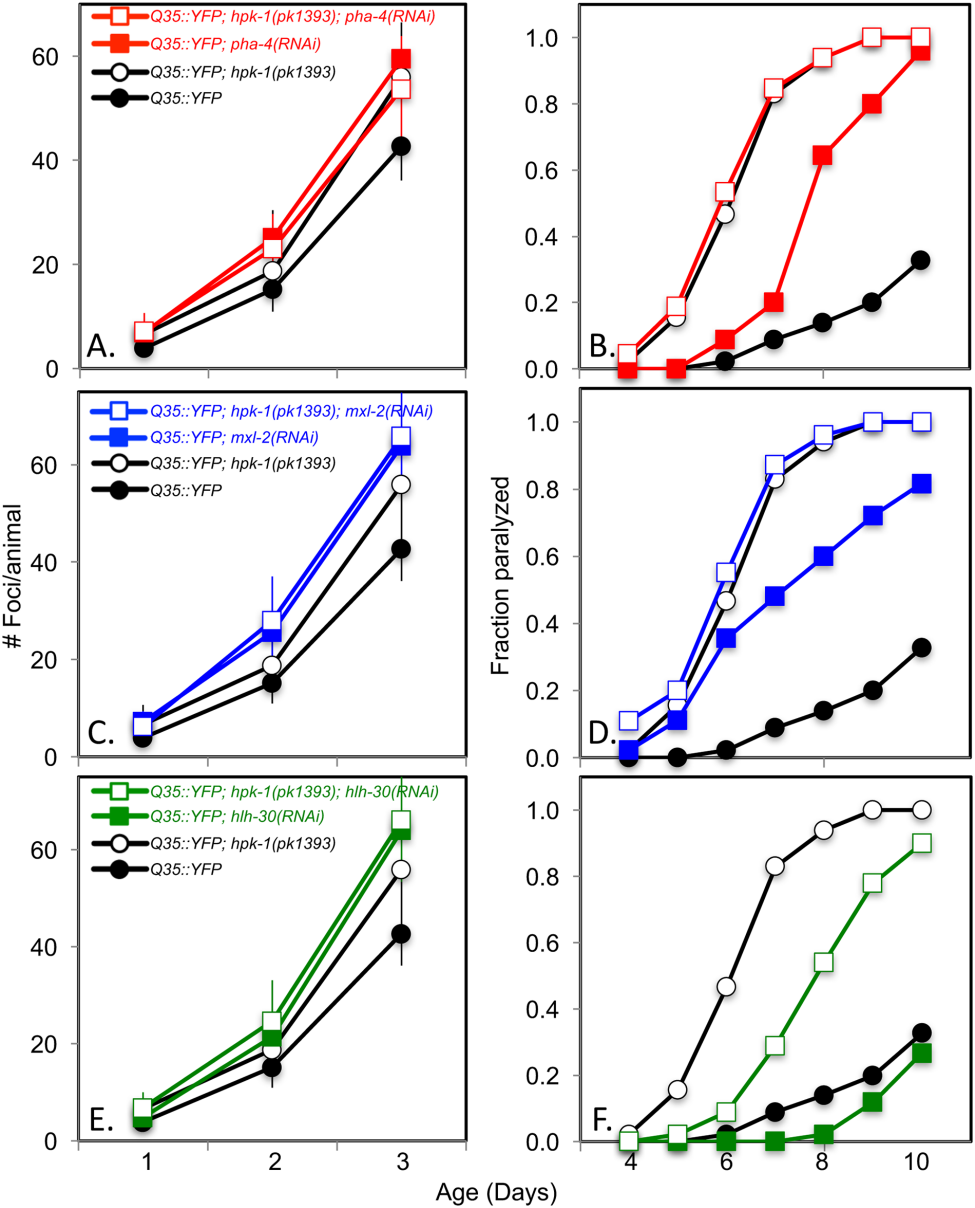
*pha-4* and *mxl-2* intersect with *hpk-1* in the maintenance of proteostasis. (A-B) Loss of *pha-4* (red traces) does not increase foci formation (A) or the onset of paralysis (B) in the absence of *hpk-1* (open circles/squares). (C-D) Loss of *mxl-2* (blue traces) does not increase foci formation (C) or the onset of paralysis (D) in the absence of *hpk-1* (open circles/squares). (E-F) Loss of *hlh-30* (green traces) does not increase foci formation (E) but delays the onset of paralysis in the absence of *hpk-1* (F) (open circles/squares). For foci formation, data are the mean and standard error of the mean (S.E.M.) of at least 15 animals from one representative trial; three independent experiments were performed. ***, **, and * indicate p-values of <0.001, <0.01, and <0.05, respectively. For paralysis, data is representative of one of two trials performed with the same conditions. See S3 Table for additional details.

Autophagy has been shown to ameliorate aggregate formation in response to polyglutamine tracts in *C*. *elegans*, as well as other systems[69–71]. We therefore tested whether these autophagy-inducing transcription factors mitigate polyQ aggregate formation and/or toxicity in muscle cells, as has been reported in two other studies[55, 72], and whether their ability to do so requires *hpk-1*. Inactivating *pha-4* or *mxl-2* in otherwise wild-type *Q35*::*YFP* animals resulted in both accelerated protein aggregate formation and early onset of paralysis (Fig 14A–14D), consistent with previous findings. Curiously, inactivation of *hlh-30* also accelerated Q35 aggregate formation without enhancing its associated locomotory toxicity.

Next, we wished to determine if these transcription factors act in parallel or as part of an *hpk-1* regulatory network that promotes protective proteostatic responses to oppose the aggregation-associated phenotypes of polyglutamine repeats. Loss of either *pha-4* or *mxl-2* did not worsen the accelerated polyglutamine phenotypes of an *hpk-1(pk1393)* mutant, suggesting that *pha-4* and *mxl-2* protect against aggregate formation in conjunction with *hpk-1*, possibly as direct transcription factor targets. In contrast, the *hlh-30* results were equivocal: while *hlh-30* RNAi did not accelerate the formation of Q35::YFP aggregates in the absence of *hpk-1*, *hlh-30* RNAi surprisingly resulted in partial restoration of the premature aggregate-associated motility defects of *hpk-1(pk1393)*. The apparent contradiction that loss of *hlh-30* ameliorates the proteotoxicity of an *hpk-1(pk1393)* mutant will require additional experimentation to determine the regulatory relationships between *hlh-30*, *hpk-1*, *mxl-2*, and *pha-4*. Nevertheless, our results- which tentatively place *hpk-1* in a regulatory network with *mxl-2*/*pha-4* but not *hlh-30*- are consistent with previous reports that have found *hlh-30* and *pha-4* have distinct pro-longevity functions[19] and *pha-4* and *mxl-2* have similar pro-longevity functions[72]. It is tempting to speculate that loss of *hlh-30* may be compensated by upregulation of MXL-2 or PHA-4, which partially rescues the proteotoxicity that results from loss of *hpk-1*.

Bacterial deprivation is known to activate autophagy and the nuclear translocation of DAF-16/FoxO and HLH-30/TFEB[19, 73]. Since *hpk-1* is required for autophagosome formation after bacterial deprivation, we tested whether *hpk-1* was also required for DAF-16 or HLH-30 activation. Loss of *hpk-1* did not impair cytoplasm-to-nuclear translocation of HLH-30::GFP or DAF-16::GFP after bacterial deprivation (S10 and S11 Figs). This is consistent with the notion that *hpk-1* and *daf-16/hlh-30* have separable functions in nutrient sensing. Collectively, our results support a model in which PHA-4 and MXL-2 represent a nutritionally-responsive arm of HPK-1 regulation in addition to its role in HSF-1 activation in response to thermal stress and in preserving the integrity of the proteome under unstressed conditions.

## Discussion

### HPK-1 belongs to a conserved family of homeodomain-interacting kinases that increase resistance to thermal and nutritional stresses

In this study we describe HPK-1 as a transcriptional regulator of proteostasis and longevity in *C*. *elegans*. HPK-1 is the lone representative of the homeodomain-interacting kinase gene family in *C*. *elegans*, a relatively understudied family of kinases. *hpk-1* is most closely related to yak1 in *Saccharomyces cerevisiae* and to the HIPKs and DYRK kinases in mammals. In mammals a total of four HIPK orthologues respond to a number of external cues including the DNA damage response, hypoxia response, reactive oxygen species (ROS), glucose availability, and viral infection[32, 45, 74, 75]. In general HIPK family members regulate the activity of transcription factors, chromatin modifiers, signaling molecules and scaffolding proteins in response to cellular stress. For example, genotoxic damage induces mammalian *Hipk2*, and HIPK2 potentiates p53 pro-apoptotic activity through direct phosphorylation[34].

We originally identified *hpk-1* in an RNAi screen for genes acting in the *daf-2*/insulin signaling pathway (i.e. genes necessary for the increased lifespan of *daf-2* mutants). A subset of the gene inactivations that shorten *daf-2(e1370)* lifespan also confer no additional lifespan shortening effect in a *daf-2;daf-16* genetic background[38], implying function specifically within the ILS pathway. *hpk-1(RNAi)* met these phenotypic criteria, but we ultimately concluded that *hpk-1* was unlikely to be component of the canonical DAF-2(insulin/IGF1R)-AGE-1(PI3K)-AKT-DAF-16(FoxO) signaling pathway, because loss of *hpk-1* only modestly suppressed *daf-2* lifespan, and to a degree that was not proportionally greater than the reduction of lifespan observed in response to *hpk-1* inactivation in wild-type animals[38]. Additionally, induction of the DAF-16 target gene *sod-3* under conditions of decreased ILS does not require *hpk-1*[38]. Our conclusion has since been supported by a separate study reporting that only eight of 259 DAF-16/ILS regulated genes showed decreased expression in animals lacking *hpk-1*[42]. Lastly, in this manuscript we show that decreased ILS does not alter *hpk-1* expression (Fig 10B). Collectively these findings are more consistent with a model where HPK-1 functions not within but parallel to the canonical ILS pathway.

### HPK-1 extends longevity, thermotolerance and preservation of proteostasis by activation of HSF-1

We show that overexpression of *hpk-1* extends natural longevity, which suggests that HPK-1 exerts a regulatory function on longevity pathways, rather than simply being required for some essential physiological function. We show that a translational HPK-1::GFP reporter is expressed broadly during *C*. *elegans* embryogenesis and larval development (in intestine, hypodermal seam cells and neurons), but its expression pattern becomes restricted to neurons in adults. These data are consistent with reported patterns of HIPK expression in mammals and *C*. *elegans* [76, 77]. Because stress response pathways are intimately tied to longevity, we tested whether *hpk-1* expression was induced by oxidative stress, DNA damage or heat shock. We found that heat shock induces HPK-1 in the same tissues where it is normally expressed during development: hypodermis and neurons, and to a lesser extent, the intestine. We showed that heat shock induction was at the protein level, as it occurred in the presence of α-amanitin. A role for HPK-1 in heat shock responses is likely to be evolutionarily conserved, as *yak1* is also induced by heat stress and is required for normal thermal stress survival in *S*. *cerevisiae* [78, 79]. In contrast, we observed no induction of HPK-1 in response to oxidative or DNA damage.

We investigated whether *hpk-1* functions as part of the well-known HSF-1 heat shock response pathway. We found that *hpk-1* was required for HSF-1 mediated thermotolerance, the hormetic extension of longevity, and transcriptional induction of two key HSF-1 target genes, the chaperones encoded by *hsp-16*.*2* and *hsp-70*. *hsf-1* and *hpk-1* are mutually required for the increased lifespan arising from overexpression of the other. We observed that an HPK-1::GFP fusion protein was localized to subcellular structures coincident with HSF-1 localization, an observation consistent with a model in which HPK-1 and HSF-1 reside within a regulatory complex. We believe that HPK-1 is likely to be a positive regulator of HSF-1 activity because of its homology to kinases that activate transcription factors by phosphorylation. The homology of HPK-1 to the homeodomain-interacting kinase family, together with our result that HPK-1 induction was not blocked by global inhibition of transcription meant that we were unsurprised to discover that the heat shock inducibility of HPK-1 was also HSF-1-independent,

A separate study has recently reported that *C*. *elegans* HPK-1 is induced by thermal stress. However the authors concluded that *hpk-1* was not part of the HSF-1 heat shock response because they saw no dependence on *hpk-1* for induction of endogenous molecular chaperones after heat shock[42]. We believe we can reconcile this discrepancy as a consequence of the timing at which chaperone induction was measured. We heat shocked animals and examined endogenous chaperone induction during late larval development, while Berber et al. tested for chaperone induction after the onset of reproduction. However, the onset of reproductive maturity in *C*. *elegans* is characterized by an extreme downshift in the ability of animals to respond to heat shock. Within 4 to 8 hours of the onset of reproduction, repressive chromatin marks are laid down at stress loci; these repressive chromatin modifications severely curtail the ability of animals to respond to heat shock[30]. This seems a likely explanation for why Berber et al. observed only low-level induction of *hsp-70* and *hsp-16* in response to heat shock, and why they were unable to detect a dependency on *hpk-1*. In contrast, we conducted our experiments prior to the timing of chromatin repression at heat shock loci. Resultantly, we observed a far larger transcriptional induction for *hsp-70* and *hsp-16*.*2* compared to Berber et al. (~100 and 4500-fold, respectively, versus ~6–8 fold) and nearly all of this induction required the presence of *hpk-1*. This suggests that *hpk-1* is essential for the activation of the heat shock response prior to chromatin silencing at stress loci, but after the heat shock response is compromised through chromatin remodeling at stress loci, *hpk-1* is no longer essential for the limited transcriptional activation of heat shock genes. As Berber et al. did not report whether the residual expression of chaperones they observed requires *hsf-1* itself, it is impossible to say whether this modest transcriptional induction is HSF-1 mediated or occurs through some other mechanism.

HSF-1 promotes global proteostasis even in the absence of heat stress by regulating the transcription of chaperones, which maintain stability and solubility of the proteome both by assisting in the folding of newly translated polypeptides and by directing chronically misfolded proteins to the proteasome for degradation. The protective functions of the chaperone system are particularly relevant to diseases of the nervous system caused by inappropriate protein aggregation and resulting neural toxicity. Expression of aggregation-prone polyQ transgenic constructs in *C*. *elegans* provides both a method for detecting the proteostatic stress level of tissues and a means of identifying protective and risk factors for aggregation diseases. Inactivation of *hsf-1*, for instance, causes a premature accumulation of polyglutamine-YFP puncta in muscle cells[80].

When we tested whether HPK-1 displays a similar protective effect on polyQ aggregate formation, we found that HPK-1 is as, if not slightly more important than, HSF-1 in its ability to delay insoluble aggregate formation and associated paralysis in Q35:YFP animals (see S3 Table). Moreover, *hsf-1* RNAi did not increase the number or toxicity of polyQ aggregates when combined with the *hpk-1(pk1393)* mutation, showing that HSF-1 confers its protective effects entirely under the regulatory umbrella of HPK-1. Strikingly, HPK-1 overexpression exerted a potent protective effect against polyQ proteotoxicity by dramatically reducing the rate of foci formation and paralysis.

### HPK-1 opposes inhibitory sumoylation of HSF-1

There has been significant effort invested in defining the mechanism(s) of activation of the heat shock transcription factor HSF-1, an effort complicated by the large number of post-translational modifications on HSF-1. Biochemical analysis of HSF-1 regulation is an extremely active area of research from yeast to mammals. Most studies of HSF-1 activation have been performed in tissue culture and *ex vivo* models, but there is very little information about the regulation of HSF-1 by post-translational modification in living animals. In this study, we describe a pair of HSF-1 modifications in *C*. *elegans* that correlate with reduced transcriptional activity of HSF-1 during aging, and in a manner dependent on *hpk-1*.

Since HPK-1 is a protein kinase that co-localizes into subnuclear foci with HSF-1, the simplest model is that HPK-1 stimulates HSF-1 activity through direct phosphorylation. There are multiple examples in mammals that phosphorylation of a transcription factor/co-factor directly prevents subsequent sumoylation, thereby increasing the activation potential of that transcription factor; examples include PML protein, p53, and c-Jun[81, 82]. While we were unable to resolve a phospho-isoform of HSF-1 attributable to HPK-1 activity, not all phosphorylation events produce a mobility shift. Alternatively, our antibody may not recognize the relevant phospho-isoform of HSF-1.

It would also be informative to test whether HSF-1 regulation requires an active HPK-1 kinase domain. There are at least 19 phosphorylation sites on human HSF1, a subset of which are conserved in *C*. *elegans* (S5 Table and[41]). Consistent with the possibility of direct interaction between HPK-1 and HSF-1, *S*. *cerevisiae* Yak1 directly phosphorylates Hsf1 in response to glucose stress, albeit within a region not conserved in multicellular eukaryotes [36]. Alternatively, HPK-1 may act indirectly to prevent HSF-1 sumoylation. In mammals both sumoylation and acetylation occurs at HSF-1 K298[49], the latter through the opposing acetyltransferase activities of p300 and SIRT1[41]. Consistent with the possibility of indirect interaction, mammalian HIPK2 directly phosphorylates SIRT1 to restrict activity after DNA damage[83]. Given the complexity of post-translational modification to HSF-1, determination of whether HPK-1 directly phosphorylates HSF-1, and identification of the relevant residue(s) will require analysis using mass spectrometry.

Sumoylation of transcription factors is often associated with reduced transactivation activity [84]. Specifically, sumoylation of HSF family members has previously been reported in mammals, and these isoforms possess decreased transcriptional activity[39, 85]. Our findings that *smo-1(RNAi)* leads to an increase in HSF-1 chaperone induction in response to heat shock support a hypothesis in which sumoylation of HSF-1 is inhibitory in *C*. *elegans* as well. Moreover, our data support a role for HPK-1 in preventing HSF-1 sumoylation (and its coupled phosphorylation event) through the end of development, at which point HPK-1 becomes restricted to low level expression in neurons. This timing for the loss of systemic HPK-1 expression correlates with the onset of transcriptional silencing at stress loci[30] and a decline in multiple additional protein quality control mechanisms (reviewed in[29]). Opposition of sumoylation by HPK-1 may provide a molecular mechanism for a long-standing question in the aging field concerning the decline of HSF-1 activity in aging animals. In mammals, HSF1 DNA binding activity and chaperone expression levels both decline with aging, while the abundance of HSF1 protein does not[41]. We propose that sumoylation of HSF-1 is inactivating, and that increased accumulation of sumoylated HSF-1 with reproductive age explains the decline of basal HSF-1 activity and the resulting decay of proteostasis in aging animals. We also propose that a critical longevity function of HPK-1 is to protect HSF-1 against age-dependent inactivation—by delaying/preventing sumoylation or by driving de-sumoylation.

### HPK-1 stimulates transcriptional induction of autophagy in response to dietary restriction. TORC1 blocks autophagy by inhibition of HPK-1 expression in the nervous system

We describe earlier in the discussion how we initially identified a role for *hpk-1* in longevity control, and provide the rationale for our conclusion that the pro-longevity functions of *hpk-1* belong to pathway(s) acting in parallel to the ILS/FOXO signaling pathway. We have shown that *hpk-1* promotes HSF-1 mediated transcription—within the context of the heat shock pathway and basal unstressed conditions—to promote global proteostasis via chaperone systems. We also wondered if *hpk-1* might function more broadly to regulate stress-responsive transcription factors. We suspected that *hpk-1* might modulate nutritional stress responses controlled by the TOR signaling pathway. First, a wealth of data in mammals and *C*. *elegans* places *daf-15* (Raptor, a TORC1 subunit) in a pathway parallel to ILS based on distinct activities within each pathway that modulate longevity[60]. However, *daf-2*/ILS and *daf-15*/TOR do not always function in isolated linearity with respect to each other, and under certain conditions these kinases converge on common transcription factors necessary for extension of longevity, including SKN-1(Nrf2), HSF-1, MXL-2, and DAF-16[21, 50, 63, 72, 73, 86–88]. Only a subset of transcriptional targets is shared. For example, TORC1 inactivation does not stimulate DAF-16 nuclear translocation and activation (as *daf-2* inactivation does)[73]. Though the importance of HIPKs in orchestrating stress responses has only begun to emerge, several pieces of evidence suggest that HIPKs may reside at the crossroads of ILS and TOR signaling. In mammals, multiple mechanisms link insulin/IGF (insulin-like growth factor) levels to activation of mTORC1[89]. Knockdown of HIPK1, 2 or 3 attenuates insulin secretion in response to glucose and HIPKs bind to insulin promoters, suggesting that HIPKs may activate insulin expression[37]. Glucose deprivation can also activate Hipk1[75, 90]. In budding yeast, the HIPK homolog Yak1 is activated by rapamycin treatment[91] and by glucose depletion[92]. For these reasons, it seemed worth testing if *hpk-1* might function within the TOR pathway, potentially as a point of regulation between TOR growth signaling and the nutrient deprivation transcriptional programs repressed by TOR.

First, we found that inactivation of *daf-15* stimulated neuronal expression of HPK-1, while inactivation of *daf-2* did not. HPK-1 induction in response to *daf-15* RNAi was restricted to the nervous system, in contrast to the broader induction of HPK-1 in response to heat shock that also included HPK-1 expression in seam cells and the intestine (weakly). When we examined the transcriptional programs controlled by TOR, we found that HPK-1 was necessary for the induction of autophagy genes *bec-1* and *atg-8* following TOR inactivation, but that *hpk-1* had no effect on the transcription of translation initiation factor genes induced by TOR activation. In further support that HPK-1 can stimulate autophagy, we observed that the induction of autophagosome formation (LGG-1::GFP) in response to DR required *hpk-1* while interestingly, *hsf-1* was not required. Stimulation of autophagy by *hpk-1* in response to DR must therefore involve the activation of other transcription factor(s) that are not HSF-1.

Autophagy is thought to occur in at least two separate phases: a rapid increase in autophagic flux that occurs entirely through post-translational protein modifications within minutes to hours after stress, which is followed by a subsequent extended phase reliant on the activation of transcriptional programs[17, 64]. Dietary restriction and TOR inactivation induce protein turnover in *C*. *elegans* by stimulating autophagy, which requires multiple DR-responsive transcription factors, including: *pha-4(FoxA)*, *mxl-2(MLX)*, *hlh-30(TFEB)* and *nhr-62(NHR4α)*. In addition to transcriptional controls, the TORC1 complex inhibits autophagy directly by inhibitory phosphorylation of autophagy components necessary for initiation of autophagy. Because *hpk-1* functions biologically by activation of transcription factors, it seems likely that HPK-1 acts during the “extended phase” of autophagy induction by activating one or more of the transcription factors required for autophagy induction in response to DR.

### Distinct roles for HSF-1 and HPK-1 in autophagy induction in response to thermal and nutritional stress

Our results are consistent with a general model in which HPK-1 promotes protein homeostasis by two separate mechanisms, each of which can function under basal as well as stressed conditions. HPK-1 stimulates transcription of molecular chaperones via HSF-1, which decreases the physiological load of misfolded proteins *in vivo*. In parallel, HPK-1 stimulates autophagy via PHA-4 and MXL-2, allowing existing proteins to be catabolized into useable metabolites, also reducing the physiological protein load. Under conditions of heat shock, the requirement for HPK-1 becomes more pronounced as the need for a proteostasis compensation mechanism is dramatically increased in response to protein unfolding. Under conditions of nutrient stress, proteostasis is not directly compromised, but the demand for metabolic building blocks must be met using existing cellular resources. In this case, protein turnover by autophagy may be an effective way to supply essential metabolites; at the same time that it improves proteostasis by clearing protein aggregates.

A recent study identified a role for HSF-1 in the induction of autophagy after hormetic heat shock or ectopic over-expression of *hsf-1*[70]. Our finding that *hpk-1* is essential for the beneficial effect of hormetic heat shock on lifespan (S4G Fig) would be consistent with the notion that *hpk-1* may also be essential for the induction of autophagy gene expression conferred by either *hsf-1* overexpression or hormetic heat shock, as well as after nutrient stress. Induction of autophagosome formation may also be regulated differently in different tissues: we find that *hpk-1* but not *hsf-1* is required for autophagosome formation in hypodermal seam cells, while heat mediated autophagosome formation in muscle, intestinal, and cells within the nerve ring require *hsf-1*[70].

### Extension of longevity and proteome maintenance by HPK-1 requires the autophagy-inducing transcription factors PHA-4 and MXL-2

Upon nutrient deprivation, *hlh-30* (TFEB), *pha-4* (FoxA), the nuclear hormone receptor *nhr-62*, and the Myc family transcription factors *mml-1* (Mondo A/ChREBP) and *mxl-2* (Mlx) have all been shown independently to be essential for at least one aspect of the induction of autophagy[21, 22, 64]. TORC1 inactivation is thought to increase lifespan by inducing autophagy and by decreasing global protein translation (reviewed in[65]). Dietary restriction reduces TOR signaling[93]. We and others have shown that either conditions of dietary restriction or inactivation of TORC1 depend upon *hpk-1*, *pha-4* and *mxl-2* to extend longevity (this study and[21, 67, 72, 73]). Similarly, we show that the extension of longevity conferred by overexpression of *hpk-1* is suppressed by *pha-4* and *mxl-2*, but not *hlh-30* or *nhr-62* (Fig 13), suggesting that autophagy contributes to the extended longevity of animals overexpressing HPK-1 in addition to the induction of chaperone genes driven by HSF-1. HPK-1 could represent the crucial regulation point between TORC1 and its target transcription factors PHA-4 and MXL-2. We have demonstrated that TOR inhibits expression of *hpk-1* mRNA (Fig 10E) and protein levels of HPK-1 (Figs 10D, S8). Mammalian Hipks are unstable and HPK-1 could be inhibited by TOR post-translationally through regulation of its stability. A particularly exciting possibility is that HPK-1 acts as a nutritional switch operated by TOR. For instance, phosphorylation of HPK-1 by TOR may destabilize HPK-1 and/or target it for degradation. Under replete conditions, autophagy gene transcription would be OFF because HPK-1 protein levels are being maintained at only low levels by TOR kinase. Inactivation of TOR kinase by dietary restriction would lead to stabilization of HPK-1, allowing protein levels to accumulate. HPK-1 could in turn phosphorylate and activate downstream transcription factors (PHA-4 and MXL-2) that stimulate expression of genes necessary for autophagosome biogenesis.

A complication of this model is that it only provides a sequence of action for TOR, HPK-1 and PHA-4/MXL-2 when they are functioning within the same tissue. We have shown that *TORC1*(RNAi) stimulates HPK-1 expression in neurons, while the Q35::YFP transgene is only expressed in muscle. Therefore, it is likely that endocrine signals arise downstream of HPK-1 in order to regulate cellular functions like autophagy in non-neuronal tissues. Precedent for the neuroendocrine relay of stress responses has arisen in recent years, including the unfolded protein responses of the ER UPR^ER^, mitochondria (UPR^mito^), and the heat shock response[25, 94–96]. Activation of either of these stress response pathways in neurons stimulates activation of the self-same UPR in non-neuronal tissues, and a recent report suggests that the transcription factor actors of these responses are required both “upstream” (in neurons) and “downstream” (in non-neuronal tissues) in the neuroendocrine circuit. A specific example is stimulation of the ER^UPR^ in non-neuronal tissues by activation of the UPR^ER^ in neurons. XBP-1, a cell autonomously acting transcription factor is required in both neurons and non-neuronal tissues in order for the UPR^ER^ activation in neurons to elicit the UPR^ER^ in distal tissues. Aside from evidence that serotonergic signaling is involved, the mechanism(s) by which specific cellular stress response pathways send or receive endocrine signals between tissues in order to generate systemic responses has yet to determined[97, 98].

### Activation of PHA-4 and MXL-2 transcriptional programs by HPK-1 protects animals from polyglutamine aggregate formation

In addition to evaluating the requirement for autophagy transcription factors in mediating the increased lifespan arising from HPK-1 overexpression, we tested the possibility that PHA-4, MXL-2 and HLH-30 were targets of HPK-1 regulation in the proteostatic context of polyQ aggregation. First we quantitated the dynamics of Q35::YFP aggregate formation and toxicity in the presence or absence of *pha-4*, *mxl-2* and *hlh-30* using RNAi. We found that inactivation of all three transcription factors significantly accelerated the accumulation of Q35::YFP aggregates. Inactivation of *pha-4* and *mxl-2* accelerated the rates of toxicity-induced paralysis. Curiously, *hlh-30* RNAi partially rescued the paralysis phenotype of the *hpk-1(pk1393)* mutant. In the Q35::YFP; *hpk-1(pk1393*) background, inactivation of *pha-4*, *mxl-2* or *hlh-30* did not increase aggregate formation above the already elevated level caused by the absence of *hpk-1*. Similarly, *pha-4(RNAi)* and *mxl-2(RNAi)* had no increased effect on the accelerated rates of paralysis observed in the *hpk-1(pk1393*) mutant strain relative to wild type. Our finding that inactivation of *pha-4* and *mxl-2* causes an increase in aggregate formation suggests that autophagy can in fact mitigate the formation of toxic Q35::YFP aggregates in muscle cells. This supports our model: extension of longevity depends specifically on the stimulation of autophagy by *hpk-1*—via activation of the *pha-4* and *mxl-2* transcription factors—and suggests that HPK-1, PHA-4, and MXL-2 may comprise a larger transcriptional circuit to regulate autophagy gene expression under specific conditions of nutrient stress.

### Proteostatic robustness is determined by the mutual opposition of TOR-mediated growth signaling and HPK-1-mediated stress responses

Aging is highly dependent upon the cellular processes that maintain proteostasis[99, 100], and diverse age-related neurodegenerative diseases (e.g. Alzheimer, Parkinson and Huntington diseases) are linked to compromised autophagy and the decline of proteostasis[99–102]. Proteostasis is achieved by the careful balancing of rates of protein synthesis with chaperone-mediated protein folding and turnover of unfolded polypeptides via proteasome- and autophagy-mediated degradation pathways. When proteostability is compromised, HSF-1 promotes restoration of proteostasis by induction of molecular chaperones and proteosomal degradation of misfolded proteins[49]. In contrast, nutrient rich conditions challenge proteome stability by activation of the TOR pathway, which stimulates protein translation and inhibits protein turnover. When nutrients are limited, TOR kinase is inactivated, prompting protein degradation through autophagy into constituent metabolites, which serves the dual function of reducing the overall concentration of proteins that must remain soluble while providing biosynthetic building blocks that can be remobilized towards pro-survival processes[103]. It is worthy of notice that HPK-1 and TOR act in physiological opposition; HPK-1 promotes catabolic processes that decrease proteotoxic stress, while TOR promotes an anabolic physiological state represented by an overall increase in cellular protein. Overexpression of HPK-1 or inhibition of the TOR pathway promotes proteome re-stabilization and concomitant extension of longevity.

### Significance: Proteostasis in the context of aggregation diseases

**Fig 15:**
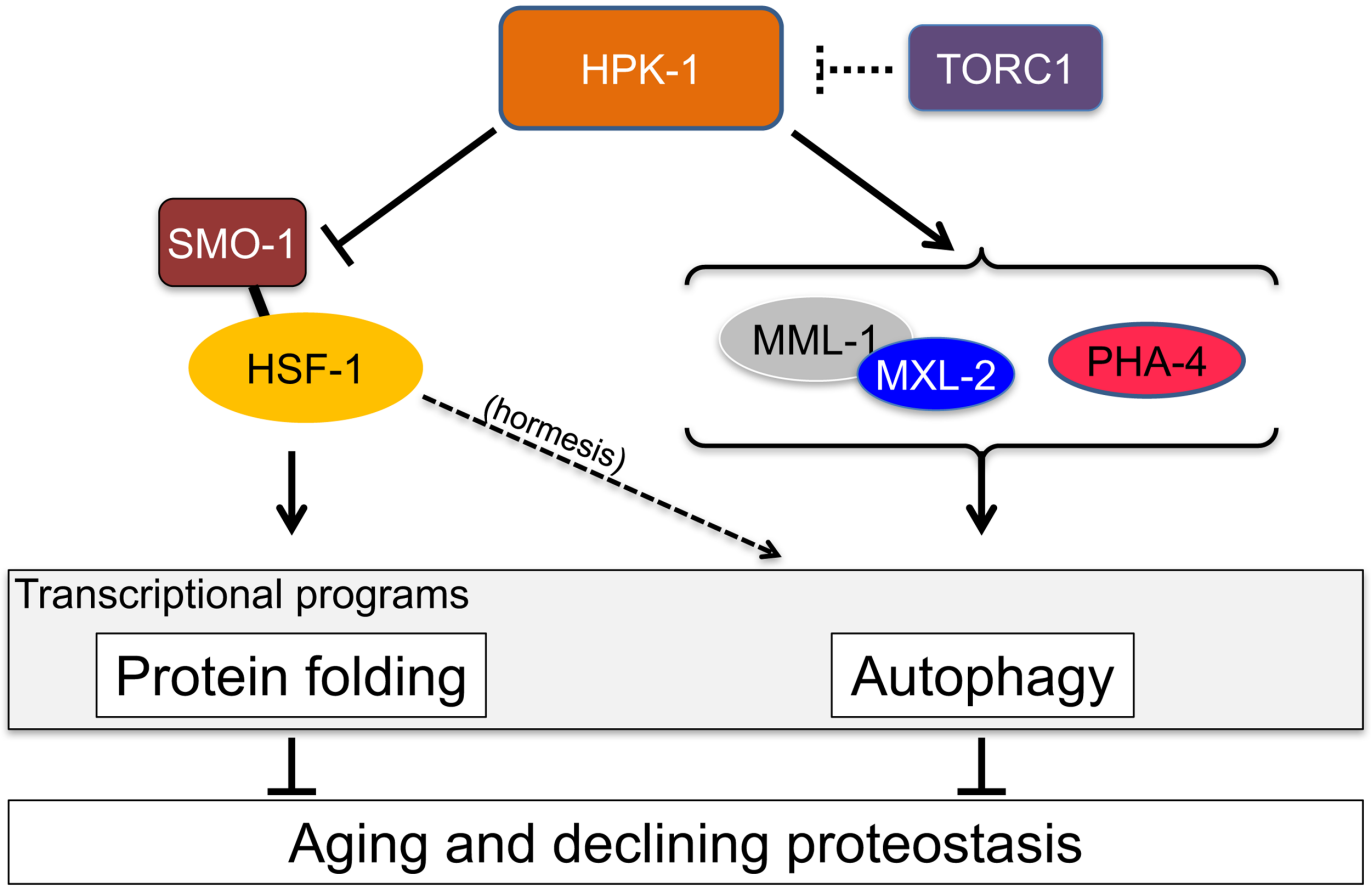
HPK-1 delays aging and maintains proteostasis by potentiating TORC1 mediated autophagy and blocking HSF-1 inactivation through sumoylation. Model of HPK-1 functions in longevity control: HPK-1 functions as a central hub to maintain proteostasis by preventing sumoylation and inactivation of HSF-1 and by stimulating the expression of autophagy genes by *pha-4* and *mxl-2*. TORC1 inhibits *hpk-1* expression to limit the induction of autophagy genes under basal conditions. Under nutrient stress, TORC1 is inactivated resulting in increased *hpk-1* expression, which promotes autophagy gene expression through PHA-4/FoxA and MXL-2/Mlx. Thermal stress increases HPK-1 protein levels to reduce the threshold of activation of the heat shock response, and HPK-1 promotes longevity through modulation of HSF-1 activity under normal growth conditions.

Most neurodegenerative diseases are characterized by an age-dependent onset, which share the common molecular feature of abnormal protein aggregation leading to neuronal cell death. A number of studies have linked loss of either HSF1 or autophagy to the promotion of neurodegenerative diseases[104, 105]. We have discovered that HPK-1 functions as a central regulatory node linking these two complementary proteostatic mechanisms that act to preserve the proteome (Fig 15), and justifies consideration of HPK-1 as an attractive intervention point for the improvement of healthspan and protection against proteostatic diseases.

### Experimental procedures

#### *C*. *elegans* strains and maintenance, RNAi, and nematode culture

Strains were maintained at 20°C on standard NGM plates with OP50. Strains and genotypes used in this study are:

- N2 wild type (CGCM)
- AVS392: *hpk-1(pk1393)* (received 6X backcrossed from CGC and backcrossed 5 additional times)
- AVS84/TJ375: *gpIs1[Phsp-16*.*2*::*GFP]*
- AVS85/AM140: *rmIs132[Punc-54*::*Q35*::*YFP]*
- AVS253/DA2123: *adIs2122[lgg-1*::*GFP + pRF4 (rol-6(su1006))]*
- AVS393-394: *artEx11-12[Phpk-1*::*GFP+ pRF4(rol-6(su1006))]* (two independent lines)
- AVS181/KWN147: *pha-1(e2123ts) III; rde-1(ne219) V; rnyEx078[Pnhx-2*::*RDE-1; Pnhx-2*::*pHluorin; pCL1(pha-1+)]* (intestinal RNAi strain)
- AVS206/NR222: *rde-1(ne219) V; kzIs9 [pKK1260(lin-26p*::*nls*::*GFP) + pKK1253(lin-26p*::*rde-1) + pRF6(rol-6(su1006)]* (hypodermal RNAi strain)
- AVS454/NR350: *rde-1(ne219) V; kzIs20[pDM#715(hlh-1p*::*rde-1) + pTG95(sur-5p*::*NLS*::*GFP)]* (muscle RNAi strain)
- AVS216/NL3321: *sid-1(pk3321) V*
- AVS265/TU3401: *sid-1(pk3321) V; uIs69[punc-119*::*sid-1;pmyo-2*::*mCherry] V*.
- AVS309, 310 and 396: *artEx26-28[Phpk-1*::*hpk-1*::*GFP+ pRF4(rol-6 (su1006))]* (Three independent lines)
- AVS413: *hpk-1(pk1393); artEx29[Phpk-1*::*hpk-1*::*GFP + pRF4(rol-6 (su1006))]*
- AVS411: *artEx30[Phpk-1*::*hpk-1*::*tdtomato + pAH71(Phsf-1*::*hsf-1*::*GFP) + pRF4(rol-6(su1006)]*
- AVS408, 409: *artEx31-32[Psur-5*::*hpk-1*::*CFP* + *pCFJ90*(*Pmyo-2*::*m-cherry*)*]*
- AVS397, 398: *gpIs1[Phsp-16*.*2*::*GFP]*; *artEx35-36[Psur-5*::*hpk-1*::*CFP + pCFJ90(Pmyo-2*::*m-cherry)]* (two independent lines)
- AVS401/AGD710: *uthIs235[Psur-5p*::*hsf-1 FL*, *+ Pmyo-2*::*tdTomato]*
- AVS403, 404: *rmIs132[unc-54p*::*Q35*::*YFP]; artEx37-38[Psur-5*::*hpk-1*::*CFP* + *pCFJ90 (Pmyo-2*::*m-cherry)]* (two independent lines)
- AVS438/EQ87: *[Phsf-1*::*hsf-1*::*GFP + pRF4(rol-6(su1006))]*
- AVS405/OG497: *drSi13[hsf-1p*::*hsf-1*::*GFP*::*unc-54 3'UTR + Cbr-unc-119(+)]*
- AVS469/TJ356: *zIs356[daf-16p*::*daf-16a/b*::*GFP pRF4(rol-6(su1006))]*
- AVS474/MAH240: *sqIs17[hlh-30p*::*hlh-30*::*GFP + pRF4(rol-6(su1006))]*
- AVS464: *hpk-1(pk1393); rmIs132[Punc-54*::*Q35*::*YFP]*
- AVS471: *rmIs132[Punc-54*::*Q35*::*YFP]; artEx32[Psur-5*::*hpk-1*::*CFP* + *pCFJ90 (Pmyo-2*::*m-cherry)]*

Multiple strain designations per genotype indicate independently generated isolates of identical genotype. In cases where both an AVS and an outside laboratory designation are given, the latter indicates the source strain we received and the former our in-house laboratory designation.

### RNAi

RNAi clones originated from early copies of *E*. *coli* glycerol stocks of the comprehensive RNAi libraries generated in the Ahringer and Vidal laboratories. Each RNAi colony was grown overnight in Luria broth with 50 μg ml–1 ampicillin and then seeded onto 24-well RNAi agar plates containing 5 mM isopropylthiogalactoside to induce dsRNA expression overnight at room temperature. All of the RNAi clones used in this study were verified by DNA sequencing and subsequent BLAST analysis to confirm their identity.

### Generation of transgenic strains

Fusion of promoter and gene sequences to reporters were carried about using the SOEing technique [106]. Pooled PCR products were microinjected to generate transgenic lines as follows.

*Phpk-1*::*GFP*: 2kb upstream of ATG start for the longest isoform of *hpk-1* was amplified from fosmid WRM066aH12 and fused to GFP (from pPD95.75) (Addgene) using the following primers:

*Phpk-1* Primer A *5’- AATTTTCAAGATAGGGCCGCCG*
*Phpk-1* Primer B (nested to GFP) *5’-AGTCGACCTGCAGGCATGCAAGCTCGCCACCCAATCAAACAATCG*
*Phpk-1* Primer C *5’- AGCTTGCATGCCTGCAGGTCG*
*Phpk-1* Primer D *5’- AAGGGCCCGTACGGCCGACTA*
*Phpk-1* Primer A’- *5*’-*CCTCCTTCGCCAAGTTTCGA-3’*GFP
GFP Primer D* (nested)- *5’- GGAAACAGTTATGTTTGGTATA-3’*

30ng of fused PCR product was injected with 120ng of pRF4 (*rol-6*) as a coinjection marker.

*Phpk-1*::*hpk-1*::*GFP*: C-terminal fusion of full-length *hpk-1* to *GFP* was generated using the fosmid template WRM066aH12 and pPD95.75 as above with following primers:

*hpk-1* Primer A *5’- AATTTTCAAGATAGGGCCGCCG*
*hpk-1* Primer B nested to GFP with mutated STOP codon *5’-AGTCGACCTGCAGGCATGCAAGCTTATGCGAGTGCAATGAAGTGATGAGT*
*hpk-1* Primer A’ *5*’-*CCTCCTTCGCCAAGTTTCGA*
GFP Primer D* (nested) *5’-GGAAACAGTTATGTTTGGTATA*

5ng of PCR product was injected into wild type and *hpk-1*(*pk1393*) animals with 150ng of pRF4 as coinjection marker.

*Phpk-1*::*hpk-1*::*tdtomato* C-terminal fusion of full-length *hpk-1* to *GFP* was generated using the fosmid WRM066aH12 and pDS9 (tdTomato) as templates

*hpk-1* Primer A *5’- AATTTTCAAGATAGGGCCGCCG -3’*
*hpk-1* PrimerB nested to tdtomato 5’-*AGTCGACCTGCAGGCATGCAAGCTTTATGCGAGTGCAATGAAGTGATGAGT-3’*
Primer C tdTomato *5’-AAGCTTGCATGCCTGCAGGTCG-3’*
Primer D tdTomato *5’-GGAAACAGTTATGTTTGGTATA-3’*

For visualizing co-localization of HSF-1::GFP with HPK-1::tdTomato, pAH71 (*P-hsf-1*::*hsf-1*::*GFP*) was obtained from Dr. Hsu (University of Michigan, Ann Arbor) and injected at a concentration of 15ng/ul either with 5ng/ul or 30ng/ul of *P-hpk-1*::*hpk-1*::*tdtomato*.

*Psur-5*::*hpk-1*::*CFP*: 1.5kb promoter region of *sur-5* amplified from genomic DNA was fused to *hpk-1* genomic fragment and *CFP* at the C-terminus in a nested PCR reaction. *CFP* was amplified from pPD134.96.

*Psur-5 PrimerA cattcgggctggaaatctgaatg*
*Psur-5 PrimerA' gagtcccaccatgcttgtcatc*
*Psur-5*:: *hpk1 PrimerB CGGAGTTCTTACGCTTTGGCATgcgatatcaccacttctaggcgt*
*Primer D CFP CTTTTGGGCCCAAGCGAGG*

5ng/ul of the PCR fusion construct, 5ng/ul of *Pmyo-2*::*m-cherry* (pCFJ90), and Log2 DNA ladder (NEB N3200S) was included as carrier DNA.

### Lifespan assays

Lifespan assays were performed essentially as described [107]. Synchronized L1 animals were seeded onto plates and allowed to develop to L4 stage at 20°C, with two exceptions: for the hormesis experiments (S5G and S5H Fig) animals were shifted to 25°C at L4 and maintained at this elevated temperature until day 3 of adulthood and then shifted back to 20°C; and for the intestinal specific RNAi (S2D Fig) animals were maintained at 25°C during development (this strain is a *pha-1* rescue strain that must be maintained at 25°C during development to ensure maintenance of the extrachromosomal transgene[108]). In all cases, at L4 2’ fluoro– 5’deoxyuridine (FUDR) was added to a final concentration of 400 uM to prevent progeny production (defined as day 0 adulthood). Viability was scored every day or every other day as indicated in each figure. Survival analysis was performed for all the experiments using the Kaplan-Meier estimator and Peto & Peto’s generalized Wilcoxon test, both as implemented in the R package *survival* version 2.38 (http://CRAN.R-project.org/package=survival). P-values were corrected for multiple testing with the Benjamini-Hochberg FDR approach[109].

To determine temporal requirements in longevity control (S2A–S2C Fig), synchronized L1 animals were seeded on control or *hpk-1* RNAi according to the following conditions: for constitutive RNAi L1 animals exposed to each of the RNAi conditions throughout lifespan, for developmental gene inactivation animals were fed *E*. *coli* expressing dsRNA to *hpk-1* or control (empty vector) from L1-L4 after which animals were moved to *dcr-1* (*dicer-1*) RNAi, and for adult inactivation animals were maintained on control RNAi plates until L4 and then moved to either control, *hsf-1* or *hpk-1* RNAi plates.

Detailed information on all lifespan trials, including number of animals scored can be found in S1 Table.

### Thermotolerance assays

Time course survival assays at high temperature were conducted using the replica set method as previously described [72]. In brief, synchronized L1 animals were allowed to develop at 20°C; FUDR was added at the L4 stage and at day three adulthood animals were moved to 35°C for the indicated time period, allowed to recover for 1–2 hours at 20°C, and viability was scored every 2 hours for up to 14 hours. Significance of pairwise comparisons between genotypes was determined by fitting the data to logit curves with pooled data from all three trials and randomized permutation testing with 100,000 iterations to determine p-values, as previously reported in [38, 72]. For single time point thermotolerance assays, synchronized day 1 adult animals were heat shocked at 35°C for 9–10 hours and viability was scored the next day. 3–4 technical replicates were included in each of two independent trials. Statistical testing for single time point assays was performed with ANOVA followed by Tukey’s HSD post-hoc test, and p-values were corrected to account for multiple testing.

### Fluorescent analysis of transgenic lines

#### Induction of *Phpk-1*::*GFP* and *Phpk-1*::*hpk-1*::*GFP* after heat shock

To assess changes *of hpk-*1 expression after heat shock, late L4 transgenic animals expressing either the transcriptional (*Phpk-1*::*GFP*) or translational (*Phpk-1*::*hpk-1*::*GFP*) reporter were heat shocked at 37°C for 1 hour and allowed to recover for at least 15 minutes. Animals were then immobilized on 2% agarose pads with 1mM tetramisole hydrochloride and imaged using Leica SP5 confocal microscope at 40X magnification. Z-stack imaging was performed and 3-D projection was used to create composite images. Three independent trials were performed with a minimum of 20 animals per condition.

#### Induction of *Phpk-1*::*hpk-1*::*GFP* in response to metabolic stress

To assess changes of *hpk-1* expression after altered metabolic signaling, L1 *Phpk-1*::*hpk-1*::*GFP* transgenic animals were seeded on control, *daf-15*, *rictor-1* or *daf-2* RNAi. 400 μM of FUDR was added at the L4 stage and animals were visualized on day 4 of adulthood using confocal microscope Leica SP5. Z-stack imaging was performed for each image that was combined using 3D projection on Leica software LAS to give a composite image. At least three independent trials visualizing a minimum of 15–20 animals each were performed.

#### Induction of *Phpk-1*::*hpk-1*::*GFP* after UV and oxidative stress

UV damage was induced in late L4 transgenic animals by exposure to 300 μjoules (Stratalinker). Oxidative stress was induced by 1hr of exposure to 7.5mM tert-butyl hydrogen peroxide (as in [89]). Animals were allowed to recover for at least fifteen minutes prior to imaging on confocal microscope Leica SP5 at 40X. At least 15–20 animals were visualized for each condition.

#### Induction of *Phsp-16*.*2*::*GFP* after heat shock

The *Phsp-16*.*2*::*GFP* animals imaged in Fig 7A–7D were heat shocked on day 1 of adulthood, for 3 hours at 35°C and imaged after 3 hours of recovery. *Phsp-16*.*2*::*GFP* transgenic animals imaged in Fig 8A–8D were heat shocked at 35°C for 1 hour on day 3 of adulthood, allowed to recover for 7–8 hrs and imaged using a Zeiss Axio Imager M2m using QCapturePro v6.0 software. Four independent trials with a minimum of 30 animals per experiment were performed.

#### Measurement of autophagosome formation using the *lgg-1*::*GFP* reporter

LGG-1::GFP foci formation was visualized in L3 stage animals that were raised on either control (EV), *hsf-1* or *hpk-1* RNAi from the preceding parental generation and imaged using confocal Leica software at 63X magnification. 15–20 animals were scored for seam cell LGG-1::GFP puncta accumulation after 6 hrs of bacterial deprivation (BD) or *ad libitum* (AL).

#### Measurement of HLH-30::GFP and DAF-16::GFP subcellular localization

HLH-30::GFP and DAF-16::GFP transgenic animals were treated with empty vector or *hpk-1(RNAi)* starting at the L1 stage, FUDR was added at L4, and at day 3 of adulthood animals were either subjected to bacterial deprivation or allowed to continue to feed *ad libitum* for 24 hours. For each condition, immediately prior to visualization 10 animals were moved to a fresh plate with sodium azide to prevent movement. GFP localization was then imaged on a Zeiss Axio Imager M2m using QCapturePro v6.0 software. At least 30 animals were visualized for each condition.

### Polyglutamine aggregation and locomotory analyses

Synchronized *Punc-54*::*polyQ35*::*YFP* L1 progeny were treated with the indicated RNAi and FUDR was added at L4 stage to prevent progeny production. To quantify fluorescent foci formation, between 15–50 animals per technical replicate were scored blind daily from days one to three of adulthood. To assess paralysis, prodded animals that responded with head movement (and were therefore still alive) but no backward or repulsive motion were scored as paralyzed (as described in [72]). Statistical testing between pairs of conditions for differences in paralysis rates was performed with Cox modeling and the Wald test.

### Generation of HSF-1 antibodies

Rabbit polyclonal antibodies to *C*. *elegans* HSF-1 were generated using purified, recombinant *C*. *elegans* HSF-1, which was recovered from *E*. *coli* expressing His6-HSF-1 from a modified version of the pET-28a(+) vector by affinity purification and subsequent on column thrombin cleavage to remove the His6 tag. Polyclonal rabbit antibodies to the full-length, untagged protein were generated by Covance (http://www.covance.com/services/lead-optimization/immunology/custom-antibody-development.html) through injection of two rabbits, of which the one with the least background immunogenicity was chosen.

### Immunoblotting

Approximately 1000 synchronized animals were collected with M9, washed, and pelleted animals were snap frozen in liquid nitrogen. Pellets were lysed in RIPA buffer (150 mM NaCl, 1.0% NP-40, 0.5% sodium deoxycholate, 0.1% SDS, 50 mM Tris (pH 8.0)) with 40 mM N-Ethylmaleimide (NEM, Thermo #PI23030); per 1 mL RIPA, 40 μL of 100x Halt Protease & Phosphatase Inhibitor (Thermo #78446), and 40 μL 100x EDTA (0.5 M) was added. Briefly, extracts were vortexed with Zirconin 2.0 mm beads (#11079124ZX, BioSpec) and lysate was separated from pellet by centrifugation. Samples were normalized using a Bradford assay and was resolved by 6% SDS-PAGE. The antibodies used to probe membranes of the immunoblots were anti-HSF-1 (1:500), and anti-beta-actin (1:1000) (Cell Signaling #4967).

The ratio of HSF-1 unmodified (75kD) to modified (90-95kD, sumoylated and sumoylated plus phosphorylated) was determined based on the quantification of band intensity in Image J. Protein levels of unmodified (75kD), modified (90-95kD) HSF-1, and beta-actin were also quantified in ImageJ from western blots. HSF-1 expression was first normalized to matched beta-actin levels and then relative fold-change compared to N2 was calculated. The S.E.M. from quantified extracts/western blots was calculated and in both cases significance was determined by Student’s t-test.

For detection of HSP-16.2 protein and GFP reporter expression on RNAi, 100 worms grown at 16°C on RNAi were collected in 25 μL dH2O. An equal volume of 2x SDS-PAGE loading dye was added followed by 15 min boiling. A 10–20% Tris-HCl gel (Bio-Rad) was loaded with 15μL (~30 worms) after another 15 min boiling, run, transferred to nitrocellulose, and blocked in 2% milk in 1X TTBS (1M Tris, 150 mM NaCl, 0.1%Tween 20). Membrane was probed simultaneously for HSP-16.2 (1:5000, rabbit, #5506 R120; kind gift of Chris Link, UC Boulder), GFP (1:1000, mouse, Roche 7.1 and 13.1) and β-actin (1:2000, mouse, Sigma AC-15) at 4°C overnight. Blots were washed in 0.1% milk 1X TTBS three times. Secondary antibodies were anti-mouse HRP and anti-rabbit HRP (1:5882 dilution both, Amersham). Blots were visualized with Amersham ECL Western Blotting System (RPN2108).

### Immunoprecipitation

For HSF-1 immunoprecipitations, L4 *C*. *elegans* pellets were collected as per sample collection for immunoblotting. *C*. *elegans* pellets were lysed in 20 mM Tris-HCl (pH 8.0), 170 mM KCl, 0.5% NP-40 (IGEPAL CA-360), 2 mM EDTA, with 40 μL of 100x Halt Protease & Phosphatase Inhibitor, 40 μL 100x EDTA (0.5 M) (Thermo #78446) per mL of lysis buffer. Lysates were prepared and quantified as above, and then 1.5 mg of lysate was precleared by the addition of 10 μL of normal rabbit serum (Invitrogen # 016101) and incubated with rotation for 1 h at 4°C, at which time the equivalent of 120 μL of drained Protein-A-Sepharose was added to immunoprecipitate non-specific interactions and incubation continued an additional 2 h. The proteins were eluted from the nonspecific pellet fraction with the addition of equal volume 2x SDS reducing buffer (1x SDS reducing buffer: 62.5 mM Tris (pH 6.8), 10% glycerol, 0.02% SDS, and 5% β-mercaptoethanol) and boiled for 5 min. Precleared lysate was evenly divided, antibodies were added, and lysates were incubated with rotation for 2 h at 4°C. The antibodies used in various immunoprecipitations were as follows: for HSF-1 1 μL of HSF-1 immune serum, and for negative controls either 1 μL of commercially available NRS (Invitrogen # 016101) or pre-immune serum from the rabbit prior to injection with recombinant *C*. *elegans* recombinant HSF-1. The equivalent of 50 μL of drained beads of Protein A-Sepharose was added, and lysates were incubated with rotation for an additional 2 h at 4°C. Immunoprecipitates were washed three times with lysis buffer, resuspended in 2x SDS reducing buffer, boiled for 5 min, and resolved by 6% SDS-PAGE. Subsequent immunoblotting for HSF-1 was carried out as described above.

### SUMO protease treatment of immunoprecipitation

Prior to elution after immunoprecipitation, beads of Protein A-Sepharose were washed three times with 1x SUMO Protease Buffer (50 mM Tris-HCl (pH8.0), 0.2% Igepal (NP-40), 150 mM NaCl, 1 mM DTT), and resuspended in 100 μL of 1x SUMO protease buffer with or without 5 μL of SUMO protease (ULP1; Invitrogen #12588–018). Reactions were performed at 20°C for 1 hour with nutating. Immunoprecipitate was eluted with the addition of equal volume 2x SDS reducing buffer and boiled for 5 min.

### Phosphatase treatment of protein extracts

Extracts were prepared as above but with HALT complete protease inhibitor cocktail (Thermo #87785) without EDTA. Protein dephosphorylation was carried out with lambda Protein Phosphatase (λPP, New England Biolabs #P0753S) treatment. For λPP treatment 25 μg total protein was incubated for 1 h at 30°C in 1x NEBuffer for MetalloPhosphatases (PMP), 1 mM MnCl_2_, and 400U of λPP. As a negative control mock λPP treatments used enzyme that had been inactivated by 10 min of boiling prior to addition and samples contained Halt Protease & Phosphatase Inhibitor (Thermo #78446). In all cases, after 1 h of incubation SDS reducing buffer was added and samples were boiled 5 minutes.

### Quantitative real time PCR

For measurement of *hsp* gene induction in response to heat shock, late L4 animals were heat shocked at 37°C for one hour and allowed to recover for two hours before harvesting in M9 buffer. For measurement of *atg-18*, *bec-1*, *ifg-1*, *iftb-1*, *hsp-16*.*2*, and *hsp-70* mRNA levels after *daf-15* inactivation, synchronized populations were grown until L4 at 20°C on RNAi plates and animals were harvested on day 1 of adulthood. RNA was isolated using Trizol reagent (Life Technologies). RNA concentration was measured using a Nanodrop, and RNA preparations were reverse transcribed into cDNA using the BioRad cDNA synthesis kit (#1708890) as per manufacturer’s protocol. For all qRT-PCR primer pairs, (-) RT test reactions were run to confirm mRNA purity and target specificity. Quantitative real time PCR was performed using SYBR green supermix (Biorad) with three biological and two technical replicates for each condition. Primer sets with at least one primer spanning the exon were used to amplify the gene of interest. *cdc-42* mRNA levels were used for normalization of autophagy and translation gene expression. *act-1* mRNA levels were used for normalization of *hsp* gene expression. Fold change in mRNA levels was determined using ΔΔ Ct method [110]. Primer sequences used were as follows:

*act-1*F 5’-CCATCATGAAGTGCGACATTG
*act-1*R 5’-CATGGTTGATGGGGCAAGAG
*hsp-16*.*2*F 5’-CTCCATCTGAGTCTTCTGAGATTGT
*hsp-16*.*2*R 5’-CTCCTTGGATTGATAGCGTACGA
*hsp-70*F 5’-GATGAAGTTGTCTTGGTTGG
*hsp-70*R 5’-CAGTTGAGGTCCTTCCCATTG
*atg-18*F 5’-ACTTGAGAAAACGGAAGGTGTT
*atg-18*R 5’-TGATAGCATCGAACCATCCA
*cdc-42*F 5’-AGCCATTCTGGCCGCTCTCG
*cdc-42*R 5’-GCAACCGCTTCTCGTTTGGC
*bec-1*F 5’-TTTTGTTGAAAGAGCTCAAGGA
*bec-1*R 5’-’CAACCAGTGAATCAGCATGAA
*ifg-1*F 5’-GCTAGCTGATTTCGGATTGG
*ifg-1*R 5’-CACGGTATGGAACCTGCTG
*iftb-1*F 5’-ATGGCACATTACGTACTCCTTG
*iftb-1*R 5’-CGGAAGTAACTGTTTGATTGTGAA
*hpk-1*F 5’-AGTATGCACAGCTCCATCAC
*hpk-1*R 5’-CCATTATTGGGACCGGAACA

### Ethics statement

This study did not employ Human Subject Research or Animal Research that would require IACUC oversight or approval.

## Supporting information

S1 Fig HPK-1 spatial expression during development and subcellular localization. (A) **HPK-1 is broadly expressed during development, with limited expression by adulthood**. (A, Top left) Expression of *Phpk-1*::*HPK-1*::*GFP* during embryogenesis. (A, top right) *Phpk-1*::*HPK-1*::*GFP* expression becomes increasingly restricted by the L3 stage of development to neurons (arrow with N), intestinal cells (arrow with I), and the hypodermis (arrow with H). (A bottom panel) HPK-1 expression is limited to neuronal cells by the L4 stage of development and in adults (not shown). Brightfield insets for each image and white dotted lines trace animals. In all cases, representative images are shown of at least fifteen animals from two independently derived lines. (B) HPK-1 is localized in the nucleus. *Phpk-1*::*HPK-1*::*GFP* expression with the overlay of DIC image. Arrows indicate examples of localized expression within nuclei.
(TIF)

S2 Fig HPK-1 temporal requirements in longevity control parallel *hpk-1* expression. (A) Constitutive inactivation of *hpk-1* (red) from the L1 stage by feeding-based RNAi in wild-type N2 worms decreases lifespan compared to control (EV) (black) RNAi. (B) *hpk-1* RNAi only during development (red) results in a decrease in lifespan comparable to constitutive *hpk-1* RNAi. Animals were moved to *dcr-1(RNAi)* at L4. (C) *hpk-1* RNAi (red) initiated at L4 decreases lifespan to a smaller, but significant extent. **HPK-1 spatial requirements mirror tissues of expression**. (D) Intestinal or (E) hypodermal specific gene inactivation of *hpk-1* (red) reduces lifespan. In contrast, (F) muscle specific gene inactivation of *hpk-1* has a minimal effect on lifespan, consistent with a lack of muscle expression during larval development (S1 File). (G) Neuronal inactivation of *hpk-1* (pink) reduces lifespan. (H) Neuronal inactivation of *daf-2* increases lifespan (orange), consistent with previous reports[47, 48]. (I) *sid-1(pk3321)* loss of function animals have impaired RNAi: *hpk-1(RNAi)* (pink) and *daf-2(RNAi)*-treatment (red), respectively. Full lifespan data can be found in S1 Table.
(TIF)

S3 Fig HPK-1 prevents sumoylation of HSF-1. (A) Inactivation of the SUMO moiety *smo-1* by RNAi prevents formation of higher molecular weight isoforms of HSF-1. Data supports Fig 6A. RNAi knockdown of *smo-1* showed some variability in the degree of effectiveness, but generally decreased the relative proportion of higher molecular weight isoforms of HSF-1. Relative proportion of modified HSF-1 shown in the lower panel is 0.49, 0.35, and 0.77 for N2/ev, *hpk-1(pk1393)/smo-1*, and *hpk-1(pk1393)/ev*, respectively. ev = empty vector RNAi treatment (L4440). dePhos is λ protein phosphatase treatment. β-actin serves as a loading control. (B, C) Additional replicates for quantification of the ratio of modified to unmodified HSF-1 are shown in Fig 6B. For (B) no β-actin control was available, which precluded quantifying differences in overall expression levels between samples. (C) Additional replicates for quantifying differences in overall HSF-1 levels between N2 and *hpk-1(pk1393)* shown in Fig 6C. β-actin serves as a loading control. In all cases U = unmodified (75 kD), S = sumoylated (~90 kD), and S+P = sumoylated and phosphorylated (~95 kD) isoforms of HSF-1.
(TIF)

S4 Fig Mild heat stress at 25°C hormetically increases lifespan and induces mosaic expression of *hsp-16*.*2*. (A, B) *Phsp-16*.*2*::*GFP* is not expressed at 20°C (basal conditions) in day one adult animals. Outlines of animals are traced in white. (C, D, E, F) *Phsp-16*.*2*::*GFP* is induced heterogeneously across tissues after continued exposure to mild temperature stress of 25°C in day one adult animals. (G, H) Transient hormetic exposure to 25°C increases mean lifespan (gray versus black), which is dependent on both *hpk-1* (G, red) and *hsf-1* (H, blue). See S1 Table for comprehensive lifespan data.
(TIF)

S5 Fig HSF-1::GFP stress granule formation and nuclear accumulation after heat shock occurs independently of *hpk-1*. (A) Strain expressing a single copy of HSF-1::GFP is diffusely localized in the nucleus under basal conditions. (B) Strain expressing a single copy of HSF-1::GFP forms nuclear stress granules after heat shock. (C-D) Strain expressing a single copy of HSF-1::GFP treated with *hpk-1(RNAi)* does not alter HSF-1 basal localization (C) or formation of stress granules after heat stress (D). Results were consistent across three independent trials; approximately 20 animals were visualized per trial and all animals produced nuclear stress granules regardless of *hpk-1* status. (E) Strain with low copy HSF-1::GFP shows increased nuclear accumulation after heat stress (column 1 to 2), which is not altered in the absence of *hpk-1* (compare 3 to 4). Two independent trials produced similar results, with 10 animals per condition in each trial. Graph includes results from one trial (*** p <0.001, Student’s t-test).
(TIF)

S6 Fig HPK-1 is induced in head neurons and hypodermal seam cells following heat shock. (A) Mean GFP intensity was measured in ImageJ for: head neurons, 2–4 hypodermal seam cells, and 4 spots of internal non-neuronal, non-seam cell background fluorescence for the indicated number of animals (n). Head and seam cell GFP intensity was normalized to average background fluorescence, and the contribution of background fluorescence was subtracted. Mean and standard deviation of head neuron and seam cell fluorescence in unstressed and heat shocked animals was calculated, and normalized to unstressed animals. * indicates p<0.0005, ** p<0.0002 (Student’s t-test). (B) As an independent verification of increased fluorescence within head neurons, we applied a matrix for assessing GFP levels via double-blind visualization, which allowed us to score a larger cohort of animals +/- heat shock. GFP expression was scored as “strong”, “moderate”, or “weak”. * p<0.03 (Mann-Whitney).
(TIF)

S7 Fig *hpk-1* mRNA expression does not increase after acute heat shock. (A-B). *Phpk-1*::*GFP* expression in L4 animals under unstressed conditions (A) or after 1 hour heat shock (B). For additional images see S1 File. (C) Endogenous *hpk-1* mRNA levels of L4 wild-type unstressed animals or following a 15-minute recovery after 1 hour of heat shock at 35°C. (D) Endogenous *hsp-16*.*2* mRNA levels are induced following a 15-minute recovery after 1 hour of heat shock (p<0.05, Student’s t-test). Fold change is relative to unstressed N2. Note: mRNA from the same extracts was used in (C) and (D). In all cases expression is normalized to *act-1* mRNA and error bars represent the standard deviation of a minimum of three replicates in each of two independent trials.
(TIF)

S8 Fig α-amanitin treatment suppresses *Phsp-16*.*2*::*GFP* transcriptional reporter after heat shock. (A-C) Expression of *Phsp-16*.*2*::*GFP* animals under the following conditions: unstressed (A), 2 hours of recovery following heat shock for 1 hour at 35°C heat shock (B), and heat shock as in (B) but with prior treatment with 100 ug/mL α-amanitin treatment (C) (as described in [112]). Note, images in (B) and (C) were taken at a similar magnification, which was higher than image (A). Outlines of animals are traced in white.
(TIF)

S9 Fig Decreased TORC1 increases HPK-1 in neurons. (A) Mean GFP intensity within head neurons was measured in ImageJ for *Phpk-1*::*HPK-1*::*GFP* expressing animals after empty vector or *daf-15(RNAi)*-treatment. Change in expression was normalized to empty vector; error bars represent the standard deviation of six animals. * p<0.03 (Student’s t-test). (B-D) Additional images of *Phpk-1*::*HPK-1*::*GFP* expression after empty vector control or *daf-15(RNAi)* treatment. Expression of *Phpk-1*::*HPK-1*::*GFP* is only induced within neurons after *daf-15* (Raptor) inactivation compared to control. While expression within hypodermal seam cells varied between animals, no differences in expression between empty vector and *daf-15* RNAi could be discerned, which is in contrast to heat shock. Images in (C) and (D) were stitched together from several overlapping high-resolution images and white space was artificially filled.
(TIF)

S10 Fig *hpk-1* is not required for HLH-30::GFP nuclear localization following bacterial deprivation. *Phlh-30*::*HLH-30*::*GFP* animals were treated with either empty vector (A, C) or *hpk-1(RNAi)* (B, D) and then were placed under *ad libitum* or bacterial deprivation conditions for 24 hours and HLH-30::GFP subcellular localization was assessed.
(TIF)

S11 Fig *hpk-1* is not required for DAF-16::GFP nuclear localization following bacterial deprivation. *Pdaf-16*::*DAF-16*::*GFP* animals were treated with either empty vector (A, C) or *hpk-1(RNAi)* (B, D) and then were placed under *ad libitum* or bacterial deprivation conditions for 24 hours and DAF-16::GFP subcellular localization was assessed.
(TIF)

S1 Table Complete lifespan data and select statistical analyses. This dataset contains all lifespan data discussed within this manuscript, as well as data from other experiments that support lifespan data represented in in-text and supplemental figures. This dataset also contains select statistical analyses to support the significance of the lifespan differences described in the text.
(XLSX)

S2 Table Complete thermotolerance data. This dataset contains all data pertaining to Fig 5: including median lifespan, p-values, and total number of animals examined for thermotolerance assays.
(XLSX)

S3 Table Complete proteostasis data. This dataset contains all data pertaining to Figs 2D and 2E, 3D and 3E and 13: including: total number of animals examined for polyQ foci formation and paralysis, quantification, and statistical analysis. Supporting experiments with additional independently derived transgenic lines are also reported.
(XLSX)

S4 Table Complete autophagosome formation data. This dataset contains all data pertaining to Fig 10: including total number of cells examined for autophagosome formation, average number of foci per cell, and S.E.M.
(XLS)

S5 Table Conserved HSF-1 amino acids that are known to undergo post-translational modifications in mammals. (XLSX)

S1 File Additional images of *Phpk-1*::*GFP* transgenic animals under an unstressed condition or after heat stress (S1 File). (PDF)
