# Supplementary figures and images for: The homeodomain-interacting protein kinase HPK-1 preserves protein homeostasis and longevity through master regulatory control of the HSF-1 chaperone network and TORC1-restricted autophagy in Caenorhabditis elegans
Source: PLoS Genet. 2017 Oct 16;13(10):e1007038. doi: 10.1371/journal.pgen.1007038 (PMC5658188; doi:10.1371/journal.pgen.1007038)

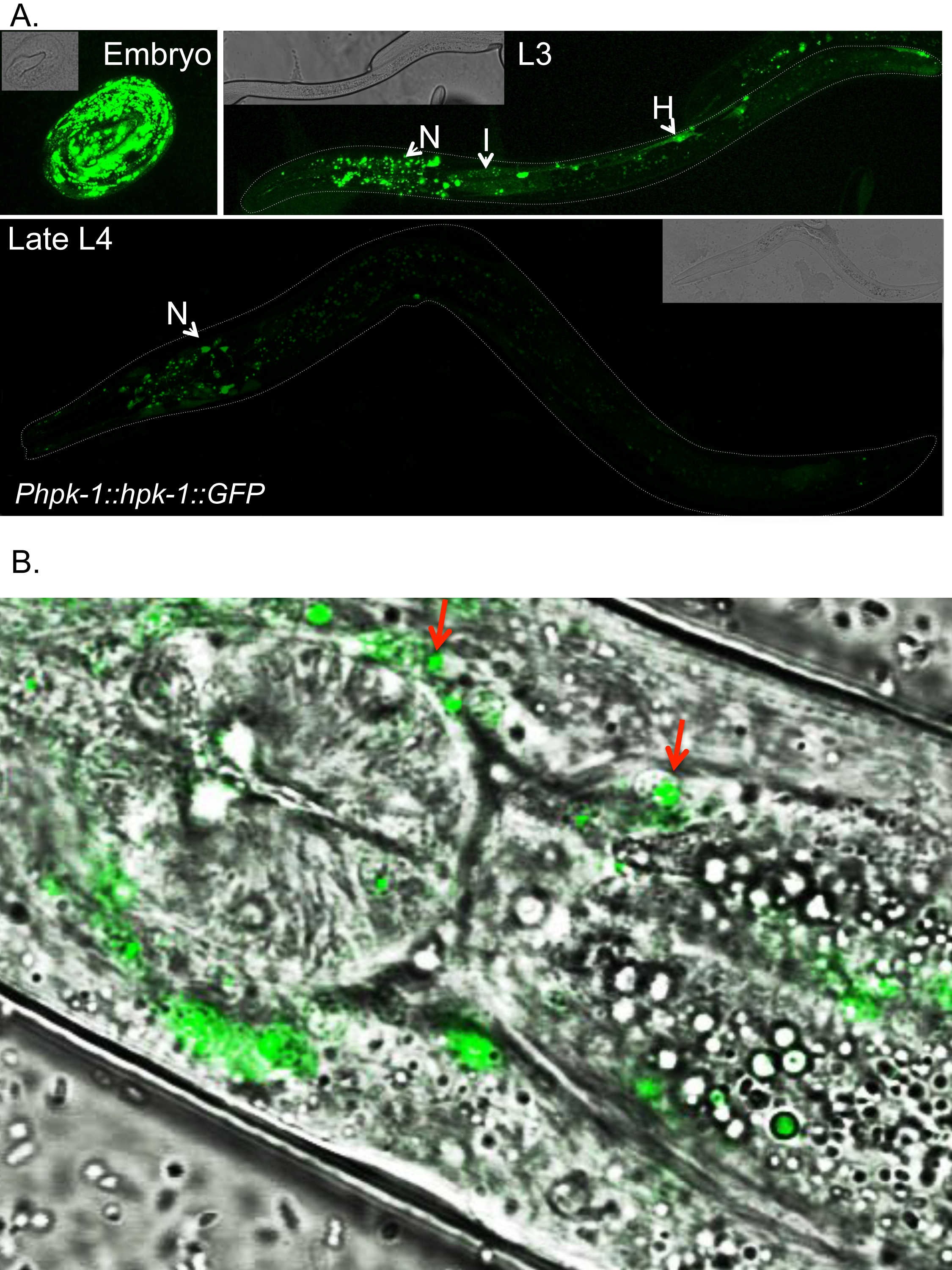

Supplement: S1 Fig — (A) HPK-1 is broadly expressed during development, with limited expression by adulthood. (A, Top left) Expression of Phpk-1::HPK-1::GFP during embryogenesis. (A, top right) Phpk-1::HPK-1::GFP expression becomes increasingly restricted by the L3 stage of development to neurons (arrow with N), intestinal cells (arrow with I), and the hypodermis (arrow with H). (A bottom panel) HPK-1 expression is limited to neuronal cells by the L4 stage of development and in adults (not shown). Brightfield insets for each image and white dotted lines trace animals. In all cases, representative images are shown of at least fifteen animals from two independently derived lines. (B) HPK-1 is localized in the nucleus. Phpk-1::HPK-1::GFP expression with the overlay of DIC image. Arrows indicate examples of localized expression within nuclei. (TIF) [file pgen.1007038.s001.tif]

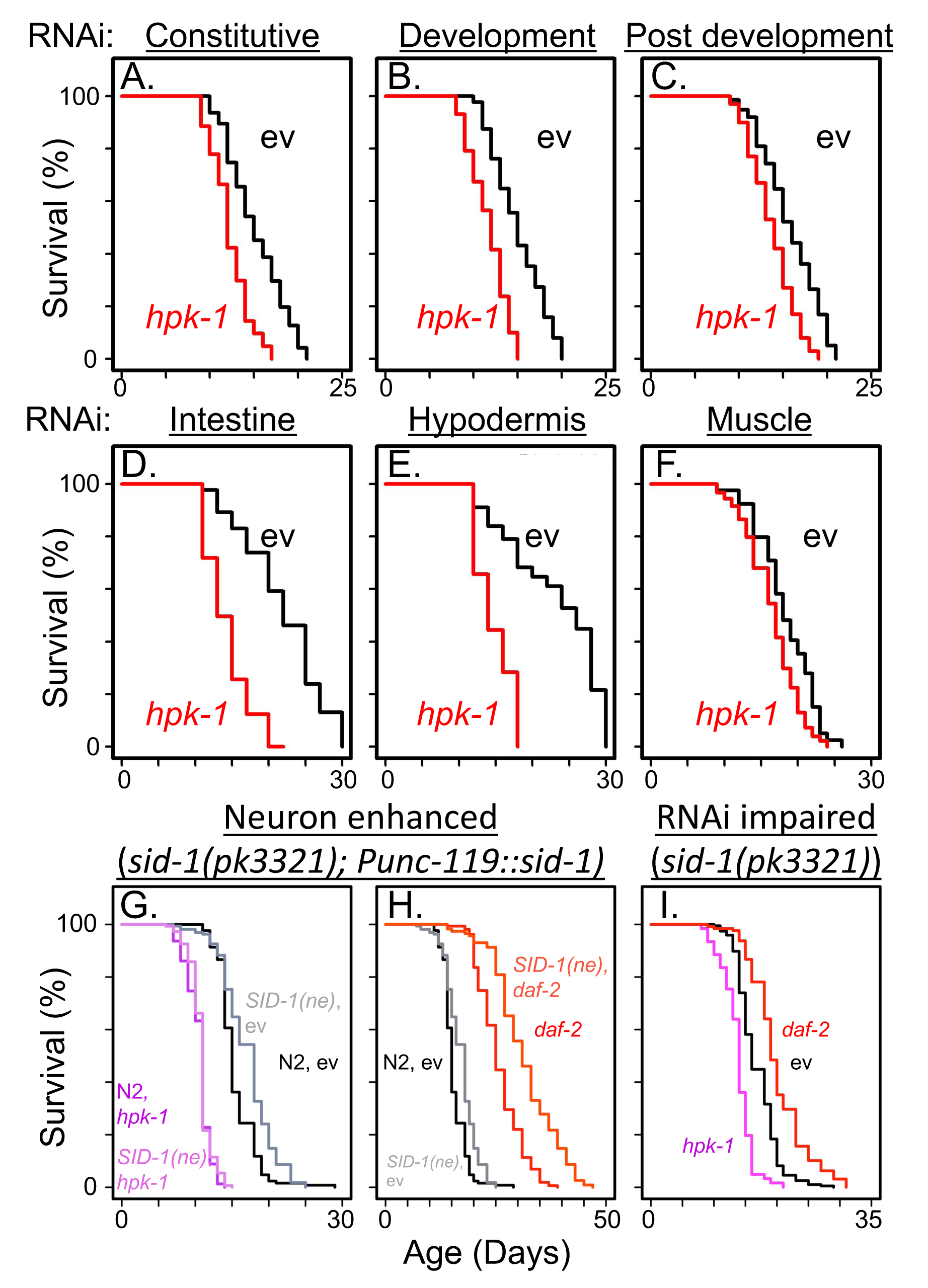

Supplement: S2 Fig — (A) Constitutive inactivation of hpk-1 (red) from the L1 stage by feeding-based RNAi in wild-type N2 worms decreases lifespan compared to control (EV) (black) RNAi. (B) hpk-1 RNAi only during development (red) results in a decrease in lifespan comparable to constitutive hpk-1 RNAi. Animals were moved to dcr-1(RNAi) at L4. (C) hpk-1 RNAi (red) initiated at L4 decreases lifespan to a smaller, but significant extent. HPK-1 spatial requirements mirror tissues of expression. (D) Intestinal or (E) hypodermal specific gene inactivation of hpk-1 (red) reduces lifespan. In contrast, (F) muscle specific gene inactivation of hpk-1 has a minimal effect on lifespan, consistent with a lack of muscle expression during larval development (S1 File). (G) Neuronal inactivation of hpk-1 (pink) reduces lifespan. (H) Neuronal inactivation of daf-2 increases lifespan (orange), consistent with previous reports[47, 48]. (I) sid-1(pk3321) loss of function animals have impaired RNAi: hpk-1(RNAi) (pink) and daf-2(RNAi)-treatment (red), respectively. Full lifespan data can be found in S1 Table. (TIF) [file pgen.1007038.s002.tif]

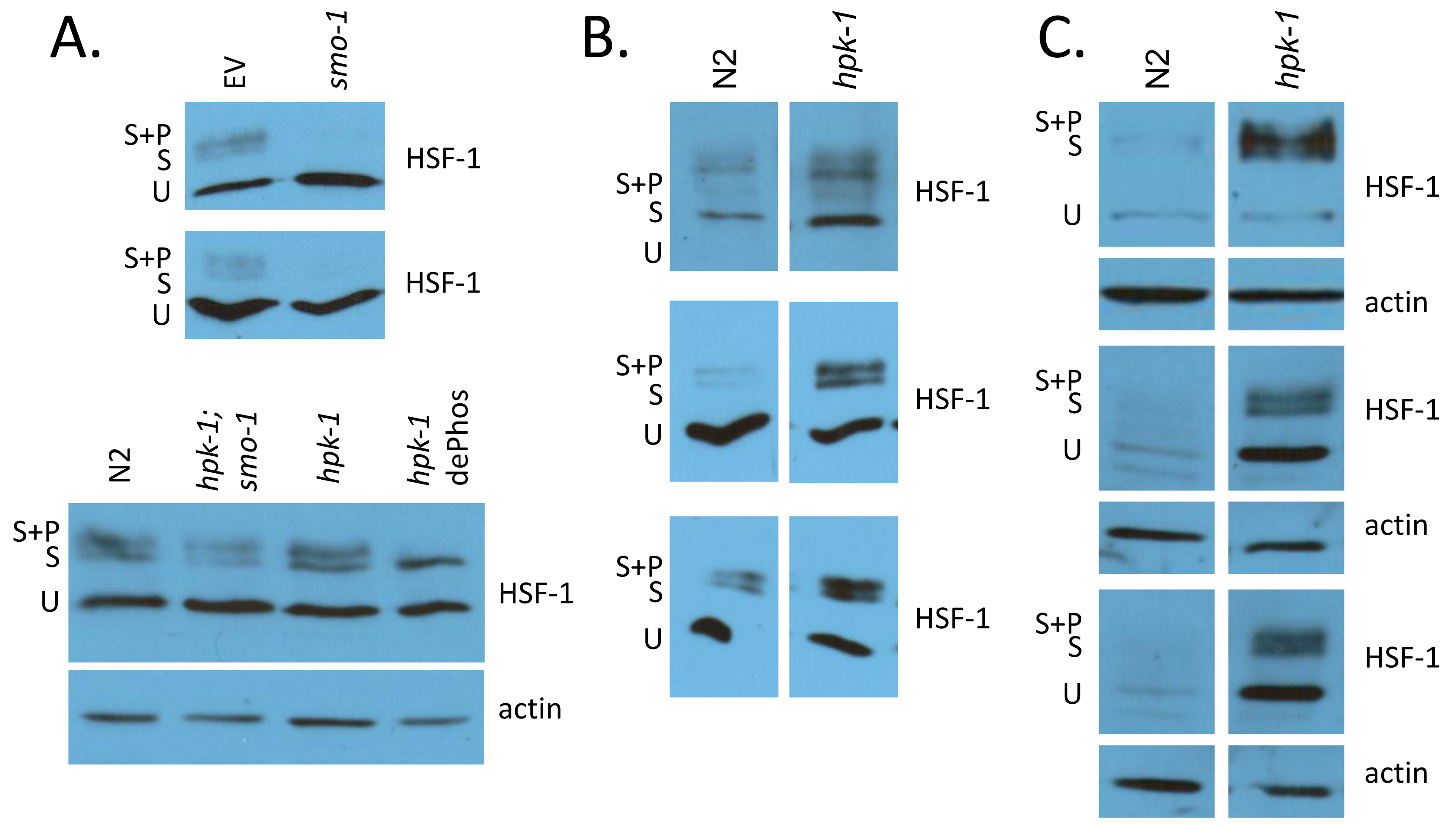

Supplement: S3 Fig — (A) Inactivation of the SUMO moiety smo-1 by RNAi prevents formation of higher molecular weight isoforms of HSF-1. Data supports Fig 6A. RNAi knockdown of smo-1 showed some variability in the degree of effectiveness, but generally decreased the relative proportion of higher molecular weight isoforms of HSF-1. Relative proportion of modified HSF-1 shown in the lower panel is 0.49, 0.35, and 0.77 for N2/ev, hpk-1(pk1393)/smo-1, and hpk-1(pk1393)/ev, respectively. ev = empty vector RNAi treatment (L4440). dePhos is λ protein phosphatase treatment. β-actin serves as a loading control. (B, C) Additional replicates for quantification of the ratio of modified to unmodified HSF-1 are shown in Fig 6B. For (B) no β-actin control was available, which precluded quantifying differences in overall expression levels between samples. (C) Additional replicates for quantifying differences in overall HSF-1 levels between N2 and hpk-1(pk1393) shown in Fig 6C. β-actin serves as a loading control. In all cases U = unmodified (75 kD), S = sumoylated (~90 kD), and S+P = sumoylated and phosphorylated (~95 kD) isoforms of HSF-1. (TIF) [file pgen.1007038.s003.tif]

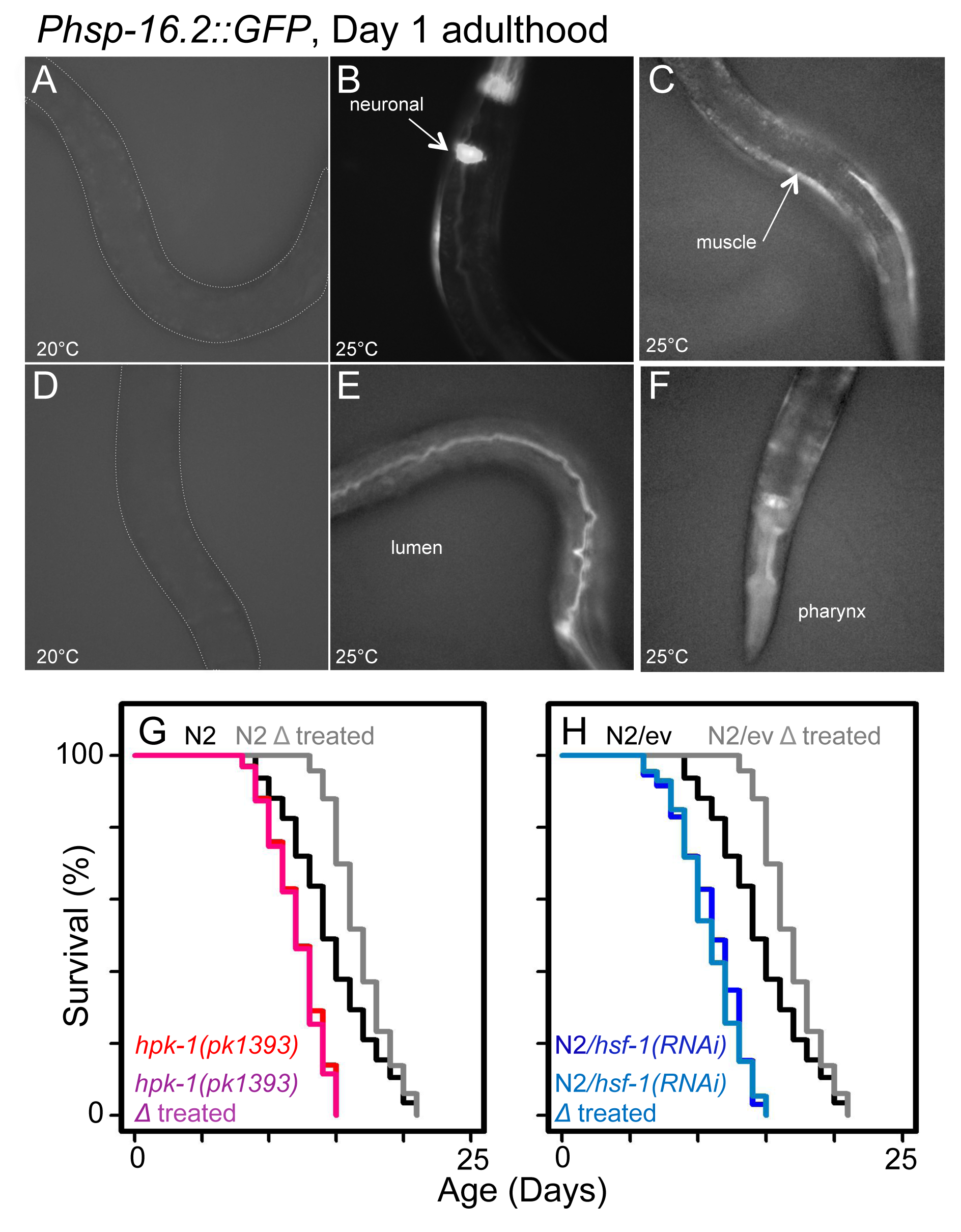

Supplement: S4 Fig — (A, B) Phsp-16.2::GFP is not expressed at 20°C (basal conditions) in day one adult animals. Outlines of animals are traced in white. (C, D, E, F) Phsp-16.2::GFP is induced heterogeneously across tissues after continued exposure to mild temperature stress of 25°C in day one adult animals. (G, H) Transient hormetic exposure to 25°C increases mean lifespan (gray versus black), which is dependent on both hpk-1 (G, red) and hsf-1 (H, blue). See S1 Table for comprehensive lifespan data. (TIF) [file pgen.1007038.s004.tif]

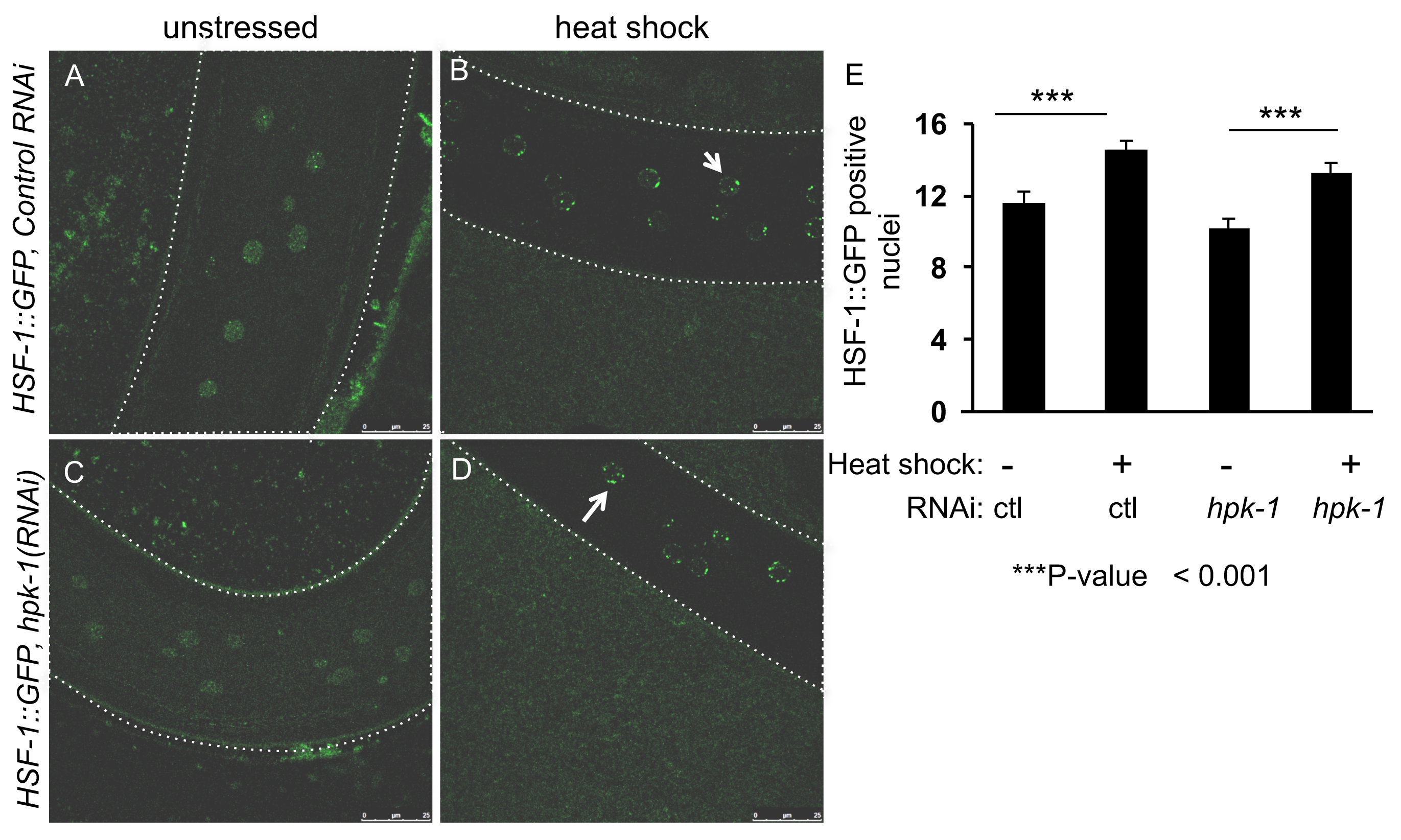

Supplement: S5 Fig — (A) Strain expressing a single copy of HSF-1::GFP is diffusely localized in the nucleus under basal conditions. (B) Strain expressing a single copy of HSF-1::GFP forms nuclear stress granules after heat shock. (C-D) Strain expressing a single copy of HSF-1::GFP treated with hpk-1(RNAi) does not alter HSF-1 basal localization (C) or formation of stress granules after heat stress (D). Results were consistent across three independent trials; approximately 20 animals were visualized per trial and all animals produced nuclear stress granules regardless of hpk-1 status. (E) Strain with low copy HSF-1::GFP shows increased nuclear accumulation after heat stress (column 1 to 2), which is not altered in the absence of hpk-1 (compare 3 to 4). Two independent trials produced similar results, with 10 animals per condition in each trial. Graph includes results from one trial (*** p <0.001, Student’s t-test). (TIF) [file pgen.1007038.s005.tif]

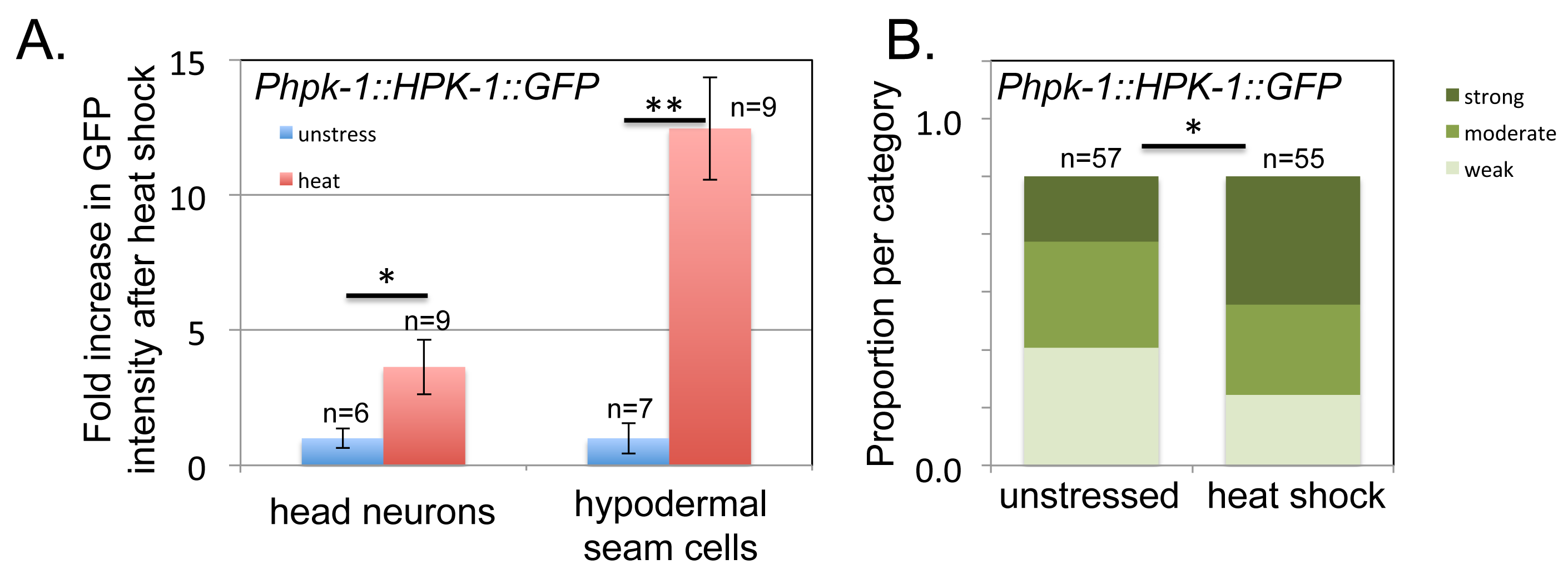

Supplement: S6 Fig — (A) Mean GFP intensity was measured in ImageJ for: head neurons, 2–4 hypodermal seam cells, and 4 spots of internal non-neuronal, non-seam cell background fluorescence for the indicated number of animals (n). Head and seam cell GFP intensity was normalized to average background fluorescence, and the contribution of background fluorescence was subtracted. Mean and standard deviation of head neuron and seam cell fluorescence in unstressed and heat shocked animals was calculated, and normalized to unstressed animals. * indicates p<0.0005, ** p<0.0002 (Student’s t-test). (B) As an independent verification of increased fluorescence within head neurons, we applied a matrix for assessing GFP levels via double-blind visualization, which allowed us to score a larger cohort of animals +/- heat shock. GFP expression was scored as “strong”, “moderate”, or “weak”. * p<0.03 (Mann-Whitney). (TIF) [file pgen.1007038.s006.tif]

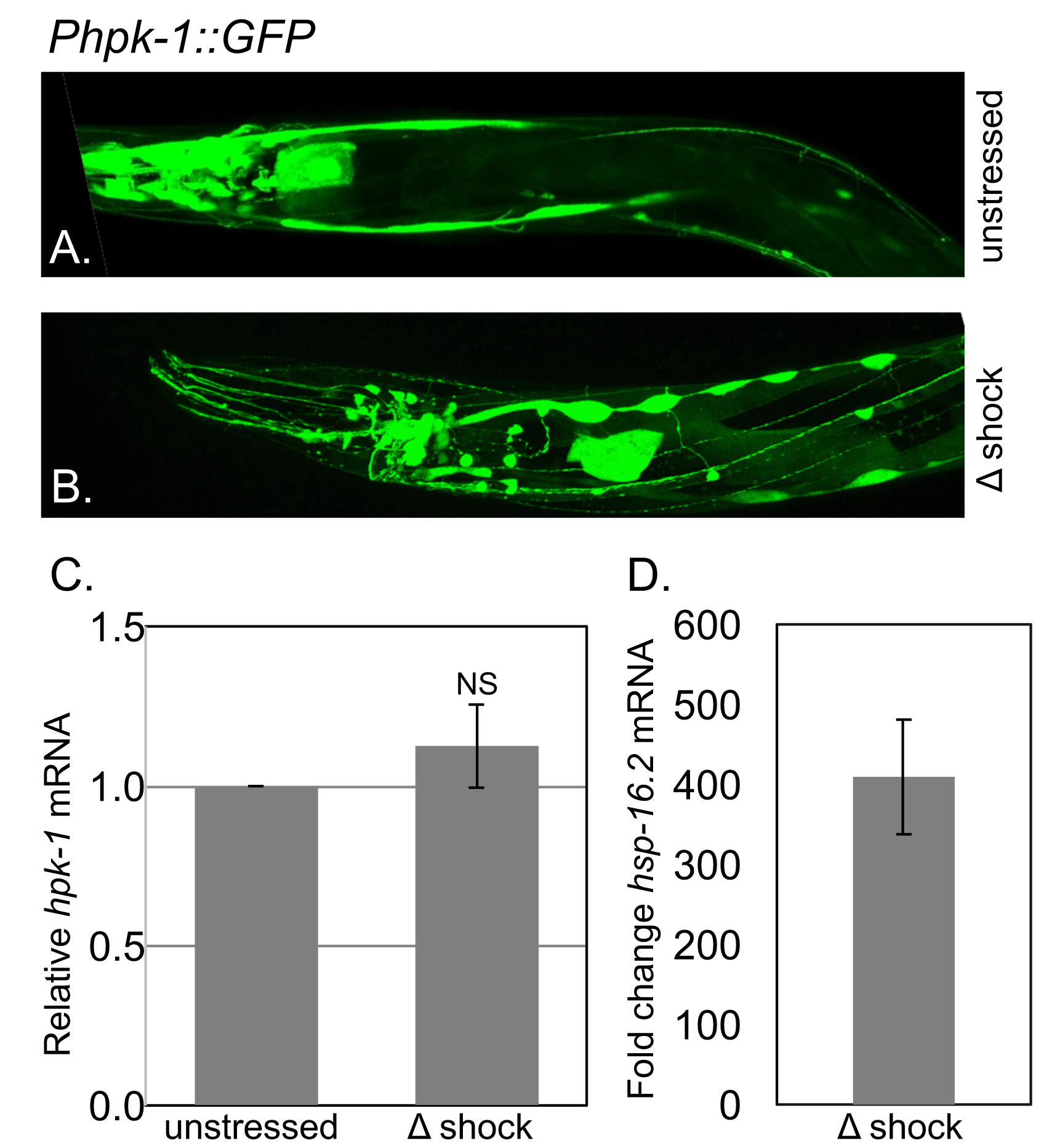

Supplement: S7 Fig — (A-B). Phpk-1::GFP expression in L4 animals under unstressed conditions (A) or after 1 hour heat shock (B). For additional images see S1 File. (C) Endogenous hpk-1 mRNA levels of L4 wild-type unstressed animals or following a 15-minute recovery after 1 hour of heat shock at 35°C. (D) Endogenous hsp-16.2 mRNA levels are induced following a 15-minute recovery after 1 hour of heat shock (p<0.05, Student’s t-test). Fold change is relative to unstressed N2. Note: mRNA from the same extracts was used in (C) and (D). In all cases expression is normalized to act-1 mRNA and error bars represent the standard deviation of a minimum of three replicates in each of two independent trials. (TIF) [file pgen.1007038.s007.tif]

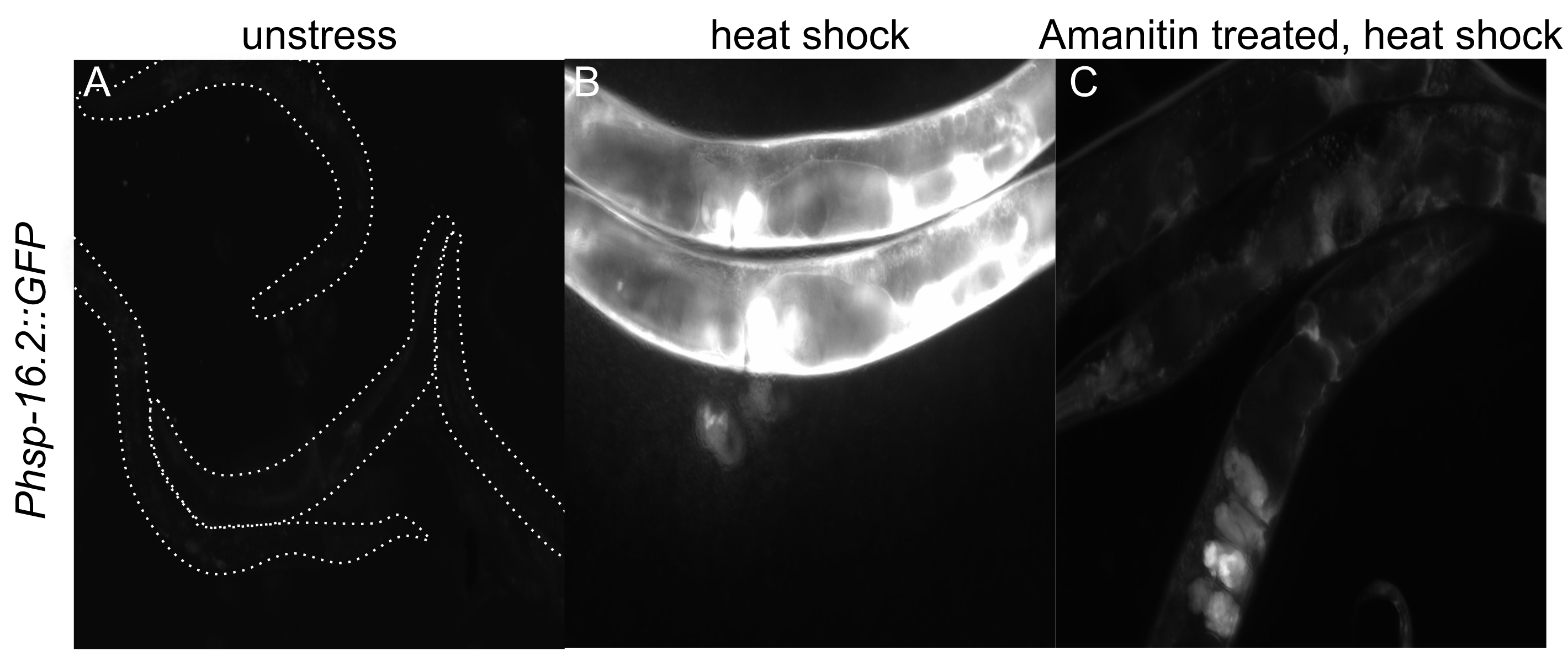

Supplement: S8 Fig — (A-C) Expression of Phsp-16.2::GFP animals under the following conditions: unstressed (A), 2 hours of recovery following heat shock for 1 hour at 35°C heat shock (B), and heat shock as in (B) but with prior treatment with 100 ug/mL α-amanitin treatment (C) (as described in [112]). Note, images in (B) and (C) were taken at a similar magnification, which was higher than image (A). Outlines of animals are traced in white. (TIF) [file pgen.1007038.s008.tif]

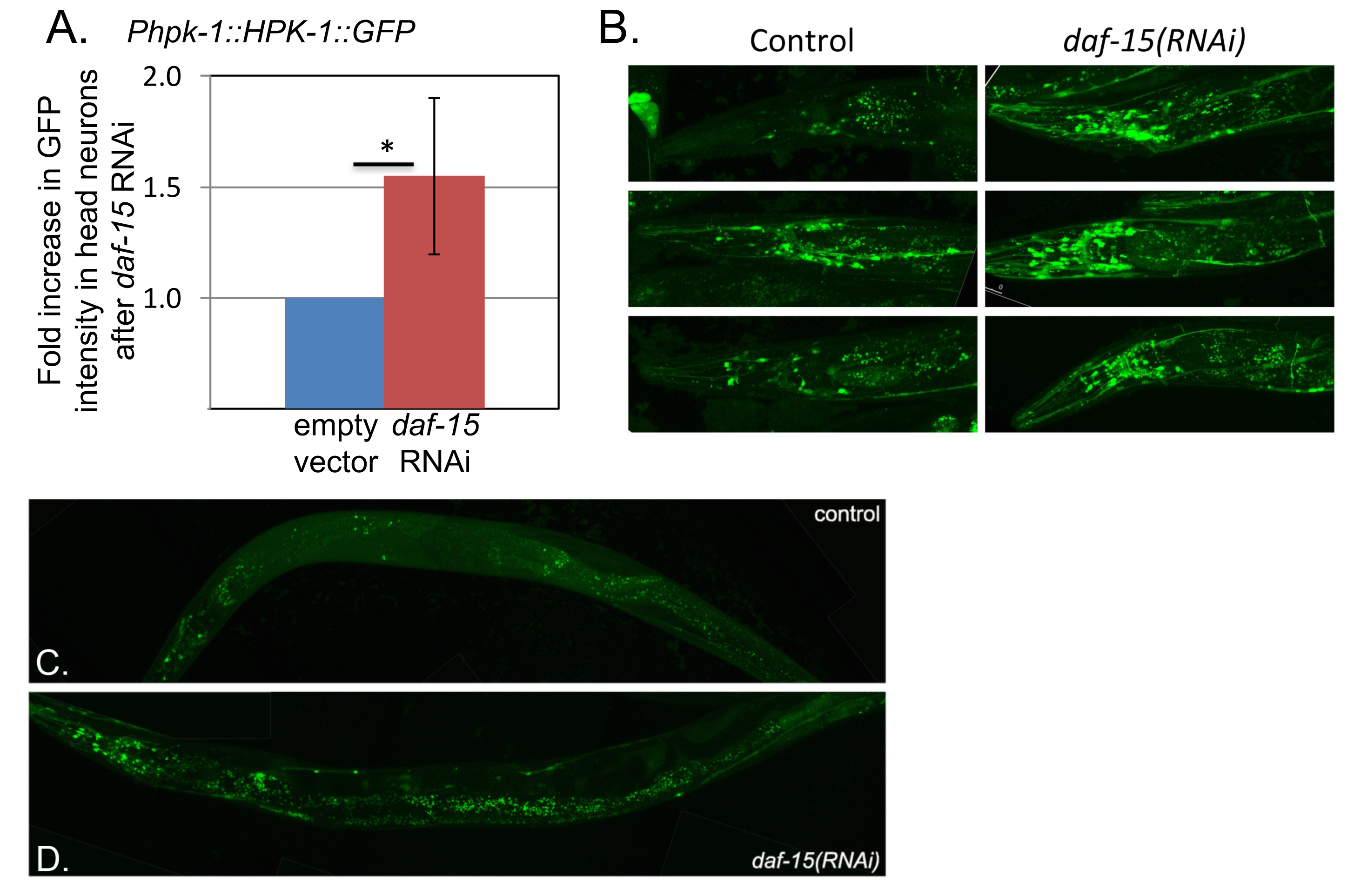

Supplement: S9 Fig — (A) Mean GFP intensity within head neurons was measured in ImageJ for Phpk-1::HPK-1::GFP expressing animals after empty vector or daf-15(RNAi)-treatment. Change in expression was normalized to empty vector; error bars represent the standard deviation of six animals. * p<0.03 (Student’s t-test). (B-D) Additional images of Phpk-1::HPK-1::GFP expression after empty vector control or daf-15(RNAi) treatment. Expression of Phpk-1::HPK-1::GFP is only induced within neurons after daf-15 (Raptor) inactivation compared to control. While expression within hypodermal seam cells varied between animals, no differences in expression between empty vector and daf-15 RNAi could be discerned, which is in contrast to heat shock. Images in (C) and (D) were stitched together from several overlapping high-resolution images and white space was artificially filled. (TIF) [file pgen.1007038.s009.tif]

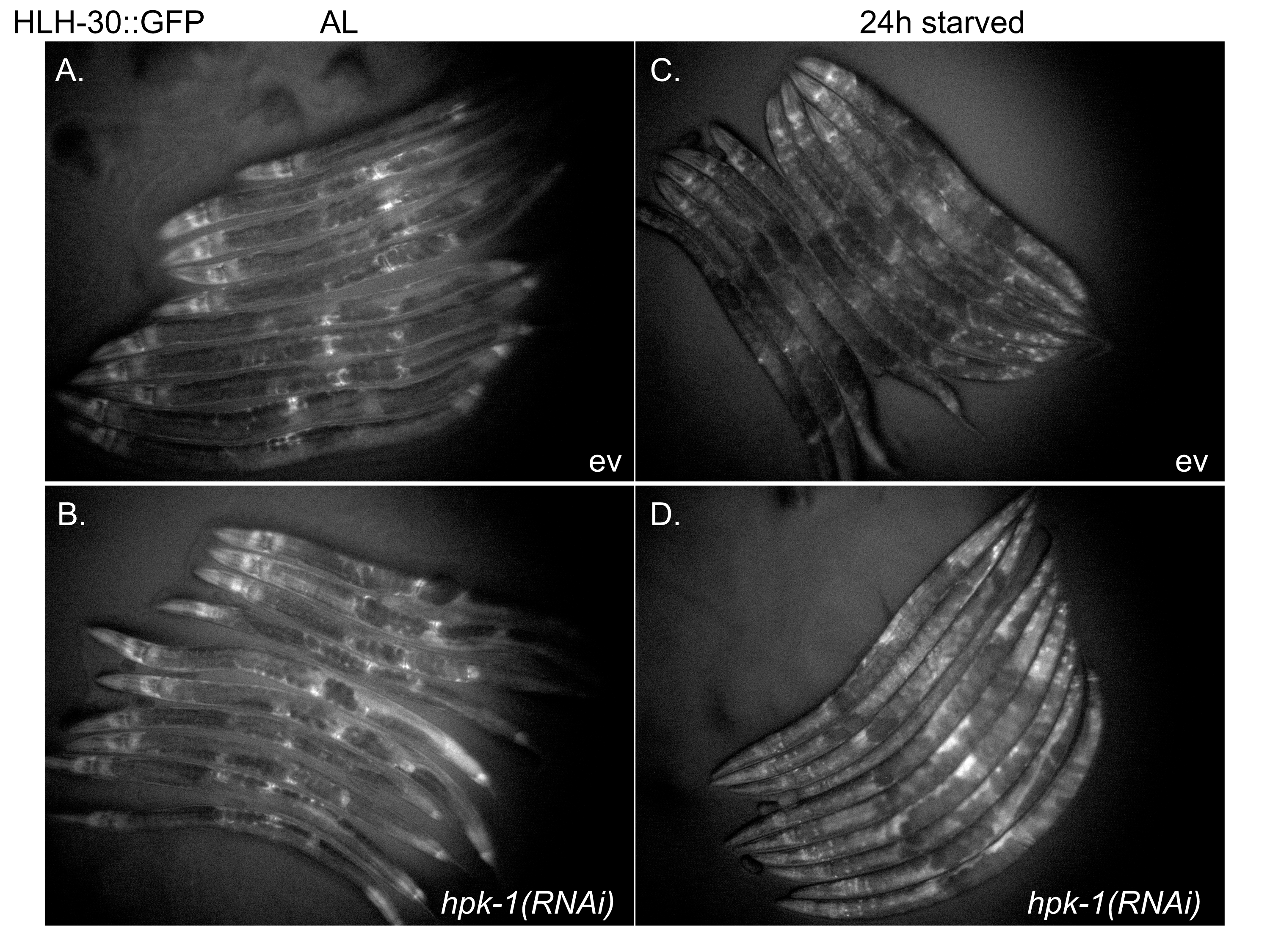

Supplement: S10 Fig — Phlh-30::HLH-30::GFP animals were treated with either empty vector (A, C) or hpk-1(RNAi) (B, D) and then were placed under ad libitum or bacterial deprivation conditions for 24 hours and HLH-30::GFP subcellular localization was assessed. (TIF) [file pgen.1007038.s010.tif]

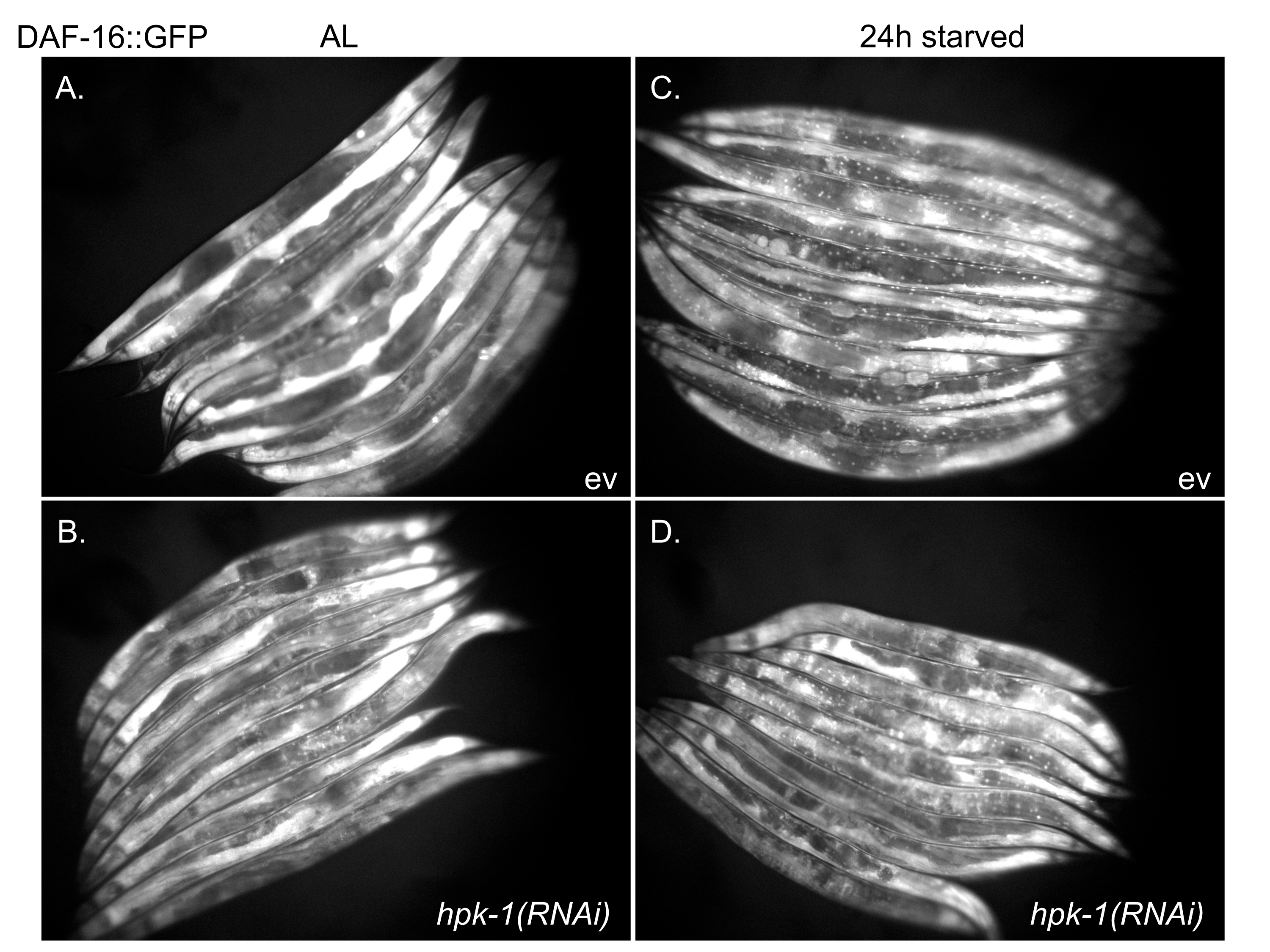

Supplement: S11 Fig — Pdaf-16::DAF-16::GFP animals were treated with either empty vector (A, C) or hpk-1(RNAi) (B, D) and then were placed under ad libitum or bacterial deprivation conditions for 24 hours and DAF-16::GFP subcellular localization was assessed. (TIF) [file pgen.1007038.s011.tif]
