# Supplementary material for: The homeodomain-interacting protein kinase HPK-1 preserves protein homeostasis and longevity through master regulatory control of the HSF-1 chaperone network and TORC1-restricted autophagy in Caenorhabditis elegans
Source: PLoS Genet. 2017 Oct 16;13(10):e1007038. doi: 10.1371/journal.pgen.1007038 (PMC5658188; doi:10.1371/journal.pgen.1007038)
Supplement: S1 File — (PDF) [file pgen.1007038.s017.pdf]

Supplemental File 1: Additional images of AVS393 (*Phpk-1::GFP*) under unstressed condition or after heat stress

Unstressed

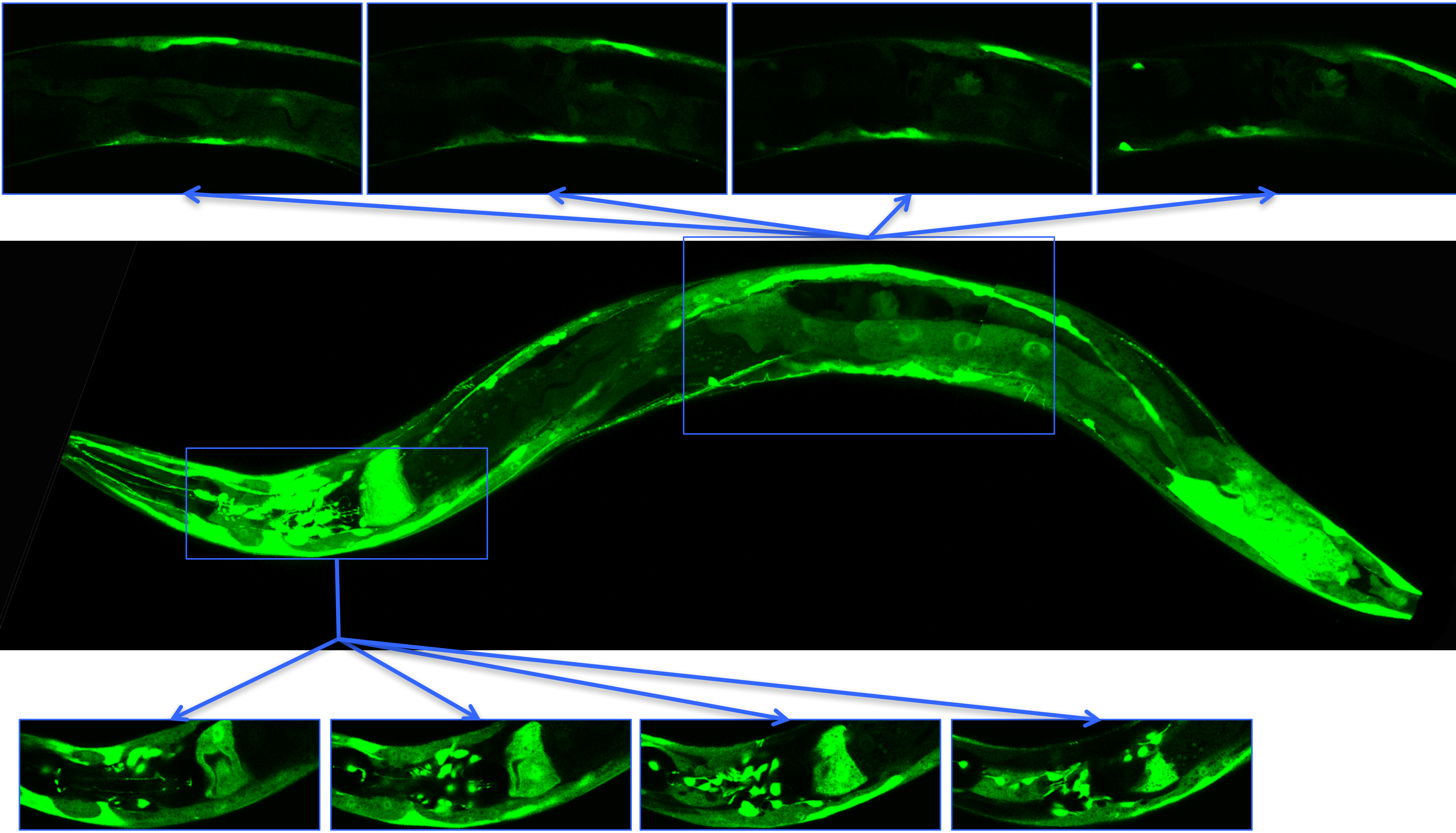

Middle: composite of three images with all stacks (max).
Top/Bottom: Individual z stack of highlighted regions

AVS393: *artEx11[Phpk-1::GFP+ pRF4(rol-6(su1006))]*

<u>Unstressed</u>

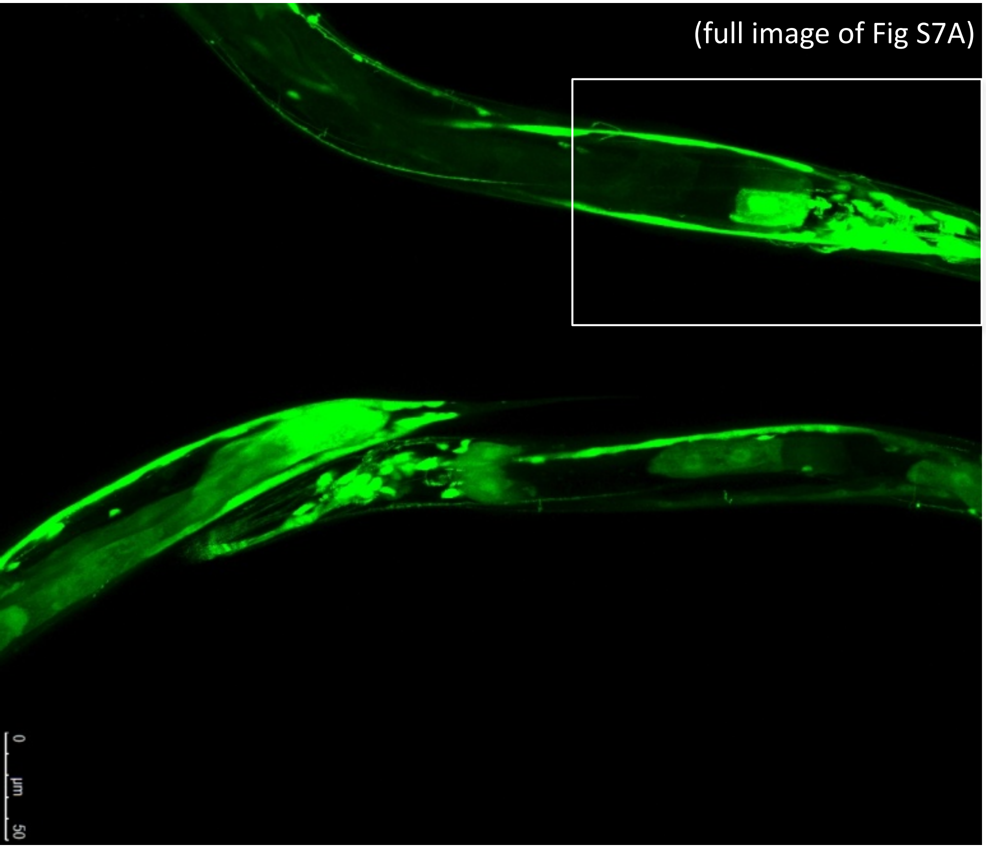

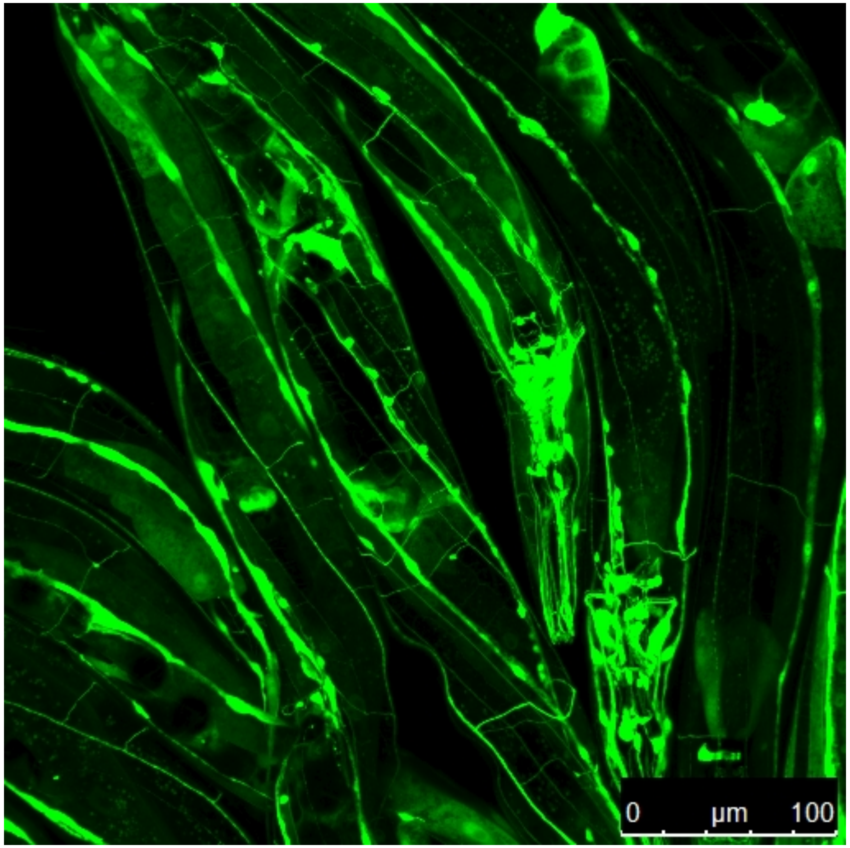

AVS393: *artEx11[Phpk-1::GFP+ pRF4(rol-6(su1006))]*

Heat shock

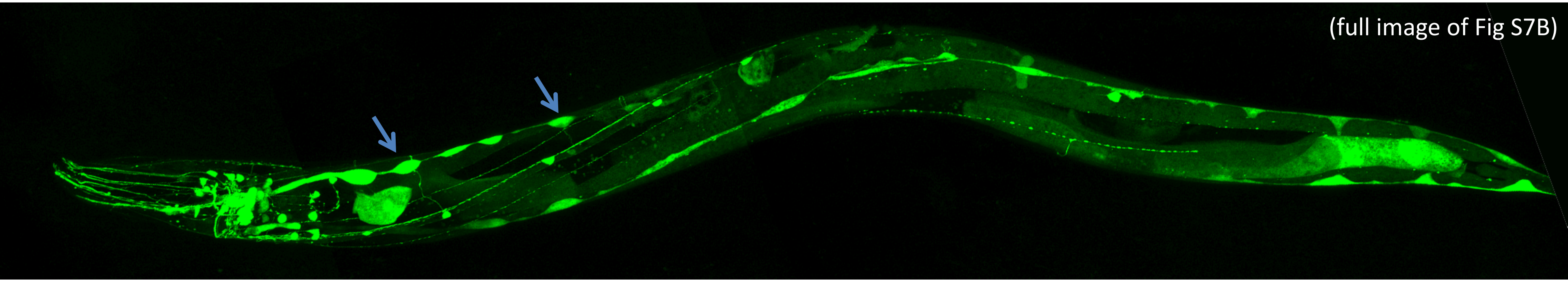
(full image of Fig S7B)

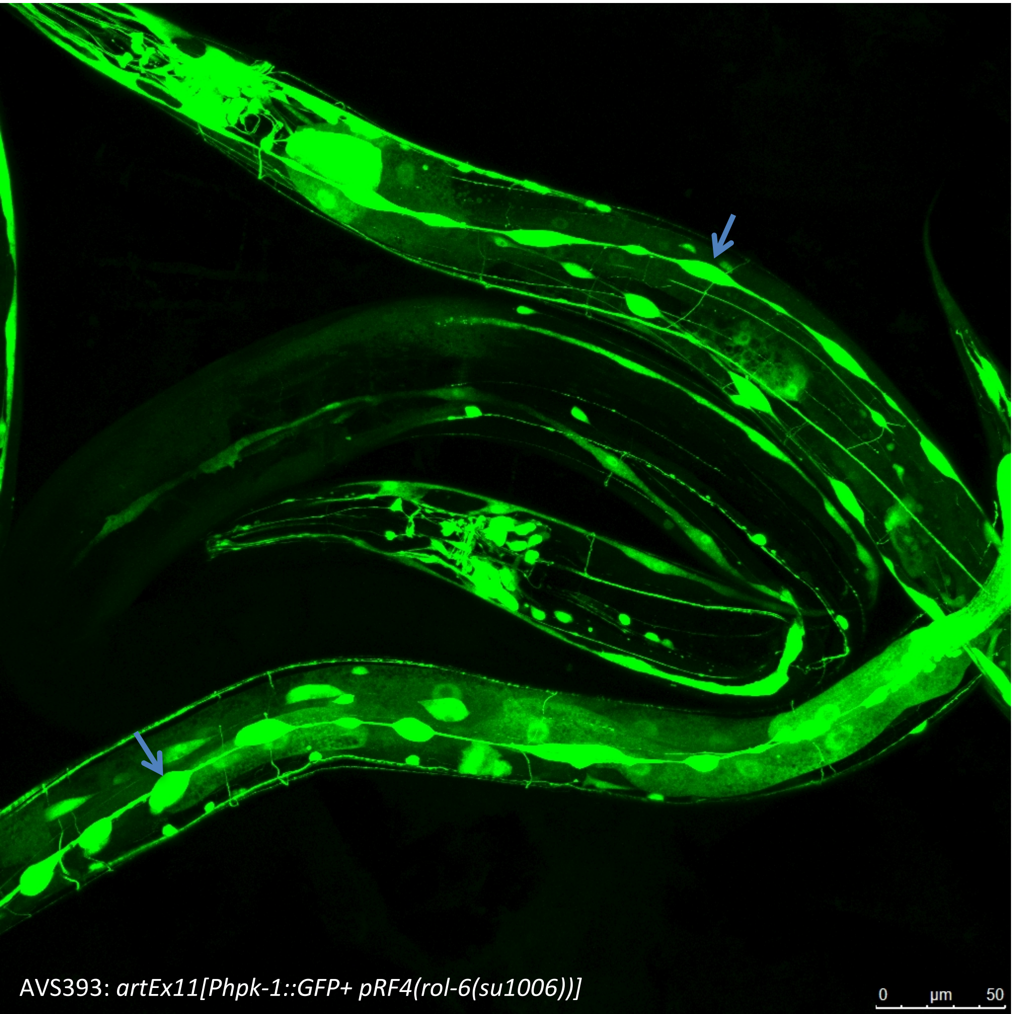
AVS393: *artEx11[Phpk-1::GFP+ pRF4(rol-6(su1006))]*
0 µm 50

- Under unstressed conditions *Phpk-1::GFP* expression is within hypodermal seam cells (1st slide).
- After heat shock *Phpk-1::GFP* expression reveals that seam cells appear larger and swollen (2nd slide).
- Animals appear "twisted" in images due to the *rol-6* mutation

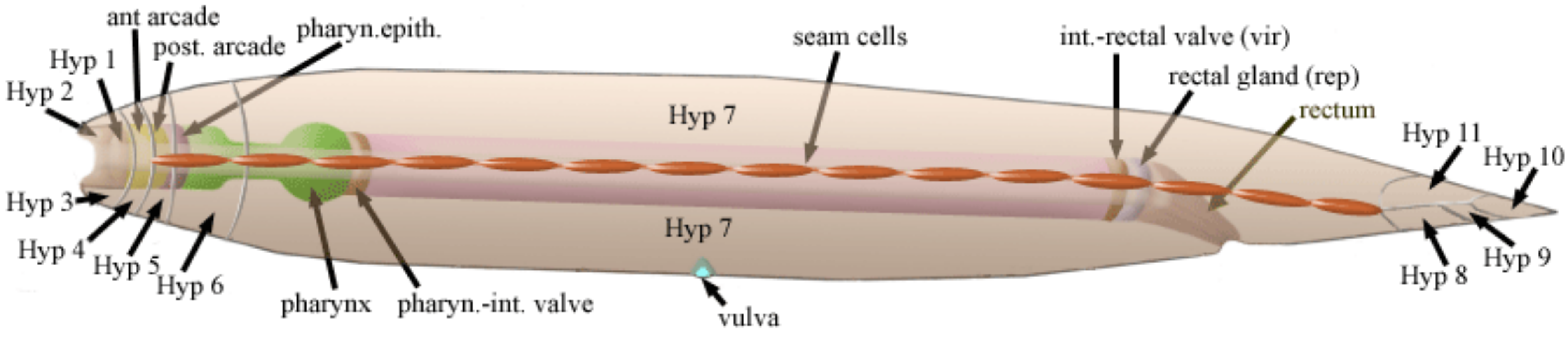

Hypodermis in the adult hermaphrodite

Image from: WormAtlas. Altun, Z. F. and Hall, D. H. (ed.s). 2002-2006.
http://www.wormatlas.org/ver1/handbook/hypodermis/hypodermis.htm
